# ΔNAzyme: A Foundational QM/MM Δ‑Machine Learning Potential for Nucleic Acid Enzyme Catalysis

**DOI:** 10.1021/acs.jctc.6c01635

**Published:** 2026-10-12

**Authors:** Erika McCarthy, Timothy J. Giese, Şölen Ekesan, Julie Puyo-Fourtine, Lauren Lerew, Andreas W. Götz, Darrin M. York

**Affiliations:** † Laboratory for Biomolecular Simulation Research, Institute for Quantitative Biomedicine and Department of Chemistry and Chemical Biology, 151434 Rutgers University, Piscataway, New Jersey 08854, United States; ‡ San Diego Supercomputer Center, 8784 University of California San Diego, La Jolla, California 92093-0505, United States

## Abstract

Nucleic acid enzymes (NAzymes), including ribozymes, DNAzymes, and XNAzymes, are programmable biocatalysts that catalyze diverse chemical reactions and provide unique platforms for investigating catalysis and engineering molecular function. Their central roles in biology and evolution, together with emerging applications in biotechnology, synthetic biology, and medicine, have created an urgent need for predictive computational design frameworks. Although multiscale computational enzymology integrating *ab initio* quantum mechanics can, in principle, provide predictive insight into these systems, current approaches remain computationally prohibitive for large, complex NAzymes and high-throughput design workflows. Machine learning potentials have the potential to enhance the accuracy and efficiency of multiscale modeling approaches, but QM/MM contexts in which the MM environment is highly polarizing remain challenging. Nucleic acid enzymes present a particularly demanding case: experimentally relevant predictions require an accuracy that semiempirical QM/MM methods alone cannot reliably deliver in their often large, highly charged, metal-dependent active sites. Building on the range-corrected QM/MM Δ-machine learning potential (ΔMLP) framework, we develop ΔNAzyme, a foundational ΔMLP for nucleic acid enzyme reactions. We generate a training set of QM/MM simulations spanning diverse nucleic acid enzyme classes, with energies and forces computed at both the reference (DFTB3) and target (PBE0/6–31G*) levels of theory, yielding a correction on top of DFTB3 that recovers PBE0 quality results. We train models using both the Deep Potential and MACE architectures. The MACE model exhibits enhanced transferability, which we attribute to the equivariant, many-body message-passing framework. Free energy profiles from case-study reactions validate ΔNAzyme against target-level results and show that the model supports efficient fine-tuning to new nucleic acid enzyme systems with minimal additional data.

## Graphical abstract

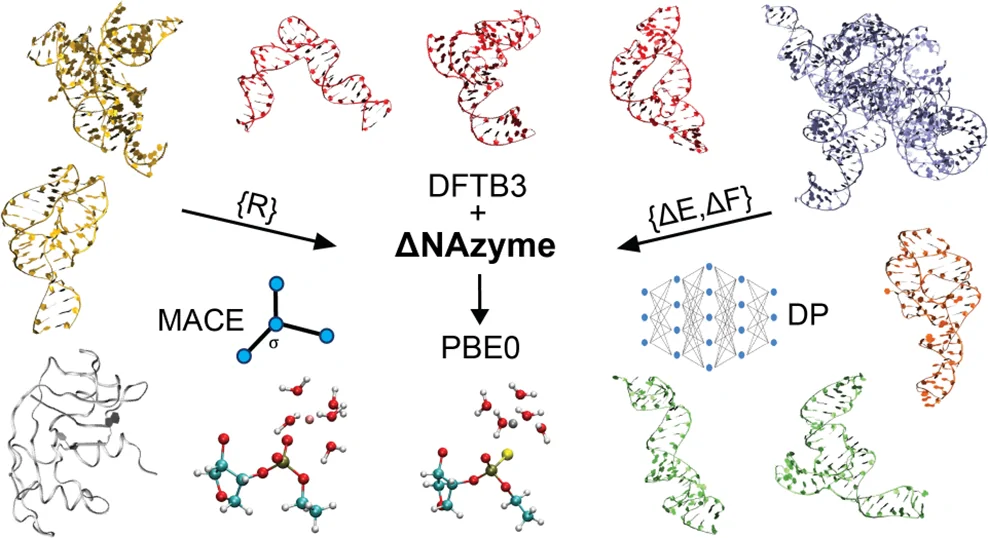

## Introduction

Nucleic acid enzymes, or NAzymes (e.g., ribozymes, DNAzymes, and XNAzymes), catalyze diverse chemical reactions^1–3^ and have emerged as powerful tools in biotechnology,^4–6^ therapeutics,^7–9^ and synthetic biology.^10,11^ Small self-cleaving ribozymes, of which there are three major known classes, are important model systems for elucidating fundamental catalytic strategies,^12,13^ while larger ribozymes such as the *Tetrahymena* group I intron^14–16^ further demonstrate the ability to employ sophisticated mechanisms involving conformational rearrangements, metal ion recruitment, and complex hydrogen-bonding networks.^17–21^ In parallel, engineered DNAzymes^22,23^ have been developed for gene silencing^24^ and programmable RNA-cleaving therapeutics,^25–27^ and designed alkyltransferase ribozymes, including the methyltransferase ribozyme (MTR1),^28–30^ self-alkylating ribozyme (SAR),^31,32^ and site-specific alkyltransferase ribozyme^33,34^ have emerged as platforms for *in vivo* RNA labeling and modification. More broadly, synthetic xenonucleic acids^35–38^ expand the available RNA and DNA building blocks^39,40^ and enable the development of XNAzymes^26,27,41^ with enhanced stability, nuclease resistance, and cellular activity.^41,42^

Despite their promise, the predictive design of NAzymes by computational approaches remains elusive^43^ owing to an incomplete understanding of the principles that govern catalytic efficiency, substrate recognition, and environmental responsiveness. Computational enzymology frameworks that integrate multiscale modeling at the *ab initio* quantum level and molecular simulation can, in principle, provide the mechanistic insight needed to guide design.^44–50^ Such methods have been applied successfully to small endonucleolytic ribozymes that catalyze RNA cleavage,^50^ including the hammerhead,^51,52^ hairpin,^53^ glucosamine-6-phosphate,^54,55^ twister,^56,57^ Varkud satellite,^58^ and hepatitis delta virus^59,60^ ribozymes, yielding general catalytic principles that transfer to artificially engineered DNAzymes.^12,13,61^ Their extension to larger, more complex RNA catalysts such as RNase P,^62,63^ group I and II introns,^64^ and molecular machines such as the ribosome^65^ and spliceosome^66–68^ has, however, been limited in scale.^69^ Further, current *ab initio* computational enzymology approaches are too resource and time intensive to be routinely applied in high-throughput nucleic acid design workflows. Taken together, these limitations motivate the development of new technologies to address the unique challenges these systems present.

*Challenges for Multiscale Modeling of NAzyme Catalysis*: NAzymes pose major challenges for multiscale modeling due to conformational heterogeneity, large and highly charged metal-dependent active sites, and complex electrostatics that are strongly modulated by solvation and the surrounding ion atmosphere.^61,70,71^ Computing the reaction pathways and free energy barriers that provide predictive mechanistic insight requires an accurate quantum mechanical (QM) treatment, with rigorous long-range electrostatics, together with intensive sampling of multidimensional free energy surfaces.^72^ *Ab initio* QM methods are too computationally intensive for routine application to such systems, whereas fast, approximate semiempirical QM methods such as density functional tight binding^73–77^ often lack the chemical accuracy needed for quantitative prediction.^78^

Machine learning potentials (MLPs) have recently been used effectively to accelerate nonenzymatic quantum mechanical/molecular mechanical (QM/MM) reactions involving proton transfer^79^ and phosphate chemistry,^80–83^ fundamental components of NAzyme reactions. One strategy that has proven particularly effective is to develop hybrid QM/MM+ΔMLP models^80,84–88^ that use a fast base QM model and train a MLP to correct its energy and forces to the *ab initio* level.^89,90^ Because it departs from a robust base QM model, this strategy retains built-in physics such as chemical bonding, charge and spin polarization, and long-range electrostatics with the MM environment, while the ΔMLP corrects only the short- and mid-range interactions with negligible computational overhead. Such corrections have been realized with kernel methods such as Gaussian process regression (GPR),^87,91,92^ deep neural networks (DNN),^93–100^ and graph neural networks (GNN)^85,101–104^ and have been extended to multiple-time-step QM/MM^105^ and multilevel free energy schemes.^106–108^ Currently, ΔMLP corrections have advanced QM/MM simulations of enzymatic reactions in solution, including chorismate mutase,^86^ dihydrofolate reductase,^88^ the cyclooxygenases,^109^ and MTR1,^110^ but the focus has predominantly been limited to single reactions.

Foundational models such as MACE-OFF,^111^ ANI-2x,^112^ AIMNet2,^113^ DPA-2,^114^ PhysNet,^115^ MEGNet,^116^ OMNI-P1,^117^ FeNNix-Bio1,^118^ and QDπ ^80,119^ achieve near *ab initio* accuracy for organic molecules and materials but are not ideal for NAzymes. Most foundational MLPs are trained on neutral, gas-phase organic molecules and neglect the QM–MM interactions and the metal chemistry (Mg^2+^, Zn^2+^, Ca^2+^) that dominate NAzyme catalysis, so their transferability across the strongly varying electrostatic environments of nucleic acid active sites is limited. Correcting the internal QM interactions alone is also insufficient, because the *ab initio* electron density differs from that of the base QM model in ways that extend into the MM environment.^84^

To address this, we previously developed a range-corrected machine learning framework in which the neural network smoothly adjusts both the QM–QM and QM–MM interactions out to an optimal cutoff of 6 Å,^84,120^ implemented for both DNNs and GNNs within a new software infrastructure in DeePMD-kit^121,122^ and AMBER.^123–125^ In the present work, we integrate these methods into a framework for training a foundational QM/MM+ΔMLP for nucleic acid catalysis that we designate ΔNAzyme.

Data sets representing nucleic acid-catalyzed reactions and their enzymatic environments do not currently exist, and the number of high-resolution NAzyme structures is limited. Therefore, in this work, we assemble a training set spanning diverse NAzyme classes and chemistries, with energies and forces evaluated at a semiempirical reference (DFTB3) and an *ab initio* target (PBE0/6–31G*) level, which we refer to as the NAzyme data set (version 1.0).

We train ΔNAzyme models using both the Deep Potential^122^ and the equivariant MACE^126^ architectures, which we henceforth refer to as ΔMACE and ΔDP, respectively. We show that the equivariant ΔMACE models are more transferable across active site environments, that they support efficient fine-tuning to new NAzyme systems from minimal additional data, and that the foundational model reproduces *ab initio* free energy profiles for case-study reactions. ΔNAzyme thus provides an accurate, transferable, and rapidly extensible starting point for the mechanistic study and design of nucleic acid catalysis, intermediate between universal potentials and specific reaction parametrizations (SRPs).

The remainder of the manuscript is organized as follows. The Methods section describes the relevant background, the range-corrected ΔMLP approach in the context of DNNs and GNNs, generation of the NAzyme data set, and the data collection, training, validation, and simulation protocols. The Results and Discussion section motivates the need for a NAzyme-specific QM/MM+ΔMLP approach, establishes the choice of reference method, evaluates the ΔDP and ΔMACE models on training and on experimentally relevant test sets, and demonstrates how the foundational ΔNAzyme model serves as a departure point for active learning and transfer learning to new reactions.

## Methods

### Background

Semiempirical QM/MM methods enable long time scale reaction sampling but are often too inaccurate to guide experiments, whereas *ab initio* QM/MM is 100–1000 times more expensive.^84^ A ΔMLP bridges this gap by correcting a fast reference potential to a target *ab initio* level while retaining the built-in physics of the base QM model.^84,89^ For NAzymes, the correction must also capture QM–MM interactions: catalysis occurs at highly charged, metal-dependent active sites,^61,127^ where most existing potentials, trained on neutral organic molecules and lacking biologically significant divalent metal ions, are inadequate.

We train range-corrected ΔMLPs using two architectures: the Deep Potential range-correction (DPRc) descriptor implemented in DeePMD-kit^84,128,129^ and the equivariant MACE model^126^ accessed through the DeePMD-GNN plugin.^122^ Both correct DFTB3 to the PBE0/6–31G* level and, unlike SRPs, are trained for domain wide accuracy; we designate the resulting models, ΔNAzyme models. Because no data set of NAzyme reactions and environments currently exists, we curate one that draws on, and expands upon, over two decades of simulation work from our group, totaling approximately 369,000 structures across 11 systems and 46 reaction mechanisms; we refer to it as the NAzyme data set (version 1.0).

### The Range-Corrected ΔMLP Approach

Range-corrected^84^ ΔMLP models based on the Deep Potential^121^ and MACE^130^ architectures were trained to correct DFTB3/3ob^74,75,131^ QM/MM energies and forces to reproduce PBE0/6–31G* QM/MM.^132,133^ The ΔMLP is a nonelectronic correction to the potential.

1 $$E\left(R;P\right)=E_{\mathrm{QM}}\left(R;P\right)+E_{\mathrm{QM}/\mathrm{MM}}\left(R;P\right)+E_{\mathrm{MM}}\left(R\right)+E_{\mathrm{ML}}\left(R\right)$$

*E*_ML_(**R**) is the ΔMLP energy, **R** is the atomic coordinate matrix, and **P** is the QM method’s single-particle density matrix. The range-corrected formalism modifies both the QM–QM and QM–MM interactions within a smooth cutoff (*r*_cut_) of the QM region. The ΔMLP must be designed with several attributes to produce energy corrections that are free of discontinuities. First, the correction should not alter the MM–MM interactions because it is applied only to the QM region and its interaction with nearby MM atoms. In the MACE model, this means that the graph neural network excludes edges connecting MM atoms to other MM atoms. Similarly in the Deep Potential model—specifically, we use the two-body embedding smooth edition of the Deep Potential descriptor—the “embedding network” parameters are indexed by the chemical species pair. The MM elements are considered to be different chemical species from the corresponding QM elements. In this way, the QM–MM correction between 2 hydrogens can be different from if they were both QM hydrogens, for example. Second, the ΔMLP should not contain an energy bias for the MM atoms. An energy bias is a size-extensive constant added to the energy for each atom. It must be zero for MM atoms, because they may diffuse away from the QM region and therefore no longer be included in the ΔMLP calculation. Finally, the pairwise descriptors must smoothly approach zero as an MM atom approaches a cutoff from the QM region. In the Deep Potential model, a switching function is explicitly included in the “coordinate matrix”. The MACE model similarly applies a smoothing envelope to the edge descriptors.^120^ We use a 6 Å cutoff in both models.

### Deep Potential Hyperparameters

The DeepPot-SE DPRc model has been previously described several times.^81,84^ In brief, the DeepPot-SE^134^ fitting network is composed of 3 hidden layers using 240 neurons/layer, and the output is a scalar correction to the energy. The filtering network is composed of 2 hidden layers using 25 and 50 neurons, and the output is 100 neurons of the “embedding matrix”. The reduced dimension embedding matrix stored the first 12 columns of the embedding matrix. The “coordinate matrix” (the input layer of the filtering network) scales the inverse distances between the QM and MM atoms, such that the neural network correction smoothly approaches zero at a cutoff distance of 6 Å. Each layer was activated with the hyperbolic tangent function. The resulting model is referred to as ΔDP.

### MACE Potential Hyperparameters

The MACE potential^111,126^ is a graph neural network that we also trained using the range-corrected formalism,^120^ which we refer to as ΔMACE. The graph edges consist of all QM–QM and QM–MM atomic pairs within 6 Å of each other. The edge features are expanded into a basis of third-order spherical harmonics and 8 radial Bessel functions scaled by a fifth-order polynomial that smoothly forces the features to zero as the separation reaches the 6 Å cutoff. The edge features are input to a 3-layer multilayer perceptron (MLP) containing 64 neurons/layer. Equivariant message passing between atoms was performed with spherical harmonics of order 1 yielding feature vectors with 128 embedding channels unless otherwise specified. A correlation order (body order minus 1) of 3 was used, yielding 4-body to balance speed and accuracy. Atomic embeddings were updated after 2 layers of message passing. The final atomic energy contribution is computed by passing the readout of the last layer to a perceptron with 16 channels gated by the smooth sigmoid linear unit (SiLU) activation function. The hyperparameters are in line with those of the so-called MACE-OFF medium model from ref 111. An additional version of the model was trained using 256 embedding channels with all other parameters held fixed, denoted ΔMACE-L.

### Generation of a Data Set Representing Diverse Nucleic Acid Chemistry

Training a foundational ΔMLP for QM/MM simulations of NAzymes requires curation of a data set that captures the diverse, highly charged environments characteristic of NAzyme active sites. Gas-phase or aqueous fragment data common in publicly available data sets^135–140^ of organic molecules or materials are insufficient and not well suited for this purpose. In particular, the data set must represent Mg^2+^ coordination by phosphates, nucleobases, sugar oxygens, and combinations thereof. To support enzyme design and mechanistic studies, the trained model must also accommodate elements commonly used in experiments probing NAzyme mechanisms, including sulfur for phosphorothioate substitutions and thiophilic metal ions such as Ca^2+^ and Zn^2+^ for metal-ion rescue experiments. Altogether, the NAzyme data set includes elements H, C, O, N, P, S, Mg, Na, Zn, and Ca in QM contexts, and H, C, O, N, P, S, Mg, Na, and Cl in MM contexts.

Systems were selected to span a wide range of NAzyme chemistry and active site scaffolds, including both naturally occurring and artificially evolved enzymes. NAzymes predominantly catalyze 2′-*O*-transphosphorylation reactions that cleave RNA, with conserved scaffolds and catalytic strategies observed across enzyme classes,^12,13,141^ defined by their general acid and base moieties. Three predominant mechanistic classes are represented: guanine (G) general base with adenine (A) general acid (G+A); G base with a divalent metal ion (M^2+^) as acid (G+M^2+^); and M^2+^ general base with cytosine (C) general acid (M^2+^+C), as illustrated in Figure 1. These classes are represented by the Varkud Satellite (VSr), Twister (TWr), Pistol (PSr), and Hammerhead (HHr) ribozymes, the 8–17 DNAzyme (8–17dz), and the Hepatitis Delta Virus ribozyme (HDVr). RNA cleavage by the protein RNase A, which employs two histidine residues (H^+^+H^+^), is included to represent RNA interactions with a protein environment. The 3′-*O*-transphosphorylation reactions involved in splicing, which require divalent metal ions in both the acid and base roles (M^2+^+M^2+^), are represented by the cleavage and ligation reactions catalyzed by the *Tetrahymena* ribozyme (THr). The growing landscape of artificially evolved alkyl transfer ribozymes is represented by the MTR1 alkyl transfer reaction and the epoxide ring-opening and alkyl transfer reaction of the self-alkylating ribozyme (SAR).

**Figure 1:**
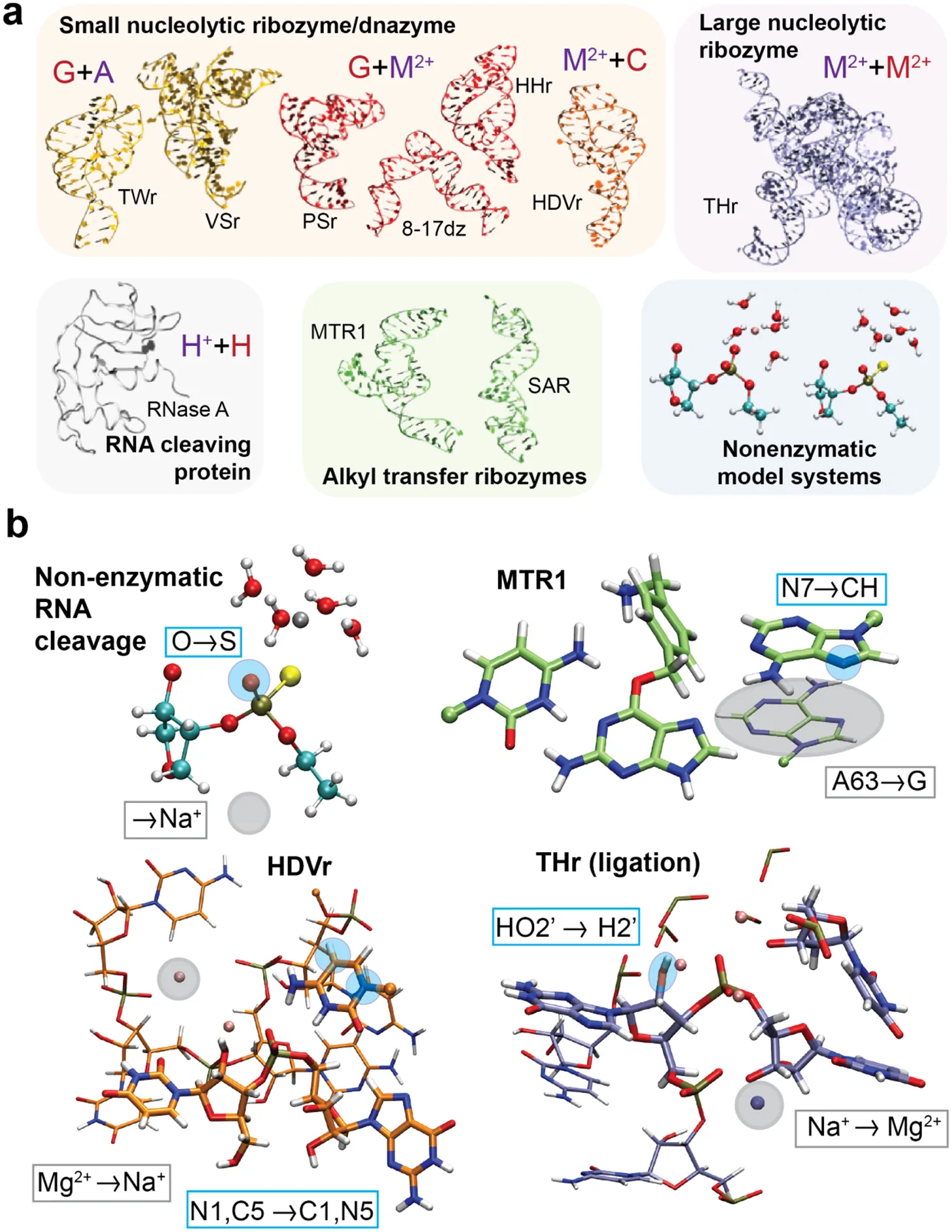
Overview of systems that comprise the NAzyme data set. (a) Classes of NAzymes and nucleic acid processing chemistry and representative systems included in the training data. (b) Systems used as test sets for semiempirical methods and for transferability tests of ΔMLP models. Modifications to the QM and MM regions used as test sets are highlighted in blue and gray, respectively.

To anticipate common experimental substitutions, a series of model nonenzymatic (NE) reactions mimicking RNA cleavage under varied conditions was also generated. These include:

- Protonation of the leaving group by metal-ion-coordinated water for Na^+^, Mg^2+^, Ca^2+^, and Zn^2+^.
- Individual substitution of the O2′, O5′, pro-*R*_*P*_, and pro-*S*_*P*_ nonbridging phosphoryl oxygens (NBOs) by sulfur, with the pro-*S*_*P*_-coordinating Mg^2+^
- pro-*S*_P_ to sulfur substitution (SpS) in the presence of Zn^2+^, mimicking a thiophilic metal ion rescue
- Protonation of the leaving group by Mg^2+^-coordinated water with indirect metal coordination to the pro-*S*_P_
- Leaving group departure without protonation, in the absence of ions, in the presence of direct or indirect Mg^2+^ coordination, and with SpS/Zn^2+^ substitution.

In total, the NAzyme data set comprises 369,000 structures across a range of charge states, as summarized in Table 1. Of these, 233,125 correspond to unique atomic compositions within *r*_cut_ (with different atomic coordinates), arising from 500 samples drawn from 16 umbrella sampling windows each per reaction pathway, along with 500 reactant state structures for systems without reaction data (TWr, 8–17dz). A sparser variant of the data set was also considered for training, comprising 139,000 structures sampled from 6 windows per reaction. These are referred to as the large (L) and small (S) data sets, respectively. The large data set is comprised of odd-numbered umbrella windows, and the small data set is a subset of the large data set including umbrella windows 1, 7, 13, 19, 25, and 31.

**Table 1:** Overview of Systems Included in the NAzyme Data Set^a^

System	N(paths)	N(RC)	N(QM)	Q(QM)	Z(QM)	Z(MM)
HDVr	5	5	93	0	H,C,N,O,Mg,P	H,C,N,O,Na,Mg,P,Cl
VSr	1	3	77	0	H,C,N,O,Mg,P	H,C,N,O,Na,Mg,P,Cl
HHr	2	3	76	–1	H,C,N,O,Mg,P	H,C,N,O,Na,Mg,P,Cl
PSr	2	1	33	–2,–1	H,C,N,O,P	H,C,N,O,Na,Mg,P,Cl
8–17dz	0	0	71	0	H,C,N,O,P,Mg,Ca,Zn	H,C,N,O,Na,P,Cl
TWr	0	0	78	–2	H,C,N,O,P	H,C,N,O,Na,Mg,P,Cl
NE	16	1,2	24–43	–2,–1,0	H,C,N,O,P,S,Na,Mg,Ca,Zn	H,O,Na,Cl
THr	10	2,3	77–93	–2,–1,0	H,C,N,O,Na,Mg,P	H,C,N,O,Na,Mg,P,Cl
MTR	6	2	46–62	1,2	H,C,N,O	H,C,N,O,Na,P,Cl
SAR	3	1,2,3	30–44	0,1	H,C,N,O,Mg,P	H,C,N,O,Na,Mg,P,Cl
RNase A	1	3	117	1	H,C,N,O,P	H,C,N,O,Na,P,S,Cl

a Each system setup is described in depth in the “System-specific preparation” section of the Supporting Information. The details of each setup include the number of mechanistic pathways N­(paths) probed, the number of reaction coordinates N­(RC) defining the pathways, the number of atoms in the QM region N­(QM), the net charge of the QM region Q­(QM), the elements in the QM region Z­(QM), and the elements in the MM region Z­(MM).

### Data Collection and Training Procedure

Foundation models are often trained to very large *ab initio* databases of small, gas-phase molecules. The conformations are generated from a number of sources, such as geometry optimization, torsional scans, normal-mode displacements, or by extracting molecular configurations from condensed-phase simulations for gas-phase evaluation. In other words, the *ab initio* databases used to train MLP or ΔMLP models have been used to develop models that seek to reproduce QM–QM interactions; they are not intended to modify QM–MM interactions in a QM/MM framework. In contrast, the models trained in the present work correct both QM–QM and QM–MM interactions. The parametrizations described below calculate QM/MM energies and forces of condensed-phase simulations with full periodic boundary conditions rather than extracting small-molecule fragments. The phosphate chemistry being targeted involves highly charged compounds that are often not electronically stable in the gas phase.

The training procedure collects data from inexpensive DFTB3/3ob QM/MM umbrella sampling, recalculates the condensed-phase energies with an *ab initio* QM/MM potential, and trains a ΔMLP correction to reproduce the differences in their energies and forces. The procedure starts by performing standard umbrella sampling with DFTB3/3ob QM/MM to locate a minimum free energy path (MFEP) using the surface-accelerated string method (SASM).^142^ The string method is iterated until the path is converged, and production sampling is performed along the MFEP. 32 umbrella windows were used for path-finding and production simulations. Each (low-level) DFTB3 QM/MM production simulation was run for 25 ps, and 500 samples from each simulation were saved. The energies and forces of the 16000 samples were recalculated with a (high-level) PBE0/6–31G* QM/MM potential. The high-level calculations are periodic condensed-phase evaluations utilizing the ambient potential composite Ewald method.^143^ Although the low- and high-level energies and forces explicitly include long-range interactions and periodicity, the ΔMLP is a short-range nonelectrostatic correction, so it can only correct the interactions of the QM region and its interaction with the 6 Å layer of neighboring MM atoms. The dpamber Python package^144^ was used to extract the coordinates, difference forces, and difference energies from the sander output files and package the results into the format used by the DP-GEN software.^145^

The network parameters were optimized with the DP-GEN “simplify″ functionality.^145^ We refer to this approach generally as pool-based active learning (P-AL). This approach is intended to “simplify″ a large data set by training multiple stochastically initiated models, referred to as a committee, in parallel to estimate uncertainty via a model deviation metric (ε_*F*,*i*_) applied to the remaining data pool:

2 $$\varepsilon_{F,i}=\sqrt{\left\langle ∥F_{i}\left(x;\Theta_{k}\right)-\left\langle F_{i}\left(x;\Theta_{k}\right)\right\rangle {∥}^{2}\right\rangle }$$

where *F*_*i*_ is the atomic force for a given data frame, *x*, and Θ is the set of parameters defining the model *k*. The DP-GEN “simplify” procedure consists of the following steps.

1. Train *k* = 4 models on an initial subset of data, producing a committee of four models (Θ_4_).
2. Compute model deviation for all remaining samples in the data set.
3. Remove samples for which the models sufficiently agree (ε_*F*,*i*_ < 0.08 eV/Å) and extreme outliers (ε_*F*,*i*_ > 2.0 eV/Å), retaining a “simplified″ pool of candidates.
4. Repeat until all samples have been trained on or removed from the pool.

In this way, training is focused on areas of greater need while reducing redundancy. In the first P-AL iteration, 20,000 samples were considered using 300,000 steps of Adam stochastic optimization^146^ with an initial learning rate of 0.001 that decayed exponentially to 5e-8. 10,000 new structures were subsequently added to the data set at each iteration with the number of training steps reduced to 100,000. The loss function contains contributions from energies and forces, and the prefactor associated with each property has a dependence on the learning rate. The initial and final prefactors of the energy term are 0.02 eV^–2^ and 1.0 eV^–2^, respectively, and the initial and final prefactors of the force terms are 1000 eV^–2^ Å^2^ and 1.0 eV^–2^ Å^2^. A detailed description of the loss function can be found in ref 129.

A system-specific refinement for the VSr reaction was performed with the DP-GEN “run″ procedure, an approach we refer to as on-the-fly active learning (O-AL) to distinguish from P-AL. In this case, training and hyperparameters were the same as for the P-AL approach. Structures were generated “on-the-fly″ from 2 ps QM/MM simulations of 32 umbrella windows along the ΔMACE minimum free energy path, writing model deviation statistics every 20 steps.

### Model Validation

#### Energy and Atomic Force Errors

Model validation on the training and test sets was performed by comparing the absolute energy errors and atomic force errors for the QM atoms and nearby MM atoms within 6 Å of the QM region. The test sets include umbrella sampling that was not included in the training (that is, umbrella windows located between trained umbrella centers), high energy paths (the initial, linear interpolation path guess rather than the MFEP), and modifications to the MM environment and/or QM region. The “nontrained window” sampling is generated with DFTB3/3ob QM/MM simulations performed along the MFEP, and each simulation provides 500 samples.

The remaining test data (the high energy paths, QM, and MM modifications) were collected by equilibrating the system with DFTB3/3ob QM/MM for 5 ps upon modification, and saving 20 samples from an additional 1 ps of sampling on the linearly interpolated path to collect forces. The purpose of the test data is to quantify the model’s ability to perform under circumstances not included directly in the training; therefore, the inclusion of converged sampling along the MFEP was not priority.

#### Potential of Mean Force Profiles from Fixed-Path Umbrella Integration

Another approach to validate the trained models is to compare reaction free energy profiles to reference *ab initio* QM/MM sampling. For this purpose, we analyze the sampling using the umbrella integration method.^147^ The QM/MM path is sampled from *N* simulations biased by a harmonic potential, *U*_bias,*i*_, where *i* indexes the bias centered at reaction coordinate **ξ**_*i*_.

3 $$U_{\mathrm{bias},i}\left(R\right)=\frac{1}{2}{\left(\xi (R)-\xi_{i}\right)}^{T}\cdot K\cdot \left(\xi \left(R\right)-\xi_{i}\right)$$

$\xi \left(R\right)=\xi_{1},\xi_{2},\cdots ,\xi_{N_{\mathrm{rc}}}$ is the array of *N*_rc_ reaction coordinates calculated from the atomic positions, **R**. **K** is an *N*_rc_ × *N*_rc_ diagonal matrix of force constants. The simulations were performed with umbrella force constants of 500 kcal/mol·Å^2^ for 400 fs along the MFEP obtained from the ΔMACE potential. Both sets of simulations were initiated from the same initial structures and velocity seeds to isolate the difference in the free energy profiles to differences in the potentials.

The biased samples from simulation *i* produce a 1 × *N*_rc_ array of reaction coordinate mean values ⟨**ξ**⟩_*i*_ and a *N*_rc_ × *N*_rc_ covariance matrix **C**_*i*_. The biased sampling of state *i* is used to estimate the gradient of the unbiased free energy in the vicinity of the observed samples **ξ** ≈ ⟨ **ξ**⟩_*i*_.

4 $${\frac{\partial A(\xi )}{\partial \xi_{d}}|}_{\xi \approx \langle \xi \rangle_{i}}=\overset{N_{\mathrm{rc}}}{\underset{d^{\prime }=1}{\sum }}C_{i,dd^{\prime }}^{-1}\frac{1}{\beta }\left(\xi_{d^{\prime }}-\langle \xi_{d^{\prime }}\rangle_{i}\right)-K_{dd^{\prime }}\left(\xi_{d^{\prime }}-\xi_{id^{\prime }}\right)$$

Short simulations lead to large uncertainties in the calculated covariance matrix; therefore, we approximate the covariance matrix from the biasing potential’s analytical Boltzmann distribution. This approximation assumes the force constant is sufficiently large that the unbiased potential energy is well-modeled by a plane in the localized vicinity of the samples.

5 $$C_{i,dd^{\prime }}\approx \delta_{dd^{\prime }}/\left(\beta K_{dd}\right)$$

The sequence of *N* biasing potentials is chosen to discretize a parametric curve **ξ**(*t*), where *t* ∈ [0,1] is a progress variable, such that ξ_*i*_ corresponds to a value of *t*_*i*_. The free energy profile is the cumulative integral along the path.

6 $$A\left(t\right)=\int_{t^{\prime }=0}^{t}\overset{N_{\mathrm{rc}}}{\underset{d=1}{\sum }}\frac{\partial A\left(\xi \right)}{\partial \xi_{d}\left(t^{\prime }\right)}\frac{d\xi_{d}\left(t^{\prime }\right)}{dt^{\prime }}dt^{\prime }$$

We solve this integral by calculating the integrand at *t*_*i*_ = *i*/(*N*–1) uniform positions corresponding to the *N* simulations. A cubic spline is fit to the discrete values and analytically integrated to produce a cubic spline of *A*(*t*). We plot the PMF by interpolating the *A*(*t*) spline at 200 points.

### Computational Details

#### System Preparation

A major goal of this work is to leverage and build upon existing data to generate a data set of NAzyme reactions; therefore, we leverage many systems built and rigorously equilibrated in MM from previous studies. In addition, we incorporated new data in anticipation of systems of interest for further investigation outside the scope of this work. A description of system-specific details including QM regions and reaction coordinate definitions can be found in Supporting Information along with QM region depictions in Figures S1–S11. In general, each system has been solvated in TIP4P-Ew water molecules,^148^ neutralizing Na^+^, and additional NaCl was added to yield a physiological concentration of 140 mM. The Joung and Cheatham monovalent ion parameters^149^ and the Li and Merz 12–6–4 divalent ion parameters^150^ were used. Panteva corrections^151^ were applied for systems containing Mg^2+^ ions to balance interactions between the solvent and nucleic acids. All RNA components were described with the OL3 force field,^152,153^ DNA with OL15,^154^ and proteins with ff14SB.^155^ Charges for nonstandard residues were determined using the restrained electrostatic potential (RESP) charge fitting method,^156,157^ where the potential was calculated at HF/6–31G*.

#### QM/MM Simulations

All QM/MM simulations and single-point calculations were performed with sander in AMBER24.^158^ Temperatures were regulated with a Langevin thermostat^159^ with a collision frequency of 5 ps^–1^, and pressures were controlled with the Monte Carlo barostat.^160^ The SHAKE algorithm^161^ was used to fix MM bonds involving hydrogen; however, all QM bonds remained unconstrained.^162–164^ The semiempirical-based QM/MM and QM/MM+ΔMLP simulations calculated long-range electrostatics with an Ewald method that uses Mulliken charges,^165,166^ and *ab initio* QM/MM calculations used the ambient potential composite Ewald method.^143^ Covalent bonds at the QM/MM boundary were treated with the hydrogen link-atom approach.^167,168^ Benchmark free energy profiles were generated from 25 ps of sampling with DFTB3/3ob,^74,75,131^ DFTB3/3ob:nhmod,^75^ DFTB3/3ob:ophyd,^169^ AM1/d-phoT,^170^ GFN2-xTB,^171^ QDπ,^80^ and MACE-OFF23­(M)^111^ potentials. The MACE-OFF model was converted for use with the AMBER DeePMD-kit interface using the deepmd-gnn mace-off convert functionality in the DeePMD-GNN plugin, and the cutoff radius was set to zero given that the model was not trained on QM/MM interactions. For multidimensional reaction sampling, the reaction string was converged using the surface accelerated string method^142^ included in the FE-ToolKit package.^172^ Simulations were performed for 1 ps per umbrella window per string iteration in the canonical ensemble at 298 K using a 1 fs time step, outputting the reaction coordinate every 20 steps. The string was updated at each iteration by performing a maximum of 300 “synthetic iterations″ to optimize a path with 50 control points and 400 spline points. Unless indicated otherwise, the free energy surface was approximated by a weighted average of nearby bin values computed from the multistate Bennett acceptance ratio (MBAR) method^173^ using fourth-order B-splines. The only difference between DFTB3 and QM/MM+ΔMLP simulations was the application of the ΔMLP to the QM region and QM/MM interactions within a 6 Å cutoff radius. For comparison, additional analysis was performed with the Weighted Histogram Analysis Method (WHAM)^174,175^ as implemented in ref 176.

## Results and Discussion

### Motivation for Training a QM/MM+ΔMLP Specifically for NAzyme Catalysis

We first motivate the approach taken in this work by demonstrating how existing popular methods perform for representative NAzyme reactions. In previous studies, we have established that PBE0/6–31G* is generally a sufficiently accurate QM method to reliably predict experimentally relevant properties, including free energy barriers^57,58,110,177,178^ and kinetic isotope effects.^59^ While dispersion effects in the density functional model are valuable, including dispersion corrections at the PBE0-D3/6–31G* level did not yield meaningful improvements in reaction barriers, and hence they are not included in the current work. It is likely that their inclusion in more computationally costly density functionals such as ωB97M-D3­(BJ)/def2-TZVPPD^179–184^ might lead to improvements, which we leave to future work.

In Figure 2, we compare free energy profiles at the DFTB3, AM1/d, GFN2-xTB, QDπ, MACE-OFF­(M), and PBE0/6–31G* levels of theory for three previously published reactions for which expensive *ab initio* sampling was performed: deprotonation of the O2′ nucleophile in the pistol ribozyme (PSr) by guanine (G) and 2-aminopurine (2AP);^177^ the two-step proton and methyl transfer reaction of the methyltransferase ribozyme (MTR1);^30,178^ and the phosphoryl transfer reaction of the Varkud Satellite ribozyme (VSr) departing from a deprotonated nucleophile.^58^ Free energy profile details are provided in Table S1, and schematics of these reactions are provided in Figure S12 of the Supporting Information. The QDπ and MACE-OFF models were excluded from the VSr comparison because they do not include Mg^2+^, a limitation shared by other universal models trained primarily for organic systems.

**Figure 2:**
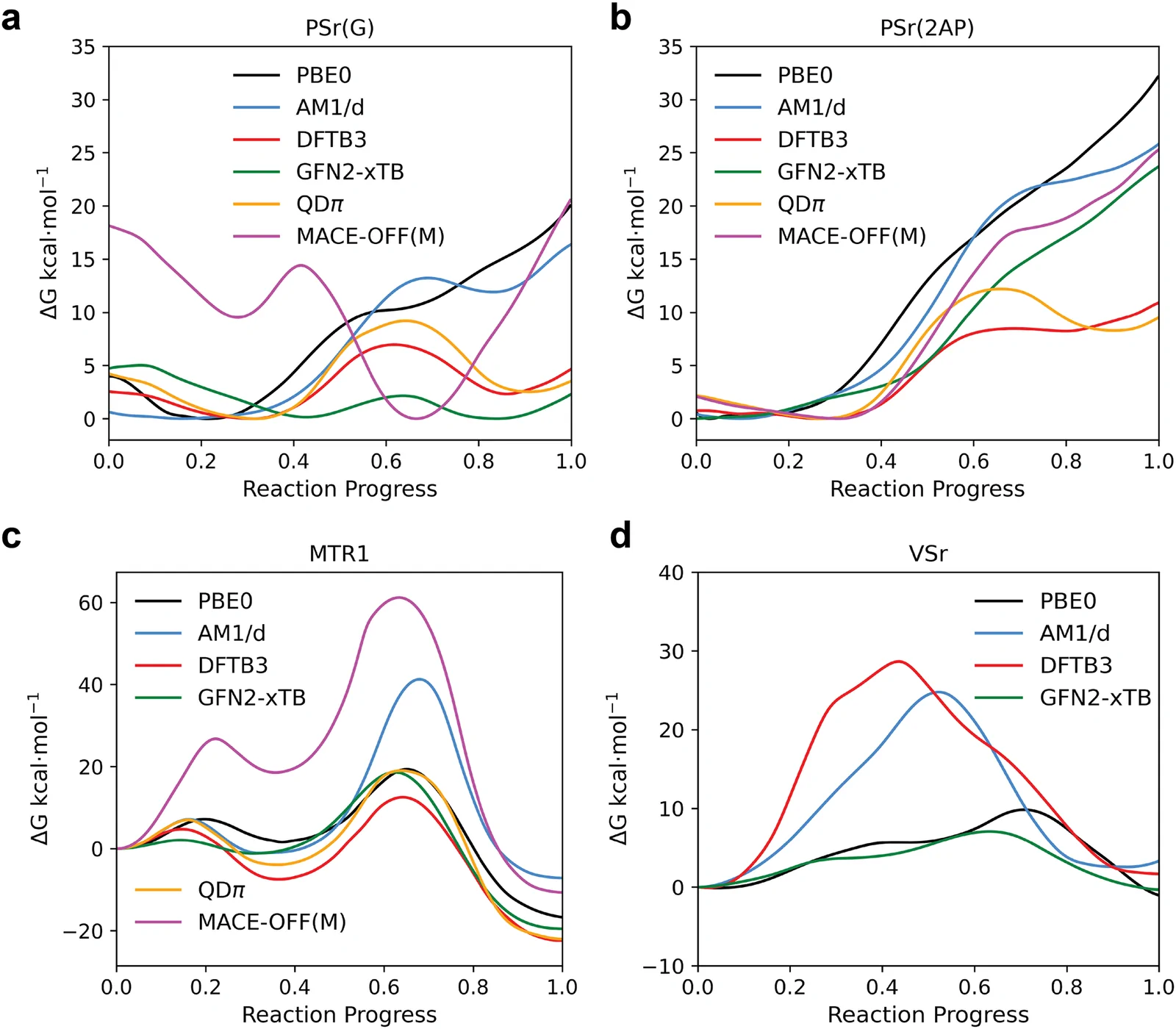
Free energy profiles obtained from QM/MM simulations at semiempirical (DFTB3, AM1/d, GFN2-xTB), QDπ, MACE-OFF23­(M), and PBE0/6–31G* levels of theory. Deprotonation of the nucleophile O2′ by general base (a) G and (b) 2AP in PSr. (c) Two-step proton and methyl transfer reaction of MTR1. (d) Phosphoryl transfer and leaving group protonation in VSr.

As shown in Figure 2, no single method reproduces the PBE0 trends across all three reactions, underscoring the need for a more accurate model that incorporates interactions with the MM environment. In PSr, the G versus 2AP deprotonation free energy difference should be approximately 7.5 kcal·mol^–1^ in agreement with PBE0,^177^ but no semiempirical method reproduces this; GFN2-xTB even predicts deprotonation of O2′ by G to be favorable, inconsistent with the p*K*_a_ values of G (9.4) and O2′ (12.5).^185^ In MTR1, AM1/d (the closest method to PBE0 for PSr) dramatically overestimates the barrier, and all methods except GFN2-xTB overestimate the intermediate stability. For VSr phosphoryl transfer, the AM1/d and DFTB3 barriers are large enough to render the ribozyme catalytically inactive; GFN2-xTB gives a more reasonable barrier, but extended sampling of the full three-dimensional surface (including nucleophile activation, below) exhibits a spuriously favorable deprotonation step. In Figure S13, we provide additional free energy profiles for each reaction at the DFTB3/3ob:nhmod^75^ and DFTB3/3ob:ophyd^169^ levels of theory to benchmark how some existing semiempirical specific reaction parametrizations perform relative to PBE0 for proton transfers involving sp^3^-hybridized nitrogen and phosphoryl transfer. While DFTB3/3ob:ophyd improves the VSr barrier, neither method fully aligns with PBE0. The QDπ model gives reasonable barriers where applicable, but the nature of the training data is not amenable for many NAzyme reactions. Together, these results show that solution-phase training data and explicit QM/MM interactions at the *ab initio* level are essential for experimentally relevant predictions in NAzyme active sites.

### Selection of the Base QM Method

Before training, it was essential to determine the reference level of theory to be corrected by the ΔMLP. The ideal reference method is one whose forces already agree most closely with PBE0, minimizing the magnitude of the Δ that must be learned. To this end, we evaluated forces at the DFTB3, AM1/d, and GFN2-xTB levels relative to PBE0/6–31G* for four reactions spanning diverse chemistry and system size: 1) nonenzymatic 2′-*O*-transphosphorylation catalyzed by Zn^2+^, where the metal-coordinated nonbridging phosphoryl oxygen (NBO) is substituted by sulfur; 2) 4-aminomethyl-benzyl (alkyl) transfer catalyzed by MTR1; 3) 2′-*O*-transphosphorylation catalyzed by HDVr; and 4) 3′-*O*-transphosphorylation catalyzed by THr. The active sites of these systems are depicted in Figure 1b, and schematics of the reactions are provided in Figure S14 of the Supporting Information. Because these reactions occur in at least two dimensions, simulations were performed along a path linearly interpolating from reactant to product, representing an initial guess of the minimum free energy path. This path is likely to be high in energy, but it is essential that the selected reference method perform well across the diverse structures that may be encountered during path finding. A total of 40 energy and force evaluations were collected per umbrella window, yielding 1280 samples per system. The resulting force errors relative to PBE0/6–31G* are reported in Table 2, with force comparisons shown in Figure S15 of the Supporting Information.

**Table 2:** Error in the Magnitude of Forces on Atoms within *r*_cut_ of the QM Region Obtained with Trial Semiempirical Methods Compared to PBE0/6–31G*

	Error(|*F*|) kcal·mol^–1^ Å^–1^					
	AM1/d		DFTB3		GFN2-xTB	
Model	RMSE	MAE	RMSE	MAE	RMSE	MAE
NE	5.08	1.40	4.03	0.96	3.84	1.32
MTR	8.79	2.06	4.20	1.09	2.89	1.00
HDVr	6.97	1.80	4.13	1.07	^a^3.43	^a^1.15
THr	6.50	1.72	3.94	0.95	4.25	1.27

a Umbrella windows with self-consistent field convergence failures were excluded from the analysis (160/1280 samples). Each reaction pathway corresponds to a straight line from reactant to product, and systems correspond to those in Figure 1b.

GFN2-xTB simulations of HDVr encountered self-consistent field convergence failures in the reactant and product regions, where negative charge is concentrated on the nucleophile or leaving group. More broadly, we observe a systematic contraction of the reaction paths obtained with GFN2-xTB, reflecting an avoidance of highly negatively charged states. Although GFN2-xTB yields the lowest force errors relative to PBE0 for converged windows, its inability to adequately sample regions of concentrated negative charge precludes its use as a reference method, as these are precisely the regions that must be well represented in the training data. DFTB3, which yields the next lowest force errors across all four systems, is therefore selected as the reference level of theory to be corrected by the ΔNAzyme models.

### Pool-Based Active Learning Procedure Yields Accurate Forces and Energies for Training Set

We trained the ΔNAzyme models with the pool-based active learning (P-AL) procedure of DP-GEN outlined in Figure 3a (see Methods), in which the NAzyme data set is the initial pool and a committee of four models is used to identify accurate samples for removal at each iteration, focusing training on regions of greater uncertainty while reducing redundancy. The DFTB3-level free energy profiles generated in the process of curating the NAzyme data set are provided in Figures S16–S20 of the Supporting Information.

**Figure 3:**
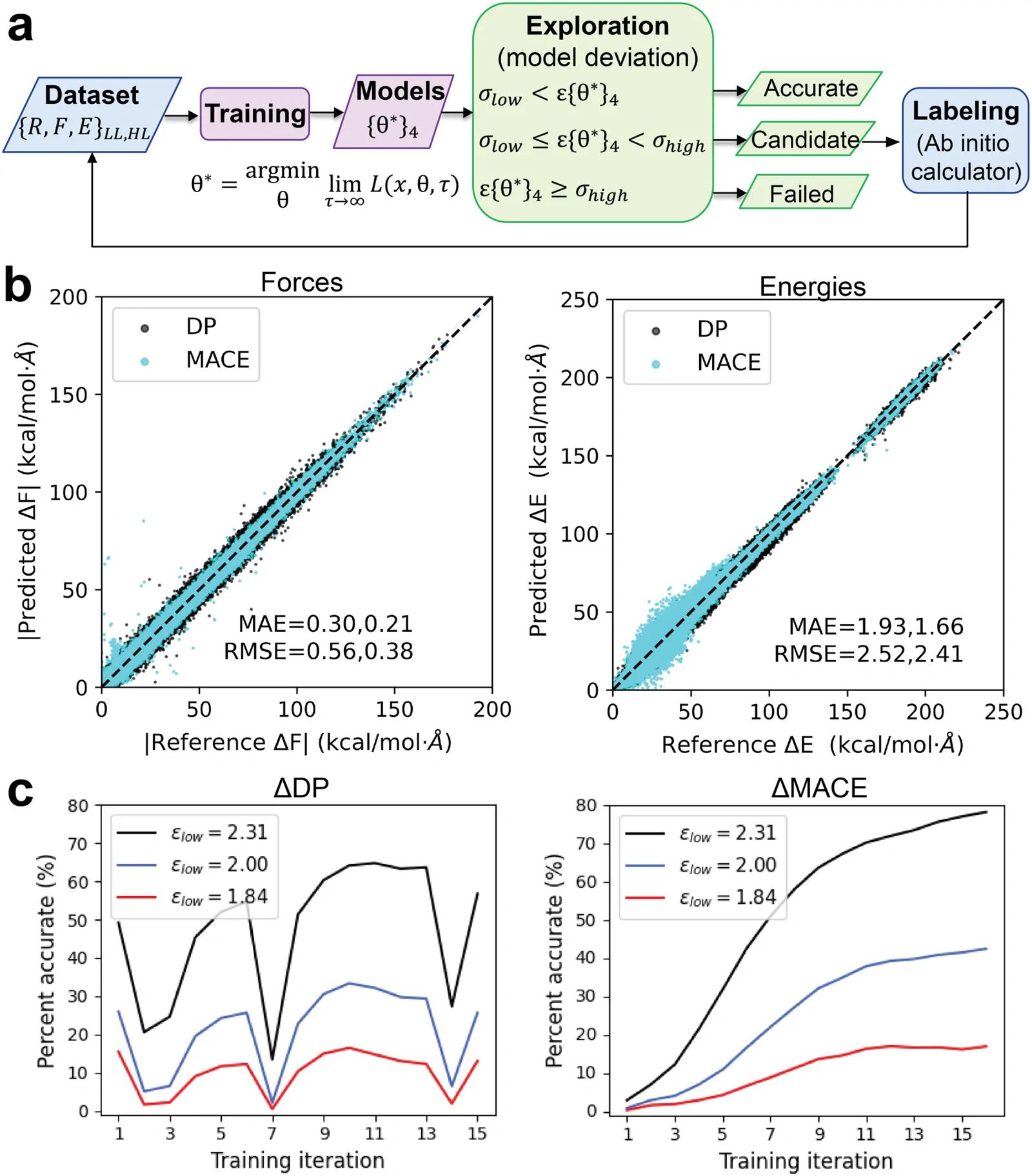
Training procedure and associated errors for ΔDP and ΔMACE models. (a) Schematic representation summarizing the pool-based active learning approach used to train the models in this work. Inputs and outputs are depicted with trapezoids and processes with rectangles. (b) Training errors in predicted forces and energies by the ΔDP (black) and ΔMACE (cyan) models on the large NAzyme data set. RMSEs and MAEs are reported as ΔDP,ΔMACE in kcal·mol^–1^ Å^–1^ for forces and kcal·mol^–1^ for energies. (c) Percentage of samples deemed accurate based on model deviation in the remaining pool as a function of pooled-based active learning iteration. ε_low_ was set to 1.84 kcal·mol^–1^ Å^–1^ during training (red), and we provide analysis with cutoffs of 2.00 (blue) and 2.31 (black) for context.

We trained ΔNAzyme models using the DP and MACE architectures on the same NAzyme data set with the P-AL procedure to enable direct comparison. The DP model is rotationally invariant, whereas equivariant models such as MACE preserve directional information while enforcing rotational symmetry, allowing more efficient representation of complex atomic interactions and often resulting in improved accuracy and generalization.^103,120,126,186^ The root-mean-square error (RMSE) and mean absolute error (MAE) in the magnitude of force and energy differences predicted by the model relative to the actual DFTB3-to-PBE0 correction are reported in Table 3 for both the small (S) and large (L) training sets with the ΔDP and ΔMACE models, with system-specific parity plots provided in Figures S21 and S22 of the Supporting Information.

**Table 3:** RMSE and MAE in the Magnitude of Force and Energy Differences Predicted by the Model Compared to the Actual Difference between DFTB3 and PBE0 for Systems in the Training Set^a^

Model	ΔDP	ΔMACE	ΔDP	ΔMACE	ΔMACE-L
Data set	S	S	L	L	L
	|*F*| RMSE(MAE) (kcal·mol^–1^ Å^–1^)				
NE	0.55(0.30)	0.38(0.21)	0.56(0.30)	0.33(0.19)	0.36(0.20)
HDVr	0.55(0.30)	0.39(0.22)	0.55(0.29)	0.35(0.20)	0.38(0.22)
HHr	0.58(0.33)	0.43(0.25)	0.58(0.33)	0.40(0.23)	0.43(0.26)
PSr	0.53(0.30)	0.39(0.22)	0.53(0.29)	0.35(0.20)	0.39(0.23)
VSr	0.58(0.33)	0.44(0.25)	0.56(0.32)	0.40(0.23)	0.45(0.27)
TWr	0.80(0.45)	0.57(0.33)	0.81(0.44)	0.61(0.36)	0.66(0.37)
8–17dz	0.67(0.36)	0.52(0.29)	0.67(0.35)	0.49(0.27)	0.60(0.33)
RNase A	0.68(0.35)	0.51(0.27)	0.63(0.33)	0.46(0.25)	0.51(0.27)
THr	0.54(0.29)	0.40(0.22)	0.54(0.29)	0.36(0.20)	0.39(0.22)
MTR	0.57(0.34)	0.55(0.33)	0.57(0.33)	0.47(0.29)	0.60(0.36)
SAR	0.53(0.29)	0.43(0.24)	0.54(0.29)	0.41(0.23)	0.46(0.26)
	*E* RMSE(MAE) (kcal·mol^–1^)				
NE	2.88(2.26)	2.64(1.96)	2.57(2.10)	1.79(1.40)	2.16(1.73)
HDVr	3.49(2.82)	2.53(2.16)	5.16(4.53)	1.53(1.22)	1.98(1.58)
HHr	3.16(2.51)	3.31(2.80)	3.02(2.41)	2.06(1.63)	2.95(2.40)
PSr	1.67(1.33)	1.45(1.15)	2.59(1.99)	1.65(1.37)	2.10(1.68)
VSr	3.42(2.85)	4.71(4.17)	3.05(2.36)	2.27(1.77)	2.94(2.33)
TWr	3.83(3.03)	2.47(2.00)	3.52(2.86)	3.41(2.69)	3.45(2.76)
8–17dz	4.75(3.80)	3.97(3.17)	4.14(3.41)	3.78(3.07)	4.67(3.67)
RNase A	4.04(3.22)	3.61(2.89)	4.51(3.65)	4.80(4.12)	4.47(3.63)
THr	2.83(2.23)	2.09(1.64)	2.52(1.98)	2.66(2.01)	2.81(2.13)
MTR	3.45(2.67)	6.14(4.77)	3.26(2.56)	5.14(3.88)	6.37(4.91)
SAR	4.49(3.48)	3.77(3.06)	2.87(2.03)	2.45(1.97)	2.61(2.06)

a Statistics are given as RMSE­(MAE) of either the magnitude of the force or energy correction. The data set for training is either small (S) or large (L). ΔMACE indicates a ΔMACE with 128 channels, while ΔMACE-L is a larger model with 256 channels.

For the ΔDP model, training on the large versus small data set does not produce a consistent improvement in accuracy across systems, whereas the ΔMACE model generally achieves lower errors on the larger data set. For both data sets, ΔMACE consistently outperforms ΔDP. Given this advantage, we examined whether accuracy could be further improved by increasing the model size. The ΔMACE-L model uses the same hyperparameters as ΔMACE, except the number of scalar and vector channels is doubled from 128 to 256. This increase in model size did not yield a systematic improvement in training set accuracy; errors for several systems (i.e., MTR1 and 8–17dz) were comparable to or slightly higher than those of the default ΔMACE model.

A further distinction between the ΔDP and ΔMACE models is observed in the trend of model deviation over successive P-AL iterations, shown in Figure 3c, which reports the percentage of the remaining pool deemed accurate by the model deviation criterion at each iteration. This quantity should increase as the model improves. The ΔDP model exhibits sharp drops in the fraction of the pool deemed accurate, particularly in iterations 7 and 14, whereas the ΔMACE model increases smoothly, suggesting that ΔDP is less transferable to previously unseen environments encountered during the active learning. The training subset is chosen randomly from the shuffled remaining pool, so if a system happens to be poorly represented in a subset, the ΔDP model may fail to extrapolate, which makes this pool-based approach less effective for this type of model. The ΔMACE model represents a cumulative improvement over training iterations with the desired data-pruning effect, whereas the ΔDP model is more reflective of its most recent training subset. This is reflected in the number of samples pruned from the data set, which is detailed in Table S2. Overall, ΔMACE achieves lower errors than ΔDP across training set systems. Next, we further probe the transferability of the models beyond the training sets to experimentally relevant test sets.

### ΔMACE Model Offers Greater Transferability Compared to ΔDP or ΔSRPs on Experimentally Relevant Test Sets

Mechanistic studies that guide design routinely test scenarios such as mutagenesis or changes to the ionic environment, so a foundational model must be reasonably transferable to such cases. Returning to the four systems used to benchmark the semiempirical methods (Figure 1b), we define four test set scenarios per system: nontrained umbrella windows that fall on the MFEP between trained windows, high energy reaction pathways, QM region modifications, and MM region modifications within 6 Å of the QM region.

The QM and MM modifications were chosen to represent scenarios that commonly arise when computing experimental observables. For the nonenzymatic reaction, the QM modification thio-substitutes both NBOs, a combination absent from the training set, and the MM modification adds a Na^+^ ion at a solvent separation from the leaving group. For MTR1, the QM modification is a 7-deaza substitution to the target adenine (A63) that raises the nucleophile p*K*_a_, and the MM modification mutates the adenine stacked on A63 (A40) to guanine. For HDVr, the QM modification is a ΨisoC substitution to the general acid C75 that lowers its p*K*_a_, and the MM modification swaps a structural Mg^2+^ for Na^+^. For the THr ligation reaction, the QM modification mutates the leaving group guanine 2′-OH, which directly coordinates a Mg^2+^, to H; the MM modification changes a nearby Na^+^ to Mg^2+^, producing more Mg^2+^ ions than were present in any training environment. For each system, the “high energy path″ is the initial, linearly interpolated path from reactant to product, distinct from the MFEPs used in training. Transferability to paths other than the DFTB3 MFEP is important in practice because the high-level MFEP often differs from the low level.

Table 4 uses the test sets described in the previous paragraph to compare the force errors between the uncorrected DFTB3 model and the ΔMLP-corrected models, with accompanying parity plots provided in Figures S23–S26 of the Supporting Information. All of the ΔMLP models extrapolate well to the nontrained windows, even for the small data set. They also extrapolate well to the nontrained, high energy paths. In general, QM modifications led to higher errors than MM modifications, but none of the models performed worse than DFTB3. The ΔMACE model trained on the large data set displayed the lowest errors across the test sets, making it most amenable to a streamlined computational enzymology approach.

**Table 4:** RMSE and MAE in the Magnitude of Forces on Atoms within *r*_cut_ of the QM Region Predicted by the Reference Potential Compared to the Target Potential (PBE0/6–31G*) for Test Sets Probing Transferability^a^

	RMSE(MAE) (|F|) (kcal·mol^–1^ Å^–1^)							
Potential	DFTB3	ΔDP	ΔMACE	ΔDP	ΔMACE	ΔMACE-L	ΔMACE (SRP)	ΔMACE (R-SRP)
Data set	N/A	S	S	L	L	L	L	L
	Trained windows							
NE	5.22(1.17)	0.38(0.17)	0.28(0.12)	0.43(0.17)	0.25(0.11)	0.25(0.11)	0.21(0.10)	0.14(0.07)
MTR	3.88(1.06)	0.47(0.21)	0.42(0.22)	0.46(0.21)	0.37(0.18)	0.45(0.23)	0.25(0.12)	0.15(0.25)
HDVr	4.83(1.21)	0.65(0.23)	0.51(0.16)	0.48(0.20)	0.29(0.14)	0.32(0.15)	0.43(0.14)	0.27(0.10)
THr	3.90(0.94)	0.40(0.16)	0.33(0.14)	0.41(0.16)	0.29(0.12)	0.29(0.13)	0.18(0.09)	0.13(0.06)
	Nontrained windows							
NE	5.21(1.12)	0.41(0.17)	0.28(0.12)	0.41(0.17)	0.23(0.10)	0.26(0.11)	0.20(0.09)	0.14(0.07)
MTR	3.96(0.98)	0.45(0.21)	0.42(0.21)	0.46(0.21)	0.37(0.19)	0.44(0.23)	0.25(0.12)	0.15(0.25)
HDVr	4.62(1.20)	0.64(0.22)	0.50(0.16)	0.48(0.20)	0.30(0.14)	0.33(0.15)	0.40(0.14)	0.28(0.10)
THr	3.95(0.95)	0.41(0.16)	0.32(0.14)	0.41(0.16)	0.28(0.12)	0.28(0.13)	0.17(0.08)	0.13(0.07)
	High energy paths							
NE	4.03(0.96)	0.46(0.18)	0.33(0.13)	0.45(0.18)	0.26(0.11)	0.29(0.12)	0.26(0.10)	0.17(0.08)
MTR	4.20(1.09)	0.50(0.22)	0.46(0.22)	0.47(0.22)	0.39(0.19)	0.47(0.23)	0.27(0.13)	0.16(0.09)
HDVr	4.13(1.07)	0.51(0.19)	0.36(0.15)	0.46(0.18)	0.28(0.13)	0.31(0.14)	0.33(0.12)	0.22(0.09)
THr	3.94(0.95)	0.46(0.17)	0.35(0.14)	0.43(0.16)	0.30(0.12)	0.32(0.13)	0.25(0.09)	0.17(0.07)
	QM modifications							
NE	4.92(1.18)	0.68(0.24)	0.47(0.17)	0.63(0.23)	0.37(0.15)	0.39(0.15)	0.63(0.19)	0.42(0.15)
MTR	3.97(1.00)	0.53(0.23)	0.49(0.22)	0.52(0.23)	0.48(0.20)	0.47(0.22)	0.73(0.19)	0.64(0.16)
HDVr	4.01(1.05)	0.91(0.27)	0.83(0.24)	0.82(0.25)	0.83(0.22)	0.86(0.24)	1.15(0.23)	0.77(0.19)
THr	3.91(0.93)	0.61(0.20)	0.52(0.17)	1.87(0.28)	0.45(0.15)	0.43(0.15)	0.53(0.14)	0.32(0.10)
	MM modifications							
NE	4.04(0.94)	0.45(0.18)	0.33(0.13)	0.45(0.17)	0.28(0.12)	0.29(0.12)	0.30(0.11)	0.19(0.08)
MTR	4.00(1.06)	0.47(0.22)	0.44(0.22)	0.44(0.21)	0.37(0.19)	0.45(0.23)	0.25(0.12)	0.17(0.09)
HDVr	4.21(1.02)	0.61(0.21)	0.49(0.17)	0.57(0.20)	0.49(0.16)	0.46(0.16)	1.05(0.20)	0.42(0.12)
THr	4.14(1.01)	0.51(0.18)	0.42(0.15)	0.49(0.18)	0.36(0.14)	0.36(0.14)	0.29(0.11)	0.22(0.08)

a Statistics are given as RMSE­(MAE) of the absolute magnitude of the force to enable comparison with DFTB3. The data set for training is either small (S) or large (L). ΔMACE indicates a MACE model with 128 channels, while ΔMACE-L is a larger model with 256 channels. ΔMACE­(SRP) indicates a specific reaction potential trained from scratch with the MACE architecture. ΔMACE­(R-SRP) indicates a refined specific reaction potential where training was initiated from the foundational ΔMACE model.

While the foundational model can explore mechanistic scenarios and trends in place of a semiempirical method, the most accurate experimental observables may warrant a fine-tuned model. We therefore refine the ΔMACE model separately for each of the four reactions with the P-AL approach, yielding refined specific reaction parametrizations (R-SRPs), and compare them to SRPs trained from scratch on the same data, as one would do without a foundational model. At no additional cost to the user, the R-SRPs reduce training set errors while retaining higher transferability to the experimentally relevant test sets than the SRPs.

### ΔNAzyme Model Serves as a Departure Point for Active Learning

With the ΔNAzyme models benchmarked, we now turn to a case-study application of the RNA cleavage reaction by VSr. This system was chosen because it is the most complex reaction for which an *ab initio* free energy profile has been computed and validated against experiment.^58^ It is also a system for which only one reaction path was used in the data set, and the only “G+A″ reaction in the data set, posing a challenging test for the model compared to other more represented systems or active site architectures. We performed reaction path-finding in three dimensions including nucleophile deprotonation by the general base guanine (RC1), phosphoryl transfer (RC2), and leaving group protonation by the general acid adenine (RC3) using the foundational ΔDP and ΔMACE models as well as the trial semiempirical models (see Figure S27 of the Supporting Information for a schematic of the reaction). Rigorous reaction path-finding at the *ab initio* level is extremely expensive; therefore, we used an on-the-fly active learning procedure (O-AL) to refine the ΔMACE potential for the VSr reaction, referred to as ΔMACE-AL, to obtain a highly accurate, reference-level potential for comparison.

To begin, we compare the free energy profiles and MFEPs obtained with the ΔNAzyme models and ΔMACE-AL model, with results shown in Figure 4a and b and Table S3 of the Supporting Information. For reference, experimental estimates of the free energy barrier are ∼16–17 kcal·mol^–1^.^58^ The ΔMACE and ΔDP models both yield reasonable barriers (15.1 and 16.9, respectively), but the shapes of the profiles are slightly different. The ΔDP model overpredicts the deprotonation barrier and predicts the wrong position of the rate-determining transition state, while the ΔMACE model overpredicts the stability of the metastable state preceding the transition state. The ΔMACE model pathway closely aligns with the ΔMACE-AL pathway as shown in Figure 4b, which is in excellent agreement with the two-dimensional PBE0 result.^58^ Even if refinement were not performed for the ΔMACE model, the resulting pathway and PMF would provide a much more informative view of the reaction than any of the tested semiempirical methods. The accuracy in the free energy profiles is reflected in the force and energy corrections predicted for the training set, as shown in Figure 4c.

**Figure 4:**
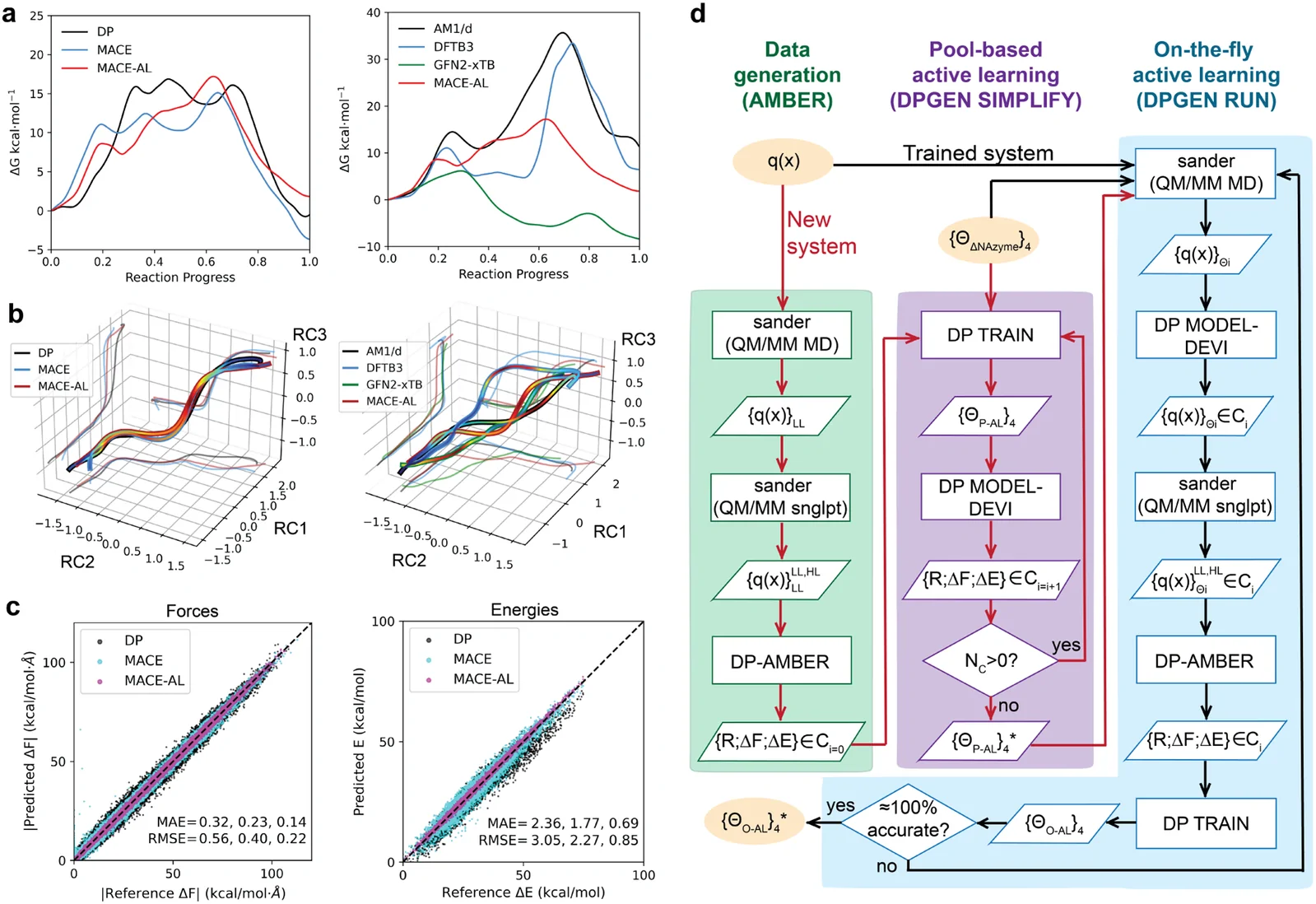
Overview of the procedure for model refinement and example application to the VSr reaction. (a) Free energy profiles at ΔMLP levels (left) and semiempirical levels (right) compared to the most accurate ΔMACE-AL model (red). (b) MFEPs at ΔMLP levels (left) and semiempirical levels (right) with colors corresponding to the free energy values and outline colors denoting the QM method. (c) Force and energy predictions made by the ΔMLP models compared to the reference values. Error statistics are given as ΔDP, ΔMACE, ΔMACE-AL. (d) Flowchart representation of two potential model refinement procedures including P-AL (red arrows) and O-AL (black arrows). Functions are represented by rectangles, inputs and outputs by trapezoids, decisions by diamonds, and terminal inputs and outputs by ovals.

The ΔNAzyme models are still a clear improvement over the three semiempirical approaches. Unlike the two-dimensional case shown in Figure 2, the GFN2-xTB method yields a 3-D MFEP where nucleophile deprotonation and phosphoryl transfer are more concerted, yielding a free energy barrier of 6.2 kcal·mol^–1^, well below experimental estimates.^58^ Meanwhile, the DFTB3 and AM1/d barriers of over 30 kcal·mol^–1^ would render the reaction experimentally undetectable. In addition, we performed path-finding and sampling with the DFTB3/3ob variants nhmod and ophyd, which yield more reasonable barriers of 22.5 and 21.3 kcal·mol^–1^, respectively, but still deviate considerably in the minimum free energy path. Additional DFTB3-level results are given in Figure S28. The ΔMACE-AL model, however, yields a barrier of 17.2 kcal·mol^–1^, in close agreement with experiment. One should also note that the MFEPs obtained with all three semiempirical models differ from that of ΔMACE-AL, which cautions against simply performing semiempirical path-finding and doing high-level simulations, reweighting, or optimizations directly on the low-level MFEP.

This example highlights an effective use case of the ΔNAzyme model: finding the MFEP with the foundational model in place of a semiempirical model, then performing O-AL or P-AL refinement to obtain a highly accurate, refined model for predicting experimental observables. While the range-corrected approach is valuable for the inclusion of QM/MM interactions, the explicit distinction between QM and MM atom types greatly increases the sampling challenge for training data generation. Therefore, to achieve the highest possible accuracy, it is desirable to train on an ensemble that closely aligns with the high level. Figure 4d demonstrates a general workflow for how one might use the foundational ΔNAzyme model. There are two paths one could follow depending on how much the new application differs from the training set. The more conservative approach for a system with new chemistry (indicated by red arrows) would be to perform semiempirical-level sampling to collect training data for P-AL. In our experience, using the ΔNAzyme model directly for a type of chemistry not present in the training set may result in bond distortions that can lead to SCF convergence issues. This will be discussed further in the next section. For systems within the training set, such as VSr, or with similar architectures and reactions, the refinement workflow could be accelerated by directly performing O-AL (black arrows), which uses the ΔNAzyme model to directly generate training data. In this approach, the ensembles generated increasingly mimic the high level as the model increases in accuracy, thus reducing the sampling burden. The specific components of the workflow outlined in Figure 4a are detailed in the Supporting Information.

When O-AL was applied for VSr, an initial data set of 3200 structures was generated on the ΔMACE MFEP, of which 3197 were deemed candidates. A subset of 500 structures from the candidate pool were drawn at random for labeling, which then served as data for the next training iteration. After this first O-AL iteration, a new 3200 structure set was generated, of which only 311 candidates remained. After 2 O-AL iterations, only 26 candidates remained, meaning 99.2% of the data set yielded model deviation below the accuracy threshold. This means that, in total, only 811 high-level evaluations were required to obtain the refined model.

### Transfer Learning from the ΔNAzyme Model Reduces the Number of *Ab Initio* Calculations Required for Future Applications

We next use the site-specific alkyltransferase ribozyme SAMURI^33^ to show how the foundational ΔNAzyme model accelerates studies of new chemistry. SAMURI binds S-adenosylmethionine (SAM), which transfers a methyl group to a target adenine (Figure S29). This system differs from the NAzyme data set in several respects: a positively charged sulfur center, a carboxylate-Mg^2+^ coordination in the QM region, and methylation at the adenine N3 position. As such, simulations that directly used the ΔMACE model eventually encountered a distorted bonding pattern in which a proton from the transferring methyl group bonded to another ligand carbon, showing that the model struggled to extrapolate to this new chemistry. We therefore took the more conservative P-AL approach for refinement.

We probed how using the ΔMACE model for refinement with transfer learning to generate a refined specific reaction parametrization (R-SRP) could reduce the number of computationally expensive single-point calculations needed to achieve high accuracy for a new application. We performed P-AL with data sets containing 500, 250, 100, 50, and 25 samples per umbrella window across 32 windows, and then used each model to predict force and energy differences on the full 500 samples per window data set to assess extrapolation. As shown in Table 5, the R-SRPs produce low errors even down to 25 samples per window, with a slight drop-off between 100 and 50 samples. For comparison, we trained SRPs without transfer learning on the same data sets, representing an approach one might take in the absence of a foundational model. These SRPs show a clear loss of accuracy as the sample count decreases, whereas the refined models remain comparable to, or better than, the 500 samples per window SRP even at 25 samples per window. The best balance of accuracy and cost is roughly 100 samples per window with transfer learning; if the P-AL model is intended only as a departure point for O-AL, 25 samples per window, or perhaps fewer, would suffice. In this way, the foundational model substantially reduces the need for expensive *ab initio* single-point calculations, even for new NAzyme reactions.

**Table 5:** Errors in Force and Energy Corrections Predicted by Specific Reaction Potentials with and without Transfer Learning for the SAMURI Ribozyme Reaction^a^

Number of samples		(kcal·mol^–1^ Å^–1^)		(kcal·mol^–1^)		%
Data set	Train	RMSE(|*F*|)	MAE(|*F*|)	RMSE(*E*)	MAE(*E*)	ε_*F*_ < 1.84
ΔMACE(R-SRP)						
500	500	0.19	0.12	0.76	0.62	99.99
500	250	0.19	0.12	0.69	0.56	99.81
500	100	0.19	0.12	0.70	0.57	99.72
500	50	0.23	0.15	1.02	0.85	99.74
500	25	0.24	0.15	0.94	0.75	99.34
250	250	0.19	0.12	0.64	0.51	99.90
100	100	0.18	0.12	0.74	0.61	99.95
50	50	0.22	0.14	0.64	0.50	100.00
25	25	0.21	0.14	0.53	0.42	100.00
ΔMACE(SRP)						
500	500	0.25	0.16	0.73	0.58	99.71
500	250	0.25	0.16	0.82	0.65	99.61
500	100	0.27	0.16	1.00	0.81	99.22
500	50	0.27	0.16	0.94	0.76	98.94
500	25	0.27	0.16	0.75	0.59	98.39
250	250	0.25	0.16	1.54	1.38	99.81
100	100	0.26	0.16	1.32	1.15	99.31
50	50	0.25	0.16	0.79	0.64	99.94
25	25	0.24	0.16	0.84	0.70	100.00

a Samples are given per window, with 32 windows considered for training. Test indicates the number of samples in the test set, and Train indicates the number of samples in the training set. All models use the MACE architecture. SRP indicates a specific reaction potential trained from scratch. R-SRP indicates a refined specific reaction potential where training was initiated from the foundational ΔMACE model. ε_*F*_ is the model deviation defined in eq 2.

### Potentials of Mean Force Validated Using 1-D Umbrella Integration

Finally, we validate the foundational ΔNAzyme model by comparing the ΔMACE free energy profiles to the target PBE0 level. We focus specifically on the ΔMACE model with default hyperparameters trained on the large data set that exhibited the highest accuracy across the training and test sets. We return to the PSr, MTR1, and VSr reactions for which PBE0 sampling has previously been performed,^58,177,178^ and sample the VSr reaction in 3 dimensions, which was not tractable in the original work. Direct *ab initio* sampling is intractable on the time scales accessible to ΔMLPs and is not an ideal validation approach for future applications due to computational expense. One option is to reweight the ΔMLP ensemble to the *ab initio* level using multireference generalized weighted thermodynamic perturbation (gwTP);^187^ this is effective for small systems, but its accuracy depends on the phase-space overlap between the ΔMLP and target total potentials, which decreases with system size. Because training weights forces above energies, however, the model reproduces the forces that drive reaction coordinate sampling, and hence the resulting free energy profile, more faithfully than the total potential energy on which gwTP reweighting depends, making umbrella integration the more reliable route at larger system sizes.

We find the MFEP rigorously at the ΔMACE level using umbrella force constants of 100 kcal·mol^–1^ Å^–2^, and then perform short simulations of 400 fs at the ΔMACE and PBE0 levels with 500 kcal·mol^–1^ Å^–2^ force constants to ensure quick convergence of the reaction coordinate to the mean. The simulations are initiated from the same structures with the same velocity seeds to avoid stochastic differences and to isolate errors to differences in the potentials. We perform analysis with umbrella integration as described in the Methods section to avoid noise in the PMFs resulting from performing MBAR analysis on short sampling. This process was also used to correct ΔMACE PMFs to the DFTB3 level to demonstrate that large differences in sampled reaction coordinate means, and therefore PMFs, will be detected within these short simulations (see Figure S30 and Table S4 of the Supporting Information). In addition, we provide a comparison of profiles analyzed by UI, MBAR, and WHAM for the PSr deprotonation reactions in Figure S31.

As shown in Figure 5a, both the G and 2AP proton abstraction reactions in PSr agree well with PBE0. In the MTR1 reaction, there is an approximately 1 kcal·mol^–1^ deviation in the intermediate state and product, but the two profiles overlap very closely. For the VSr reaction, we depart from the ΔMACE-AL model (i.e., perform each set of sampling on the ΔMACE-AL MFEP) and correct it to the PBE0 and ΔMACE level. This is R-SRP that has been specifically refined from the foundational ΔMACE model for the VSr reaction through O-AL in the prior section. This differs from the ΔMACE profile in the previous section because it represents the ΔMACE free energy on the ΔMACE-AL MFEP. The ΔMACE-AL and PBE0 profiles agree quite well, whereas the ΔMACE profile differs by approximately 3 kcal·mol^–1^ in most regions, reflecting the fact that the MFEP did not exactly overlap with that of the ΔMACE-AL model and the accuracy enhancement from active learning. We hypothesize that although the training set force errors are comparable for the PSr, MTR1, and VSr systems, refinding the minimum free energy path with the ΔMLP incurs more degrees of freedom in higher dimensions and therefore allows more opportunity to stray from the training set and perhaps encourages O-AL refinement. These results, taken together with energy and force validation, show a vast improvement from the semiempirical models. This improvement comes with up to 1000x speed up compared to PBE0, with benchmark timings for DFTB3, PBE0, ΔDP, and ΔMACE provided in Table S5 of the Supporting Information. Despite a consistent increase in accuracy compared to the ΔDP model, and comparable GPU timings, one should note that the PyTorch-based ΔMACE model experiences a dramatic drop-off in speed on CPU’s, whereas TensorFlow-based DP models only experience an approximately 10% decrease in speed. In instances where CPU infrastructure is more abundant, and the underlying DFTB3 simulations can be highly parallelized, the ΔDP model could be more desirable, especially for an SRP-based approach.

**Figure 5:**
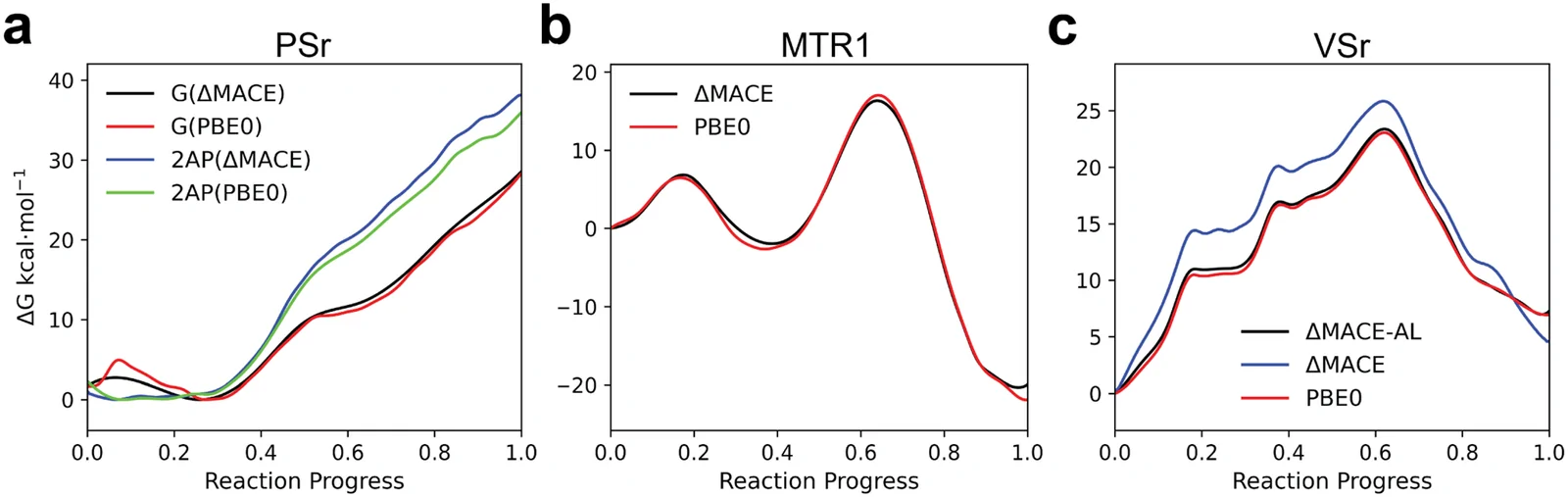
PMF validation of the ΔMACE model compared to the target PBE0 level. PMF for (a) guanine (G) and 2-aminopurine (2AP) deprotonation reactions in the Pistol ribozyme, (b) 2-D methyl transfer reaction in the methyl transferase ribozyme, and (c) 3-D phosphoryl transfer in the Varkud satellite ribozyme.

## Conclusion

We present the ΔNAzyme foundational QM/MM+ΔMLP model for nucleic acid enzyme reactions trained on the newly curated NAzyme data set. This is a fit-for-purpose model to accelerate mechanistic studies of nucleic acid chemistry, with a focus on the computation of experimental observables to promote the design and understanding of new biotechnology. The equivariant, many-body message-passing architecture of the MACE model provides enhanced accuracy and transferability over the popular Deep Potential architecture, making it ideal for a foundational model. The model may be used in place of a semiempirical model for mechanistic exploration and/or rapidly refined for highest accuracy predictions. This lays the groundwork for an approach that could be taken for various enzyme classes: an intermediate between universal potentials that are not amenable to QM/MM simulations, and specific reaction potentials that lack transferability. The NAzyme data set and ΔNAzyme model can be easily expanded as new NAzyme chemistry is evolving rapidly with *in vitro* selection methods and improvements in nucleic acid structure prediction.

## Supplementary Material



## Data Availability

Source data including training input files, the NAzyme data set in HDF5 format, trained ΔNAzyme models, per-system QM/MM preparations (AMBER parameter, topology, mdin, and NMR restraint files), and source data required to reproduce figures and tables are available online through Zenodo: https://doi.org/10.5281/zenodo.21223572.

## Notes

Supporting Figures S1–S31 and Tables S1–S5; system-specific preparation details, semiempirical-level free energy profiles, system-specific parity plots, schematics of case-study reactions, and timing benchmarks (PDF)
