# Supplementary material for: ΔNAzyme: A Foundational QM/MM Δ‑Machine Learning Potential for Nucleic Acid Enzyme Catalysis
Source: J Chem Theory Comput. 2026 Oct 12;22(20):11037–55. doi: 10.1021/acs.jctc.6c01635 (PMC13718545; doi:10.1021/acs.jctc.6c01635)
Supplement: Supplementary file 1 [file ct6c01635_si_001.pdf]

Supporting Information: ΔNAzyme: A foundational QM/MM Δ-machine learning potential for nucleic acid enzyme catalysis

Erika McCarthy,[†] Timothy J. Giese,[†] Şölen Ekesan,[†] Julie Puyo-Fourtine,[†] Lauren Lerew,[†] Andreas W. Götz,[‡] and Darrin M. York[*,†]

†*Laboratory for Biomolecular Simulation Research, Institute for Quantitative Biomedicine and Department of Chemistry and Chemical Biology, Rutgers University, Piscataway, New Jersey 08854, United States*

‡*San Diego Supercomputer Center, University of California San Diego, La Jolla, California 92093-0505, United States*

E-mail: Darrin.York@rutgers.edu

Supporting Information

System-specific preparation

Non-enzymatic reactions

The majority of nucleic acid catalyzed reactions involve phosphoryl transfer. A simple, non-enzymatic (NE) model system of hydroxyalkyl phosphate ester has commonly been employed to gain insight into fundamental aspects of phosphoryl transfer reactions.[S1–S3] In this work we sought to prepare NE model reactions that mimic potential interactions that one may wish to introduce into a ribozyme, such as additional metal ions and phosphorothioate substitutions. The QM region consists of the entire hydroxyalkyl phosphate ester (24 atoms), with some systems containing an additional metal ion and 5 or 6 coordinating waters depending on the coordination pattern (40 or 43 atoms). The metal ions considered were Na^{+}, Mg^{2+}, Ca^{2+}, and Zn^{2+}. Reactions involving 1 and 2 reaction coordinates were sampled:

$$\xi_1 = \mathrm{R}_{\mathrm{O5'-P}} - \mathrm{R}_{\mathrm{O2'-P}} \tag{1}$$

$$\xi_2 = \mathrm{R}_{\mathrm{O5'-H}} - \mathrm{R}_{\mathrm{M.OH-H}} \tag{2}$$

where M.OH is a metal coordinated water donating a proton. 1-dimensional reactions only included ξ_1, thus the O5′ leaves as an oxyanion. The QM region has a -2 charge in the absence of ions, -1 when the metal is Na^{+}, and 0 when the metal is a divalent ion.

HDVr

The 2′-*O*-transphosphorylation reaction catalyzed by the Hepatitis delta virus ribozyme (HDVr) has been explored in depth by our group.[S4] The QM region contained the scissile phosphate, flanking sugars, the C75 nucleobase and sugar, U23 phosphate and flanking sugars, the active site Mg^{2+} ion, and four Mg^{2+} coordinating waters. The reaction path was previously sampled at the AM1/d-phoT level in 3 dimensions including nucleophillic attack, leaving group dissociation, and leaving group deprotonation. To enrich the chemical landscape of the data set, we initiated the DFTB3 reaction

from the ground state in which O2′ is protonated, introducing a fourth reaction coordinate for nucleophile deprotonation and yielding a QM region of 93 atoms and neutral charge (Figure S1). Upon sampling with DFTB3, we observed a ligand substitution event not seen with AM1/d in which the O2′ transitioned from direct coordination to indirect coordination with Mg^{2+}, bringing an adjacent water into the direct coordination sphere. This event was then incorporated as a reaction coordinate, as it was unclear whether this was an artifact of the DFTB3 potential. In total, 5 reaction coordinates were sampled.

$$\xi_1 = \mathrm{R}_{\mathrm{P-O5'}} \tag{3}$$

$$\xi_2 = \mathrm{R}_{\mathrm{P-O2'}} \tag{4}$$

$$\xi_3 = \mathrm{R}_{\mathrm{O5'-H}} - \mathrm{R}_{\mathrm{C75:N3-H}} \tag{5}$$

$$\xi_4 = \mathrm{R}_{\mathrm{OH-H}} - \mathrm{R}_{\mathrm{O2'-H}} \tag{6}$$

$$\xi_5 = \mathrm{R}_{\mathrm{H_2O-Mg^{2+}}} - \mathrm{R}_{\mathrm{O2'-Mg^{2+}}} \tag{7}$$

(8)

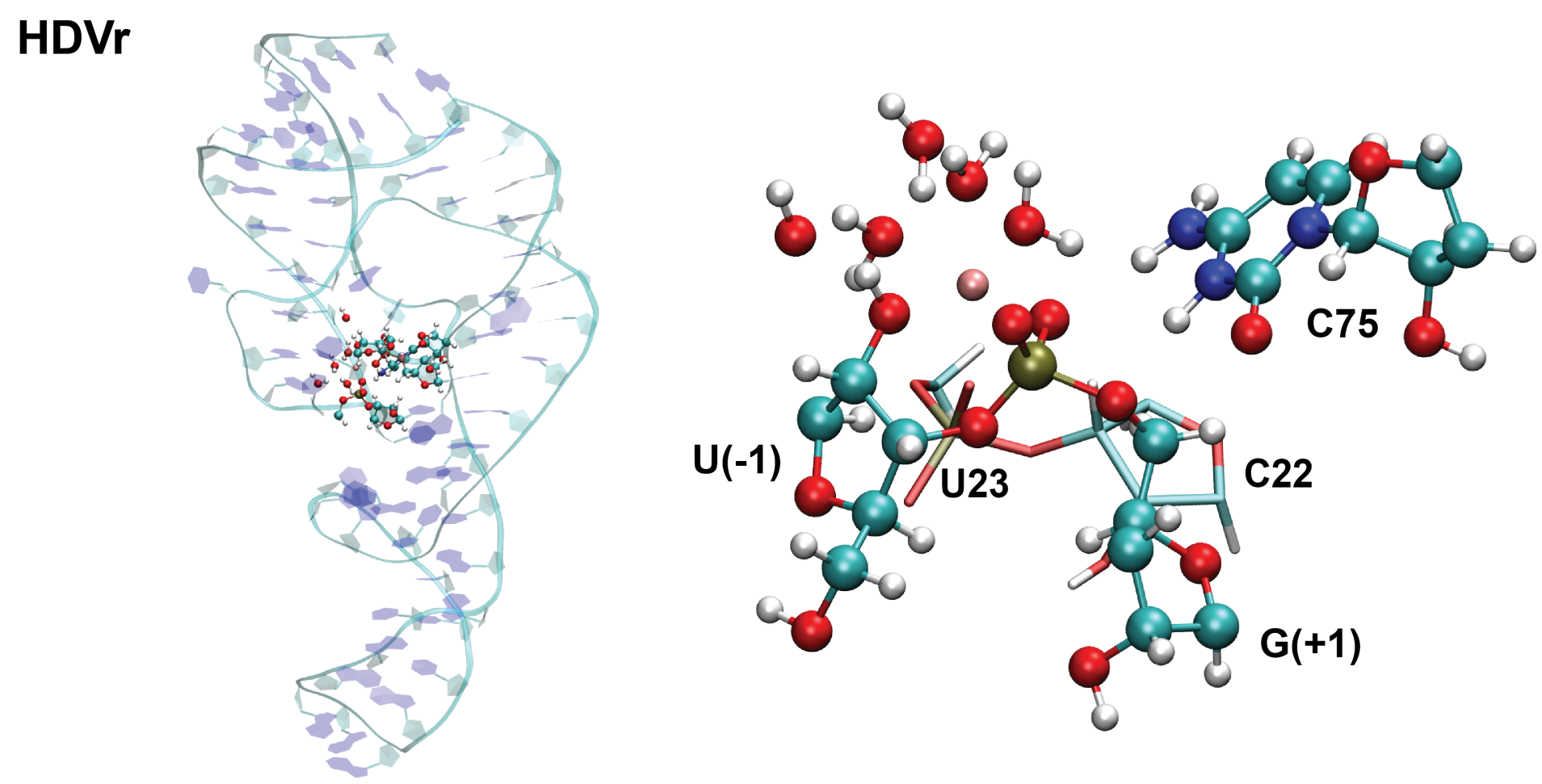

Figure S1: Representative QM region for HDVr reactions. The entire ribozyme is represented by ribbons, with the QM region represented in CPK and licorice for clarity.

To generate a variety of structures, we initiated path-finding from 6 distinct initial guesses coupling different reaction events, each of which evolved into a unique local minima. These included 1) simple linear interpolation, stepwise paths in which the first step coupled 2) O2′ association and

deprotonation (ξ_2 and ξ_4), 3) O2′ association and Mg^{2+} ligand substitution (ξ_2 and ξ_5), 4) O5′ dissociation and Mg^{2+} ligand substitution (ξ_1 and ξ_5), 5) O5′ dissociation and O2′ deprotonation (ξ_1 and ξ_4), and 6) O5′ dissociation and protonation (ξ_1 and ξ_3).

HHr

The Hammerhead ribozyme (HHr) 2′-*O*-transphosphorylation reaction has been extensively simulated.[S5–S11] Specifically, we depart from the equilibrated system described in ref S12. The QM region contains 76 atoms with a net -1 charge. This includes the nucleobase of G12, the scissile phosphate and flanking sugars, the sugar and phosphate of G8, and a Mg^{2+} ion coordinating 4 waters (Figure S2). Reactions where sampled in 3 dimensions in which either the O2′ of G8 or a Mg^{2+} water acts an acid, corresponding to ξ_{3a} and ξ_{3b}, respectively.

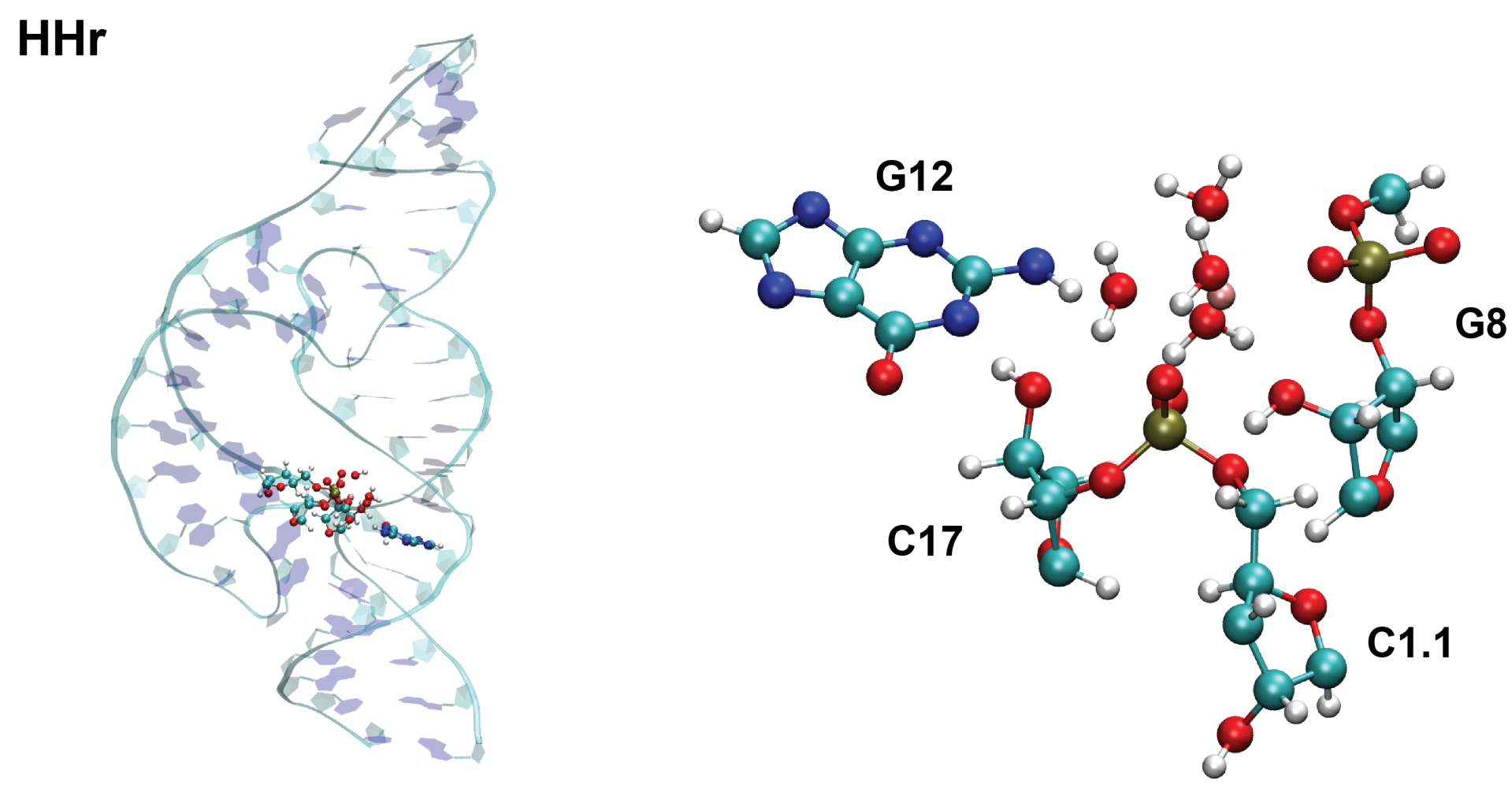

Figure S2: Representative QM region for HHr reactions. The entire ribozyme is represented by ribbons, with the QM region represented in CPK.

$$\xi_1 = \mathrm{R}_{\mathrm{G12:N1-H}} - \mathrm{R}_{\mathrm{C17:O2'-H}} \quad (9)$$

$$\xi_2 = \mathrm{R}_{\mathrm{O5'-P}} - \mathrm{R}_{\mathrm{C17:O2'-P}} \quad (10)$$

$$\xi_{3a} = \mathrm{R}_{\mathrm{O5'-H}} - \mathrm{R}_{\mathrm{G8:O2'-H}} \quad (11)$$

$$\xi_{3b} = \mathrm{R}_{\mathrm{O5'-H}} - \mathrm{R}_{\mathrm{Mg.OH-H}} \quad (12)$$

PSr

The Pistol ribozyme (PSr) represents a less studied system catalyzing 2′-O-transphosphorylation of great interest for further investigation. QM/MM simulations have thus far been limited to the initial proton abstraction step,[S13] which we include here in the data set. DFTB3 sampling with the wild type G42 and modified 2-aminopurine (2AP) were initiated from the equilibrated systems from ref S13, which represent a structure in which G40 is poised to act a general base. The QM region consists of 33 atoms including the scissile phosphate, the N(-1) sugar, and the nucleobase of either G or 2AP (Figure S3). The QM region charge was -2 with G and -1 with 2AP. The proton transfer reaction coordinate is defined as

$$\xi_1 = \mathrm{R}_{\mathrm{G42:N1-H}} - \mathrm{R}_{\mathrm{O2'-H}}. \tag{13}$$

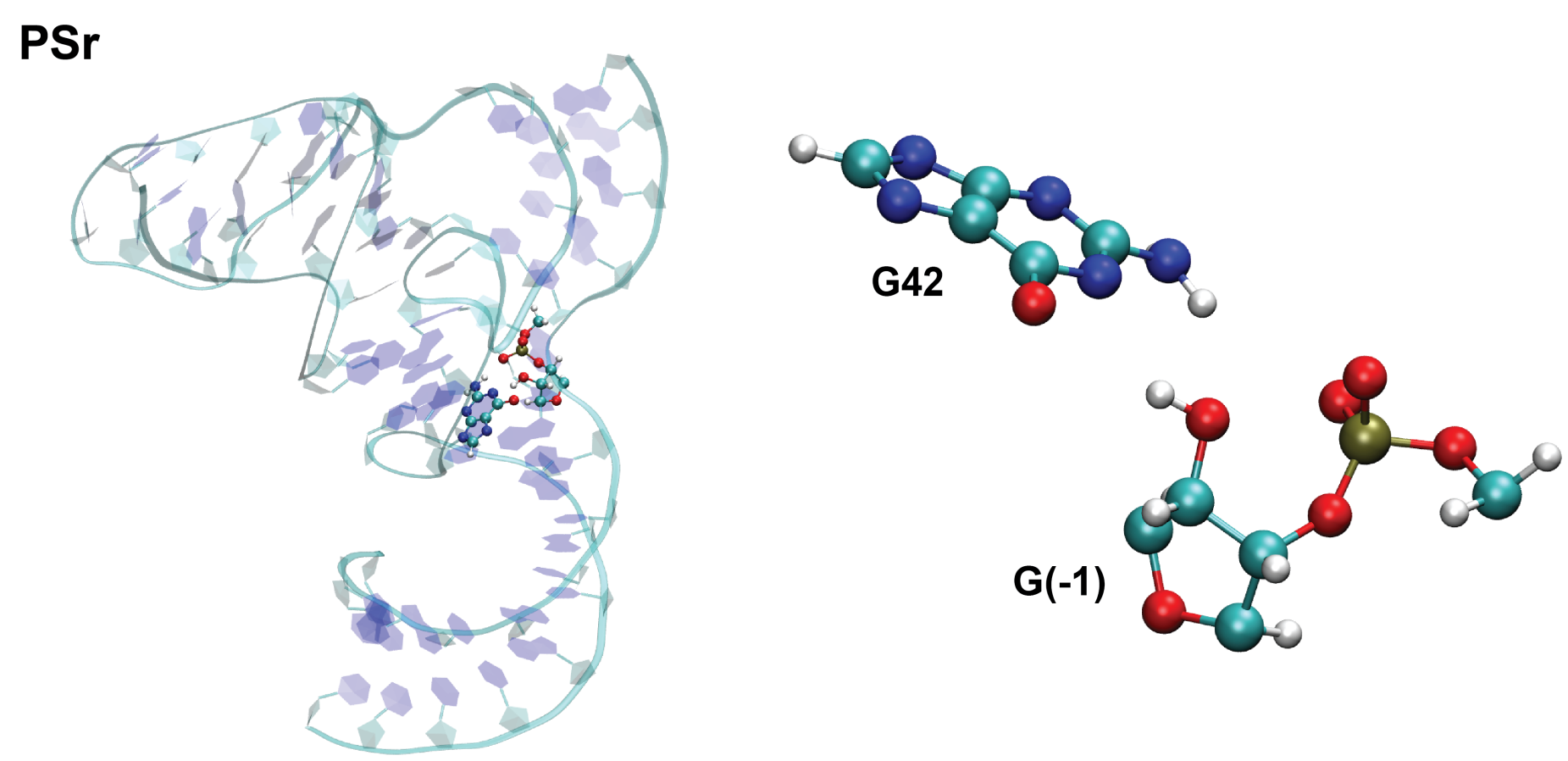

Figure S3: Representative QM region for HHr reactions. The entire ribozyme is represented by ribbons, with the QM region represented in CPK.

VSr

The Varkud satellite ribozyme system was previously prepared and rigorously simulated in ref S14. The 2′-*O*-transphosphorylation was previously sampled at the AM1/d level with QM/MM optimizations performed at the PBE0/6-31G* level. The QM region included 123 atoms including the nucleobases of G623, G638, and A756, the scissile phosphate and flanking sugars, the phosphate at A621, and Mg^{2+} ion with 3 coordinating water yielding neutral charge (Figure S4). In that case, AM1/d simulations were performed in 3 dimensions involving nucleophile deprotonation, phosphoryl transfer, and leaving group protonation, while high level optimizations were performed in the latter two dimensions. In this work, we re-perform sampling at the DFTB3 level in 3 dimensions defined by the following reaction coordinates:

$$\xi_1 = \mathrm{R}_{\mathrm{G638:N1-H}} - \mathrm{R}_{\mathrm{O2'-H}} \tag{14}$$

$$\xi_2 = \mathrm{R}_{\mathrm{O5'-P}} - \mathrm{R}_{\mathrm{O2'-P}} \tag{15}$$

$$\xi_3 = \mathrm{R}_{\mathrm{O5'-H}} - \mathrm{R}_{\mathrm{A756:N1-H}}. \tag{16}$$

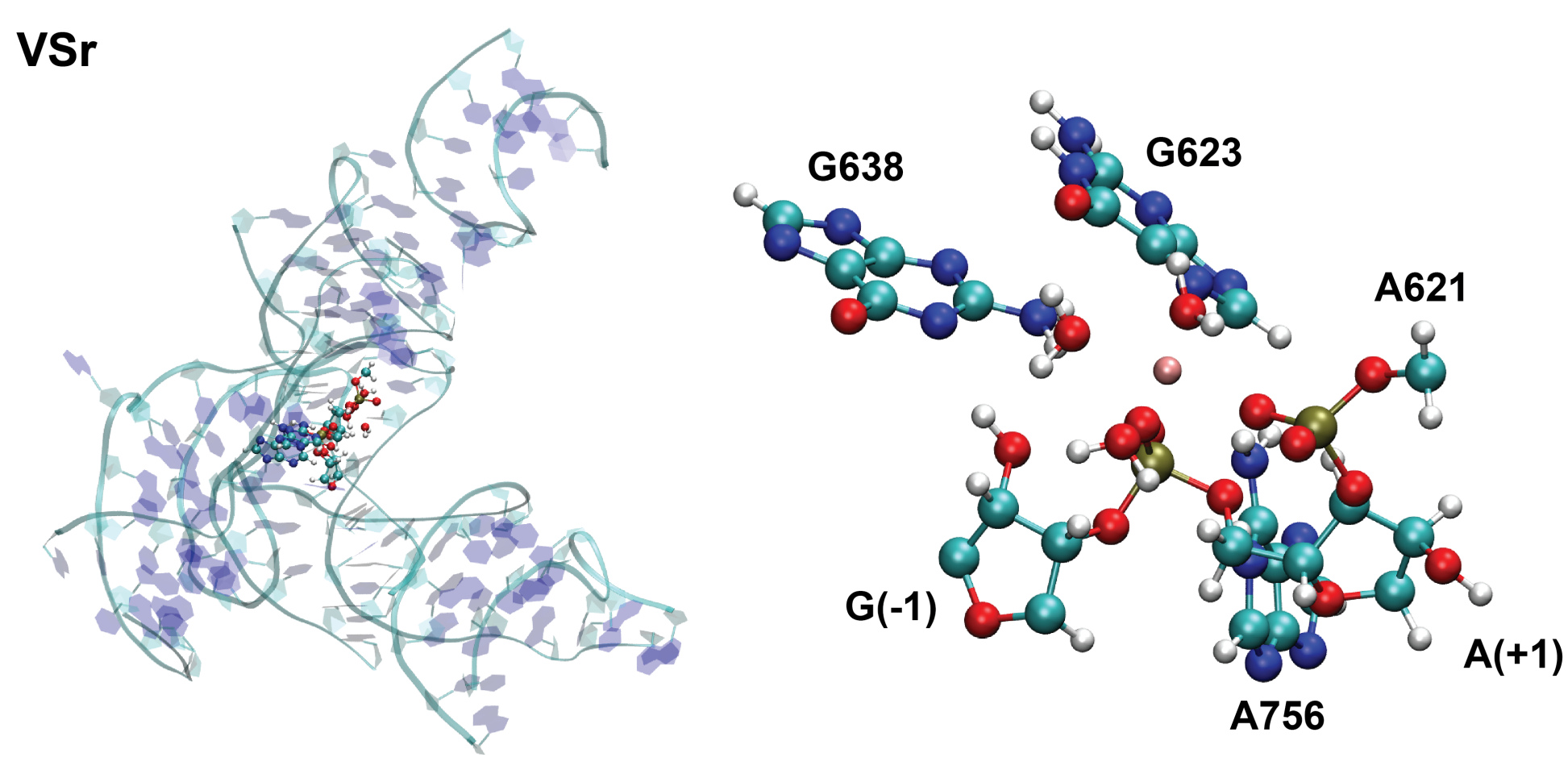

Figure S4: Representative QM region for VSr reactions. The entire ribozyme is represented by ribbons, with the QM region represented in CPK.

TWr

The 2′-*O*-transphosphorylation reaction of the Twister ribozyme (TWr) was previously investigated in ref S15. In this work, a reactant state QM/MM ensemble was generated using DFTB3, predominately to ensure the model was trained on structures containing adenine protonated at the N3 position, a protonation state not present in other ribozymes in the data set. The QM region contained 78 atoms including the nucleobase of G33 and the two nucleotides flanking the scissile phosphate with a total charge of -2 (Figure S5).

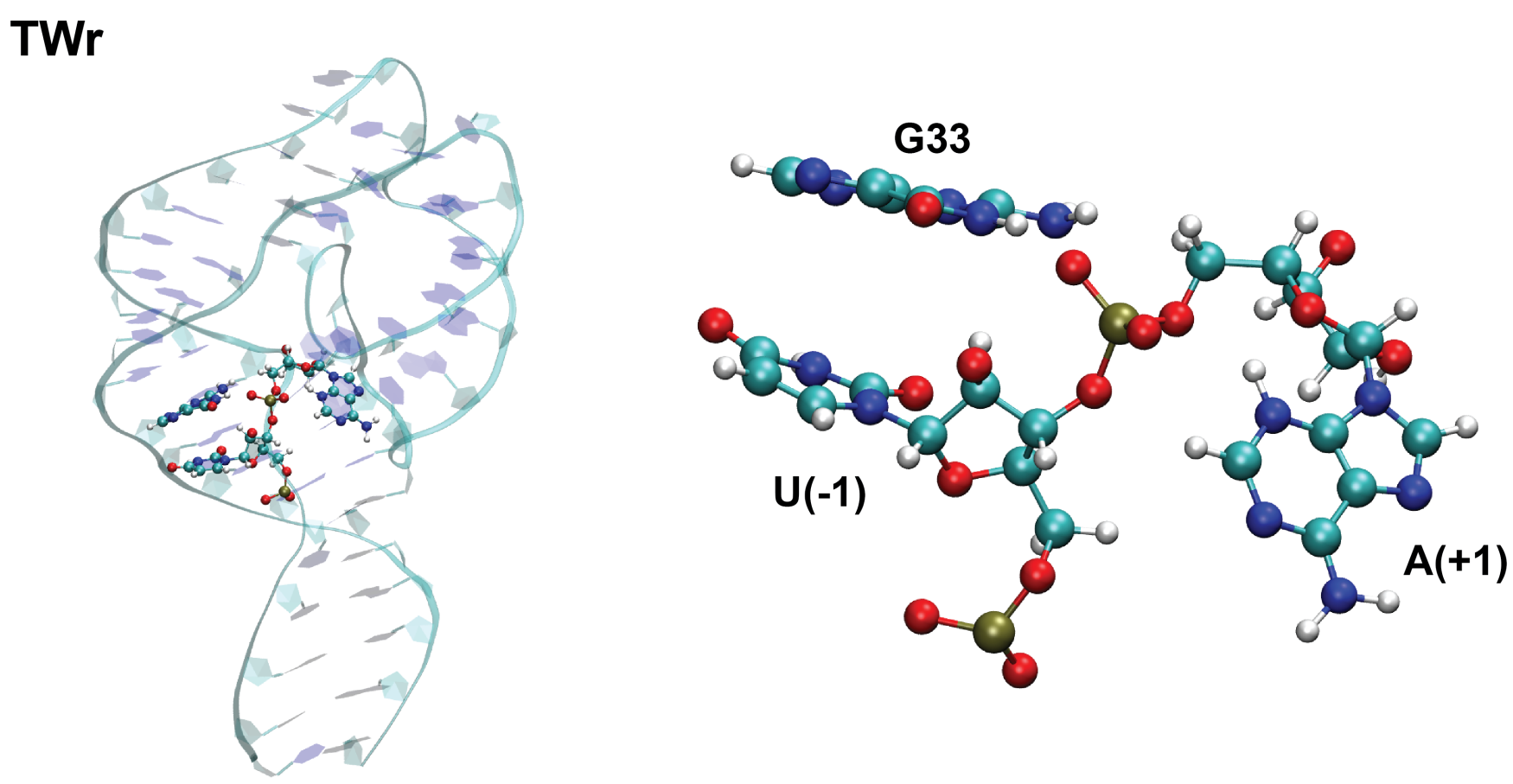

Figure S5: Representative QM region for of TWr. The entire ribozyme is represented by ribbons, with the QM region represented in CPK.

8-17dz

The 8-17 dnazyme (8-17dz) also catalyzes 2′-*O*-transphosphorylation to cleave the RNA backbone, but with an all DNA catalyst. Molecular dynamics simulations of 8-17dz were initially performed in ref S16 with Pb^{2+} bound at the active site. However, understanding the chemistry of 8-17dz in the presence of non-toxic metals including Mg^{2+}, Ca^{2+}, and Zn^{2+} is of great interest. Although a full mechanistic investigation is outside the scope of this work, we sought to include ensembles from the 8-17dz reactant state in the data set to inform the model of QM/MM interactions with a DNA environment to enhance future transferability. Toward this end, 3 reactant state systems were simulated with Pb^{2+} substituted by Mg^{2+}, Ca^{2+}, and Zn^{2+} using DFTB3. The QM region included 71 atoms with neutral charge comprising nucleobase of G13 and G8, the scissile phosphate and

flanking sugars, and the metal ion with 4 coordinating waters (Figure S6). No reaction coordinate was used, as simulations were limited to the reactant state.

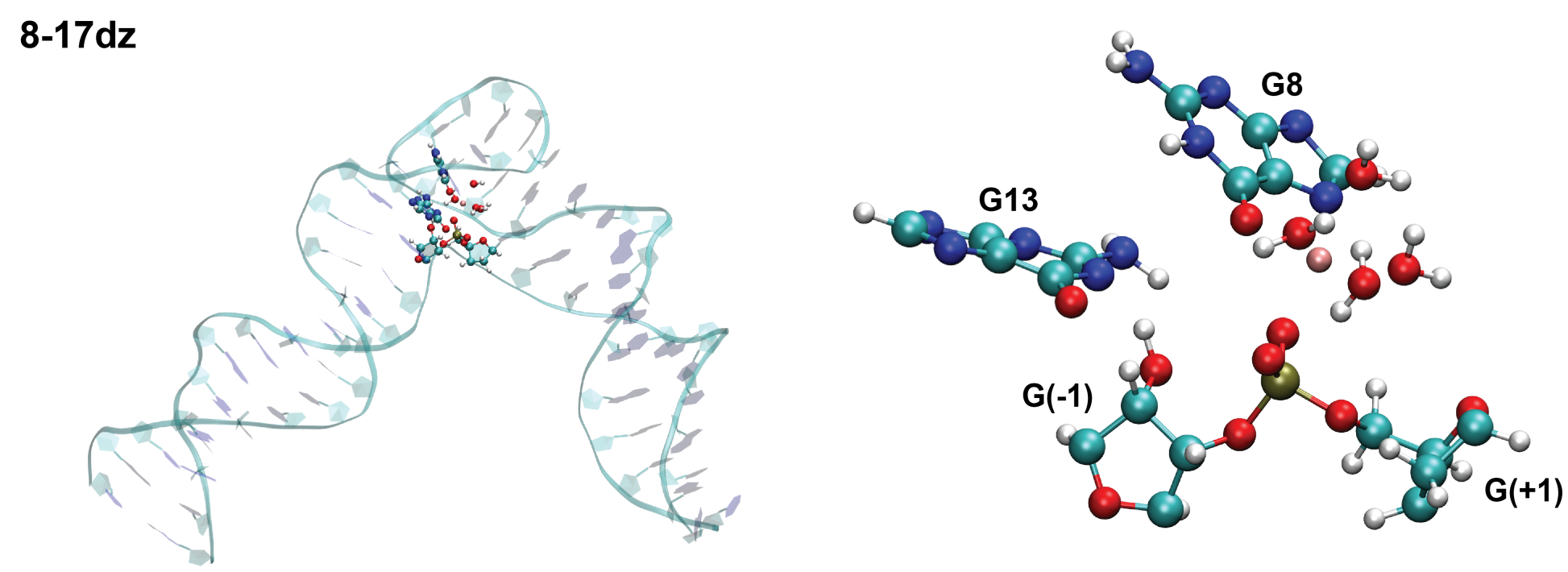

Figure S6: Representative QM region for 8-17dz. The entire ribozyme is represented by ribbons, with the QM region represented in CPK.

RNase A

The bovine pancreatic ribonuclease A (RNase A) is a protein enzyme that catalyzes the cleavage of RNA through two steps; a 2′-*O*-transphosphorylation reaction, forming a 2′,3′-cyclic phosphate intermediate, which is then hydrolyzed to produce a 3′-*O* phosphate product.[S17] In this work we model the transphosphorylation step in three reaction coordinates. During transphosphorylation, RNase A utilizes the side chain residue His12 as a general base to activate the O2′ by transferring HO2′ (ξ_1). The O2′ attacks the phosphorus center amid O5′ departure (ξ_2), and the O5′ leaving group is protonated by an additional side chain residue, His119, acting as a general acid (ξ_3).

$$\xi_1 = \mathrm{R}_{\text{H-O2}'} - \mathrm{R}_{\text{H-His12:N}} \tag{17}$$

$$\xi_2 = \mathrm{R}_{\text{P-O5}'} - \mathrm{R}_{\text{P-O2}'} \tag{18}$$

$$\xi_3 = \mathrm{R}_{\text{H-His119:N}} - \mathrm{R}_{\text{H-O5}'} \tag{19}$$

(20)

The 117 atom QM region includes His12, His119, Lys41, cytidylyl-(3′,5′)-adenosine (CpA), and the coordinated water molecule, with a +1 charge (Figure S7).

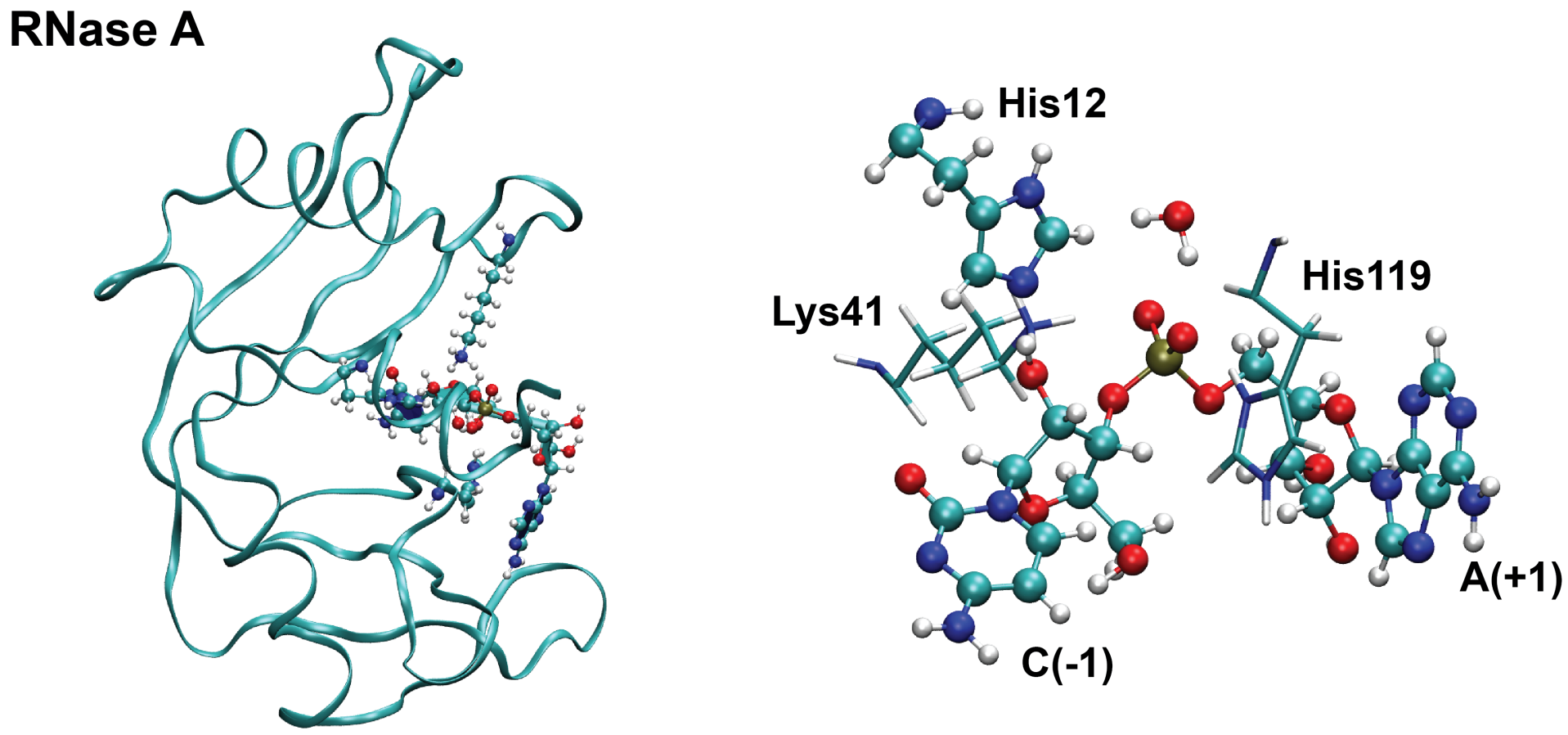

Figure S7: Representative QM region for RNase A reactions. The entire ribozyme is represented by ribbons, with the QM region represented in CPK and licorice for clarity.

THr

The Tetrahymena ribozyme (THr) catalyzes its own excision and subsequent ligation of neighboring exons through two sequential transesterification reactions, a process known as splicing.[S18] The first step of the reaction requires binding of an exogenous guanine cofactor which cleaves at the 5′ splice site and attaches itself to the 5′ intron. After a conformational change, the 5′ residue attacks the 3′ splice site to form a ligated exon. Multiple mechanistic scenarios were probed for both the cleavage and ligation steps, including the presence and absence of a third active site metal ion. For both reactions, the QM regions included the nucleophile and leaving group sugars, the scissile phosphate, 2 or 3 metal ions (with the third being either Na^+ or Mg^{2+}), waters directly coordinated to the metal ions, and the phosphate groups of C262, A306, A304, and C208. This totaled 77 QM atoms for the 2 metal system and 90 for 3 metal systems. The QM region net charges were -2 for the 2 metal system, -1 if the third metal was Na^+, and 0 when it was Mg^{2+}. The reaction coordinates were defined as

$$\xi_1 = \mathbf{R}_{\text{P-U:O3}'} - \mathbf{R}_{\text{P-G:O3}'} \tag{21}$$

$$\xi_2 = \mathbf{R}_{\text{G:O3}'\text{-HO3}'} - \mathbf{R}_{\text{OH-HO3}'} \tag{22}$$

$$\xi_3 = \mathbf{R}_{\text{X-H}} - \mathbf{R}_{\text{U:O3}'\text{-H}}, \tag{23}$$

corresponding to phosphoryl transfer, nucleophile deprotonation, and leaving group protonation, respectively. An "X" indicates the identity of the acid, which is varied across the mechanistic scenarios including the O2′ of the leaving group, water from the bulk, or metal coordinated waters. The scenario with no acid omits ξ_3.

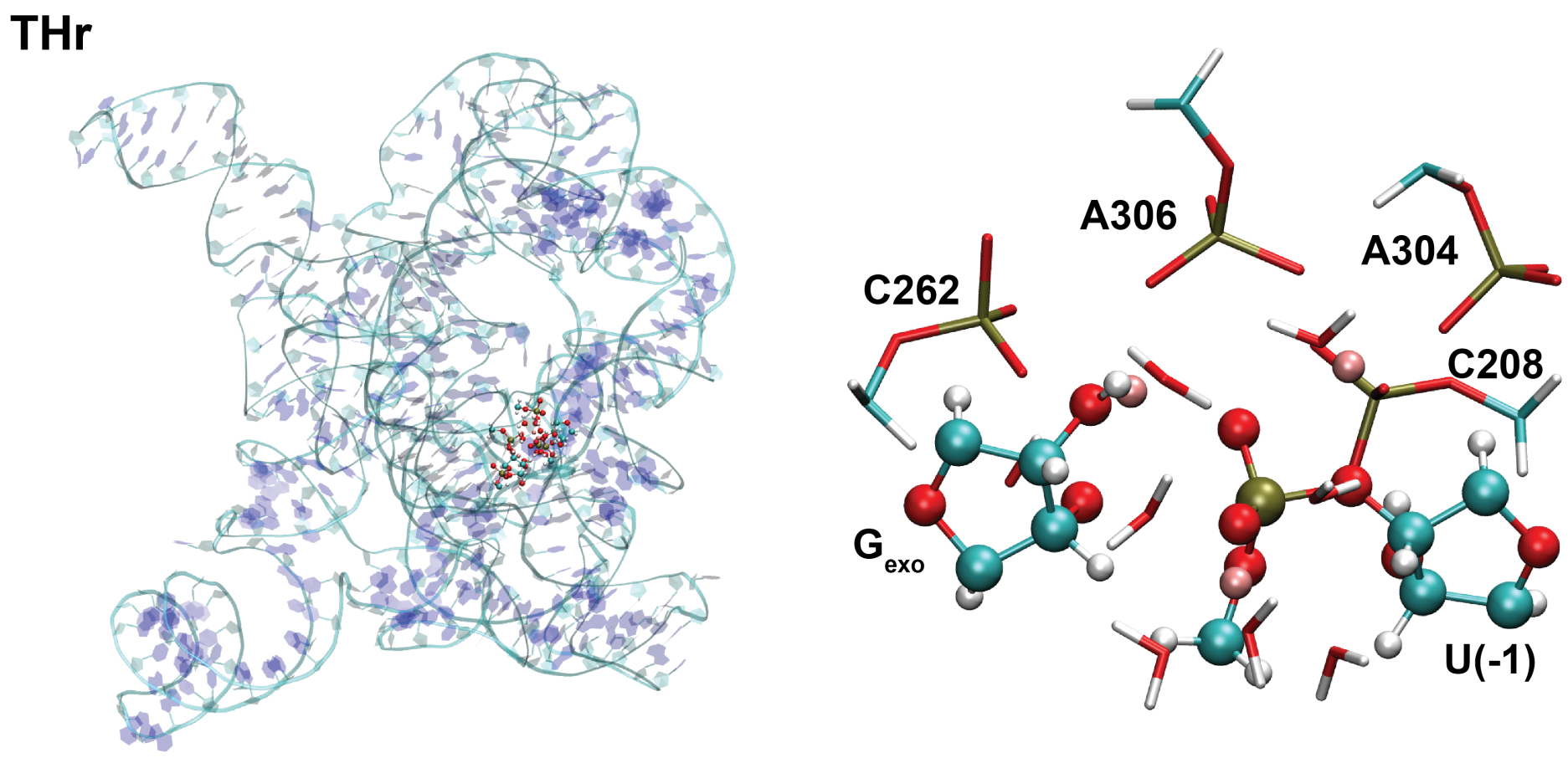

Figure S8: Representative QM region for THr reactions. The entire ribozyme is represented by ribbons, with the QM region represented in CPK and licorice for clarity.

MTR1

DFTB3 sampling of the methyl transferase ribozyme (MTR1) alkyl transfer reaction was previously performed with the O^6-methyl guanine and O^6-(4-aminomethyl-benzyl) guanine cofactors.[S19,S20] The QM regions contained 46 and 62 atoms, respectively, comprising the cofactor, C10 nucleobase, and A63 nucleobase. The QM region charges were +1 and +2, respectively. Reactions of isofunctionally modified ribozymes including C10(n1c), C10(n1c,c5n) (also referred to as ΨisoC), and A63(n7c) were also taken from ref S20, with C10(n1c) having a neutrally charged QM region. An additional system was prepared where the entire substrate strand was mutated to DNA, which did not impact the QM region. The reaction coordinates included proton transfer (ξ_1) and alkyl transfer (ξ_2).

$$\xi_1 = \mathrm{R}_{\mathrm{O^6alkG:N1-H}} - \mathrm{R}_{\mathrm{C10:N3-H}} \tag{24}$$

$$\xi_2 = \mathrm{R}_{\mathrm{A63:N1-CR}} - \mathrm{R}_{\mathrm{O^6alkG:O6-CR}} \tag{25}$$

CR indicates the alkyl carbon.

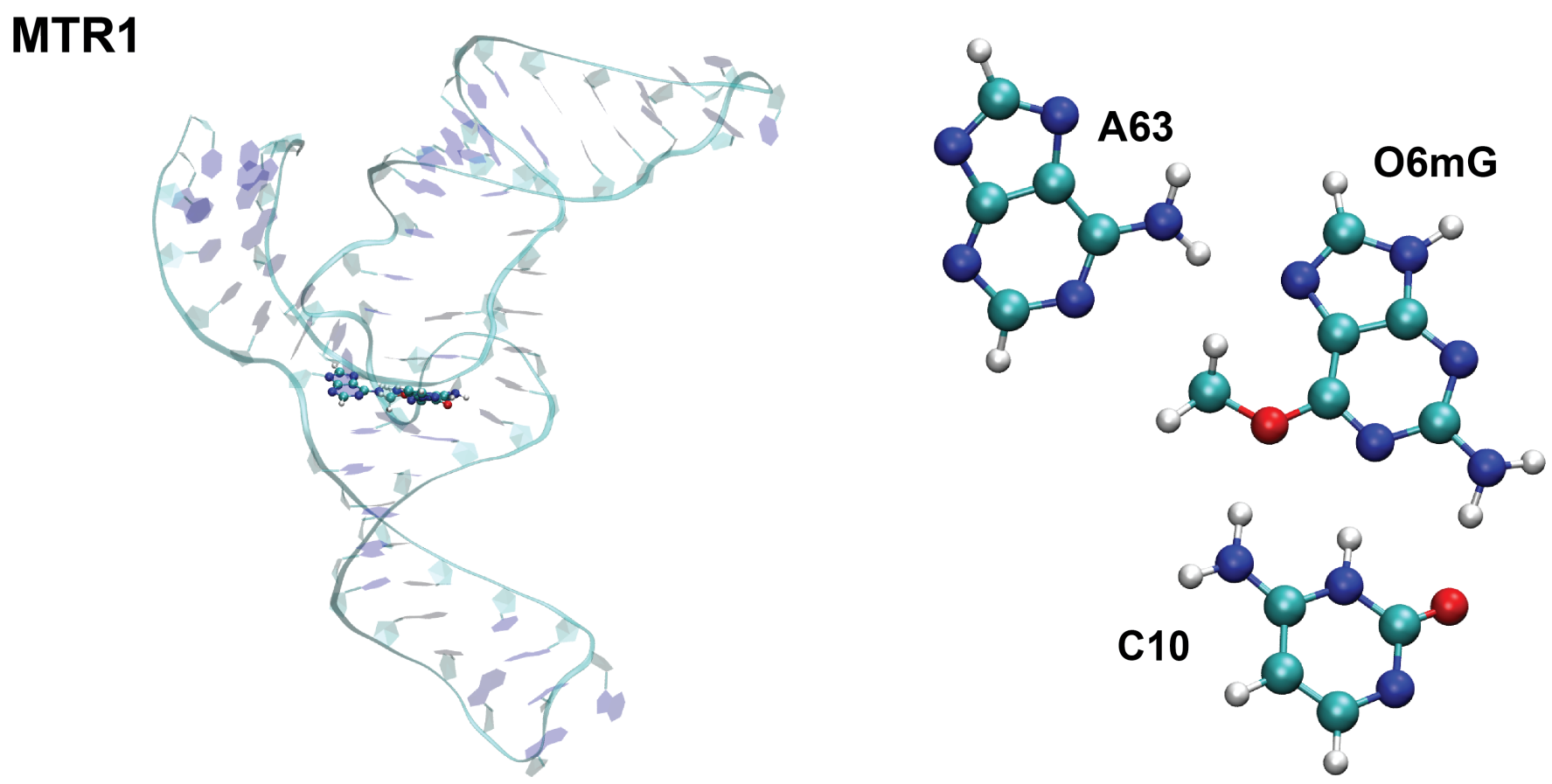

Figure S9: Representative QM region for MTR1 reactions. The entire ribozyme is represented by ribbons, with the QM region represented in CPK.

SAR

The SAR system preparation and MD simulations have been described previously in ref S21. The 3 mechanisms probed involve a "baseline" mechanism where a nucleophilic guanine attacks the epoxide ligand to open the ring, yielding an oxyanion product. In addition, mechanisms involving protonation of the epoxide by a Mg^{2+} coordinated water ("Mg^{2+}") and by the O6 hydrogen of a guanine tautomer ("Taut") were considered. The baseline mechanism contains 30 QM atoms including the G16 nucleobase and epoxide ligand and is neutrally charged. The QM region for the Mg^{2+} mechanism is the same as the baseline with the addition of a Mg^{2+}, 5 coordinating waters, and the phosphate group of G16 totaling 59 atoms with a net charge of +2. The QM region for the Taut mechanism is the same as the baseline with the addition of the C46 nucleobase, totaling 43 atoms with a +1 net charge. The reactions are sampled in 1 to 3 dimensions as follows:

$$\text{Baseline:} \tag{26}$$

$$\xi_1 = \mathrm{R}_{\text{C22-O21}} - \mathrm{R}_{\text{C22-G16:N7}} \tag{27}$$

$$\mathrm{Mg}^{2+}: \tag{28}$$

$$\xi_2 = \mathrm{R}_{\text{H-O21}} - \mathrm{R}_{\text{H-Mg}^{2+}\text{OH}} \tag{29}$$

$$\text{Taut}: \tag{30}$$

$$\xi_2 = \mathrm{R}_{\text{H-O21}} - \mathrm{R}_{\text{H-G16:O6}} \tag{31}$$

$$\xi_3 = \mathrm{R}_{\text{H-G16:N1}} - \mathrm{R}_{\text{H-C46:N3}} \tag{32}$$

where ξ_1 represents nucleophillic attack and epoxide ring opening in each reaction, ξ_2 represents protonation of the epoxide, and ξ_3 represents proton transfer from C46 to G16 to recover Watson Crick pairing.

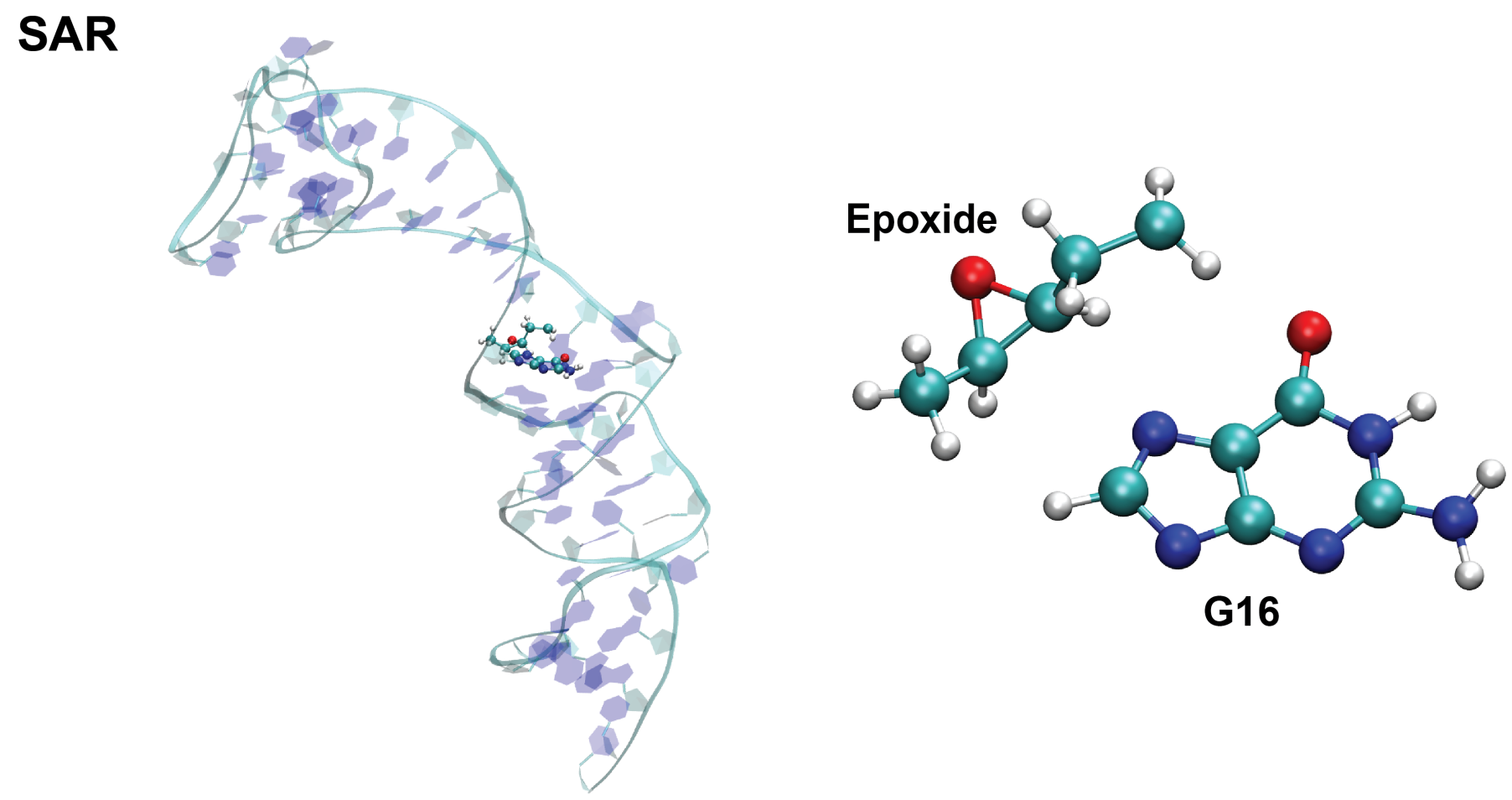

Figure S10: Representative QM region for the baseline SAR reaction. The entire ribozyme is represented by ribbons, with the QM region represented in CPK.

SAMURI

System preparation, equilibration and MD simulation protocols have been described previously in ref S22. The SAMURI ribozyme catalyzes alkyl transfer from *S*-adenosyl-L-methionine (SAM) to a nucleobase. Here, methyl transfer from SAM to A52 at N3 was studied, yielding N3-methyladenosine

and S-adenosyl-L-homocysteine (SAH). The quantum region was defined to capture the reactive center, the directly participating nucleobase, and key electrostatic interactions stabilizing the SAM tail. The QM region contained 59 atoms and included the A52 nucleobase, SAM cofactor excluding the ribose moiety (truncated between C5′ and C4′), a Mg^{2+} ion coordinating the cofactor tail and 4 water molecules, and the phosphate group of G30. The reaction mechanism was investigated using a single reaction coordinate describing the methyl transfer:

$$\xi_{\mathrm{MT}} = \mathrm{R}_{\mathrm{SAM:S-C_m}} - \mathrm{R}_{\mathrm{A52:N3-C_m}}, \tag{33}$$

where SAM:S is the SAM sulfur atom, $\mathrm{C_m}$ is the transferring methyl carbon, and A52:N3 is the nucleophillic N3 atom of A52.

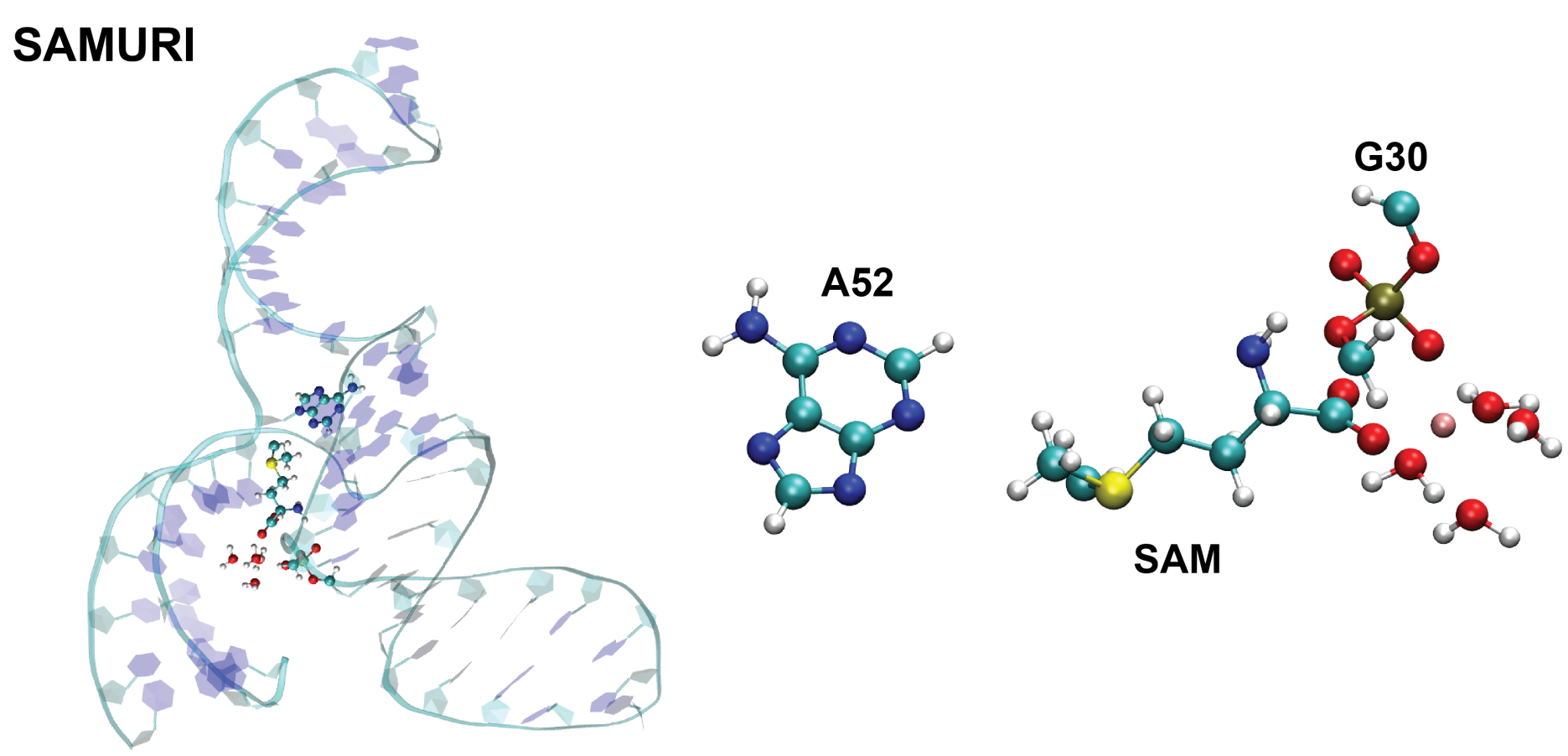

Figure S11: Representative QM region for the SAMURI reaction. The entire ribozyme is represented by ribbons, with the QM region represented in CPK.

Supplementary results

Comparison of free energy profiles

We use reactions of PSr, MTR1, and VSr to benchmark the performance of some popular semiempirical methods and the QDπ model compared to PBE0 for free energy profiles. Schematics of these reactions are given in Figure S12. Table S1 provides key details for free energy profiles presented in Figure 1 of the main text. Across the reactions, it is difficult to find a consensus approach that consistently agrees with the target PBE0.

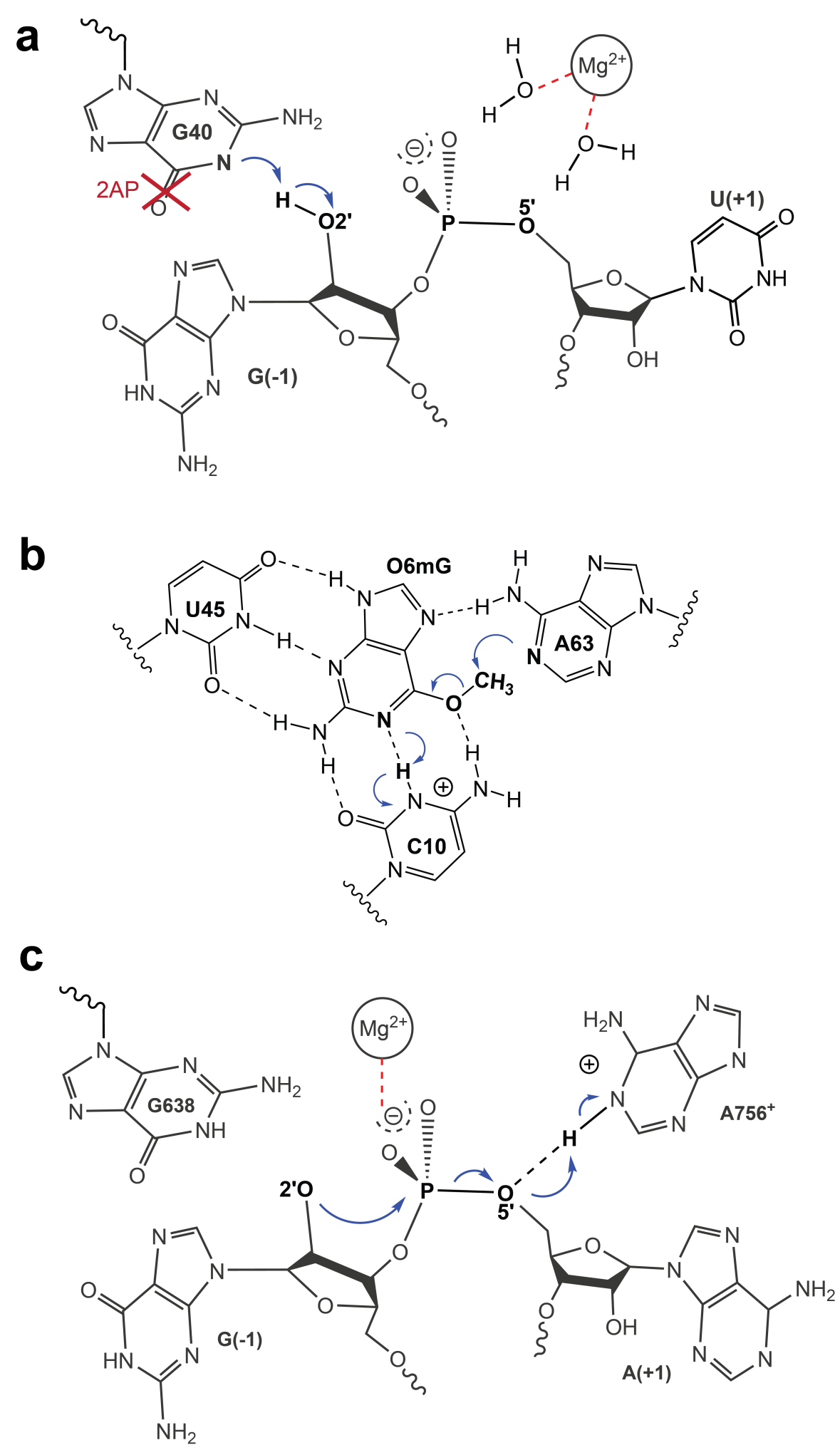

Figure S12: Schematics of reactions used to benchmark the forces of trial semiempirical methods. **(a)** Deprotonation of the nucleophile in PSr by G and 2AP, which lacks the O6, sampled in 1 dimension. **(b)** Proton transfer from C10 to O^6mG and methyl transfer from O^6mG to A63 in MTR1 sampled in 2 dimensions. **(c)** Phosphoryl transfer reaction and leaving group protonation by A756 in VSr departing from an activated nucleophile sampled in 2 dimensions.

In addition, we tested the DFTB3/3ob specific reaction parameterizations nhmod and ophyd, which are intended to improve the descriptions of proton transfer to sp3 nitrogen and pentavalent phosphate transition states, respectively. The resulting free energy profiles are given in Figure S13. While the DFTB3/3ob:ophyd parameterization brought the VSr profile closer to PBE0, the nhmod method did not significantly improve the proton transfer reactions of PSr, and MTR1. While semiempirical SRP's can be valuable for specific applications, as will be show in a later case study, many NAzyme reactions involve both proton and phosphoryl transfers, so a single approach does not correct both simultaneously.

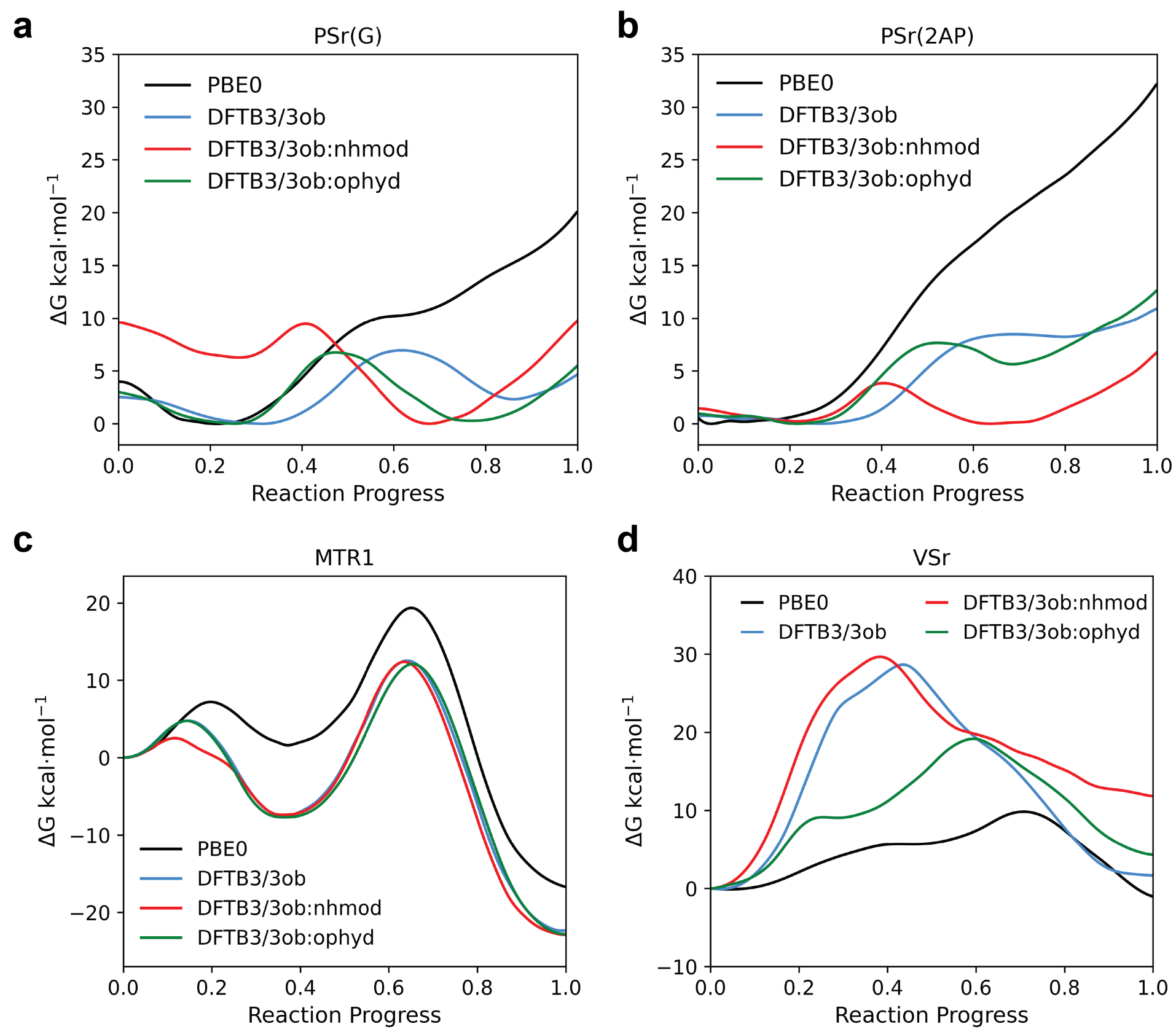

Figure S13: Free energy profiles obtained from QM/MM simulations at DFTB3/3ob, DFTB3/3ob:nhmod, DFTB3/3ob:ophyd, and PBE0/6-31G* levels of theory. Deprotonation of the nucleophile O2′ by general base **(a)** G and **(b)** 2AP in PSr. **(c)** Two-step proton and methyl transfer reaction of MTR1. **(d)** Phosphoryl transfer and leaving group protonation in VSr.

Table S1: Free energy barriers for reactions of PSr, MTR1, and VSr with trial potentials

			(kcal·mol^{-1})	(Å)		(kcal·mol^{-1})
System	Potential	N(rcs)	$\Delta G^{\ddagger}$	$\xi_1^{\ddagger}$	$\xi_2^{\ddagger}$	ΔG_{rxn}
PSr(G)	PBE0	1	16.29	2.00	-	16.29
	AM1/d	1	13.22	0.13	-	11.91
	DFTB3	1	6.95	-0.10	-	2.32
	GFN2-xTB	1	2.15	-0.03	-	0.00
	QDπ	1	9.20	0.00	-	2.53
	MACE-OFF(M)	1	4.87	-0.38	-	-9.54
PSr(AP)	PBE0	1	25.97	2.00	-	25.97
	AM1/d	1	22.82	0.63	-	22.82
	DFTB3	1	8.48	0.15	-	9.15
	GFN2-xTB	1	16.88	0.44	-	16.88
	QDπ	1	12.20	0.79	-	8.29
	MACE-OFF(M)	1	25.30	2.00	-	25.30
MTR1	PBE0	2	19.36	0.89	-0.22	-16.71
	AM1/d	2	41.12	0.90	-0.13	-7.16
	DFTB3	2	12.53	0.94	-0.24	-22.36
	GFN2-xTB	2	18.61	0.72	-0.19	-19.52
	QDπ	2	18.87	0.92	-0.32	-22.02
	MACE-OFF(M)	2	57.08	0.96	-0.46	-10.67
VSr	PBE0	2	9.74	0.14	0.08	-1.02
	AM1/d	2	24.77	0.38	-0.19	2.56
	DFTB3	2	28.66	0.35	-0.57	1.68
	GFN2-xTB	2	7.07	0.33	-0.04	-0.33

Free energies correspond to the profiles shown in Figure 1. N(rcs) is the number of reaction coordinates used to sample the pathway. $\Delta G^{\ddagger}$ is the rate controlling free energy barrier, with corresponding reaction coordinates ξ_1 and ξ_2 when applicable. These are proton abstraction in PSr, proton transfer and methyl transfer in MTR1, and phosphoryl transfer and leaving group protonation in VSr. ΔG_{rxn} is the free energy of reaction.

Evaluation of semiempirical models

Benchmarking force evaluations were performed for 4 chemically distinct reactions to inform the choice of underlying semiempirical method. Schematics of the reactions are given in Figure S14. Forces were evaluated for structures along a pathway linearly interpolating between reactant and product. Figure S15 compares the DFTB3, AM1/d, and GFN2-xTB (reference) forces to those obtained with PBE0. The HDVr plot contains 160 less structures than the other three due to self-consistent field failures in the reactant and product regions.

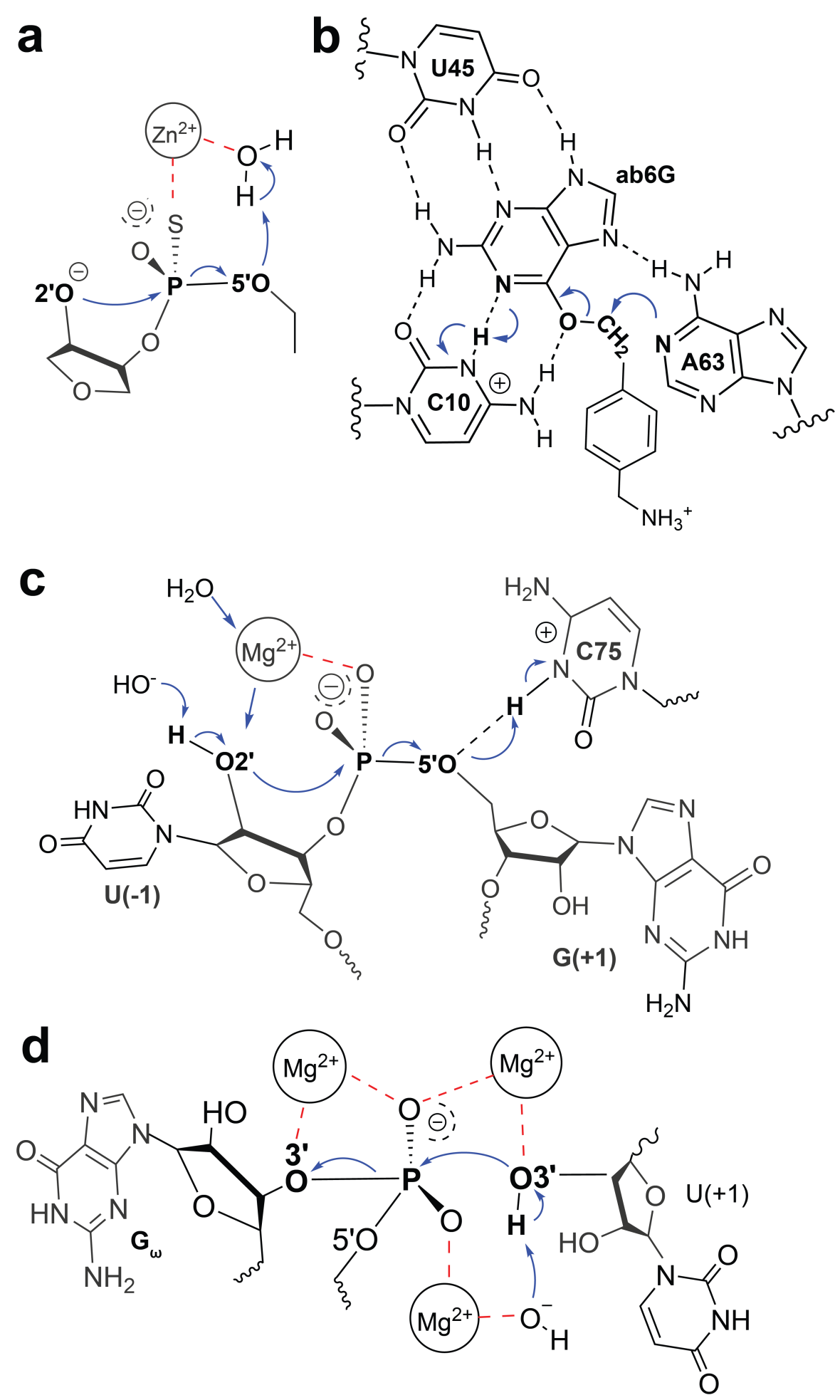

Figure S14: Schematics of reactions used as test sets to benchmark semiempirical methods and transferability of ΔNAzyme models. **(a)** Non-enzymatic model phosphoryl transfer reaction catalyzed by Zn^{2+} with the pro-S_P oxygen substituted for sulfur. **(b)** Proton transfer from C10 to ab6G and alkyl transfer from ab6G to A63 in MTR1 sampled in 2 dimensions. **(c)** RNA cleavage reaction in HDVr sampled in 5 dimensions including nucleophile activation, nucleophillic attack, leaving group departure, leaving group protonation, and Mg^{2+} ligand substitution as O2′ becomes indirectly coordinated. **(d)** Ligation reaction of THr involving nucleophile deprotonation by Mg^{2+} coordinated hydroxide and phosphoryl transfer.

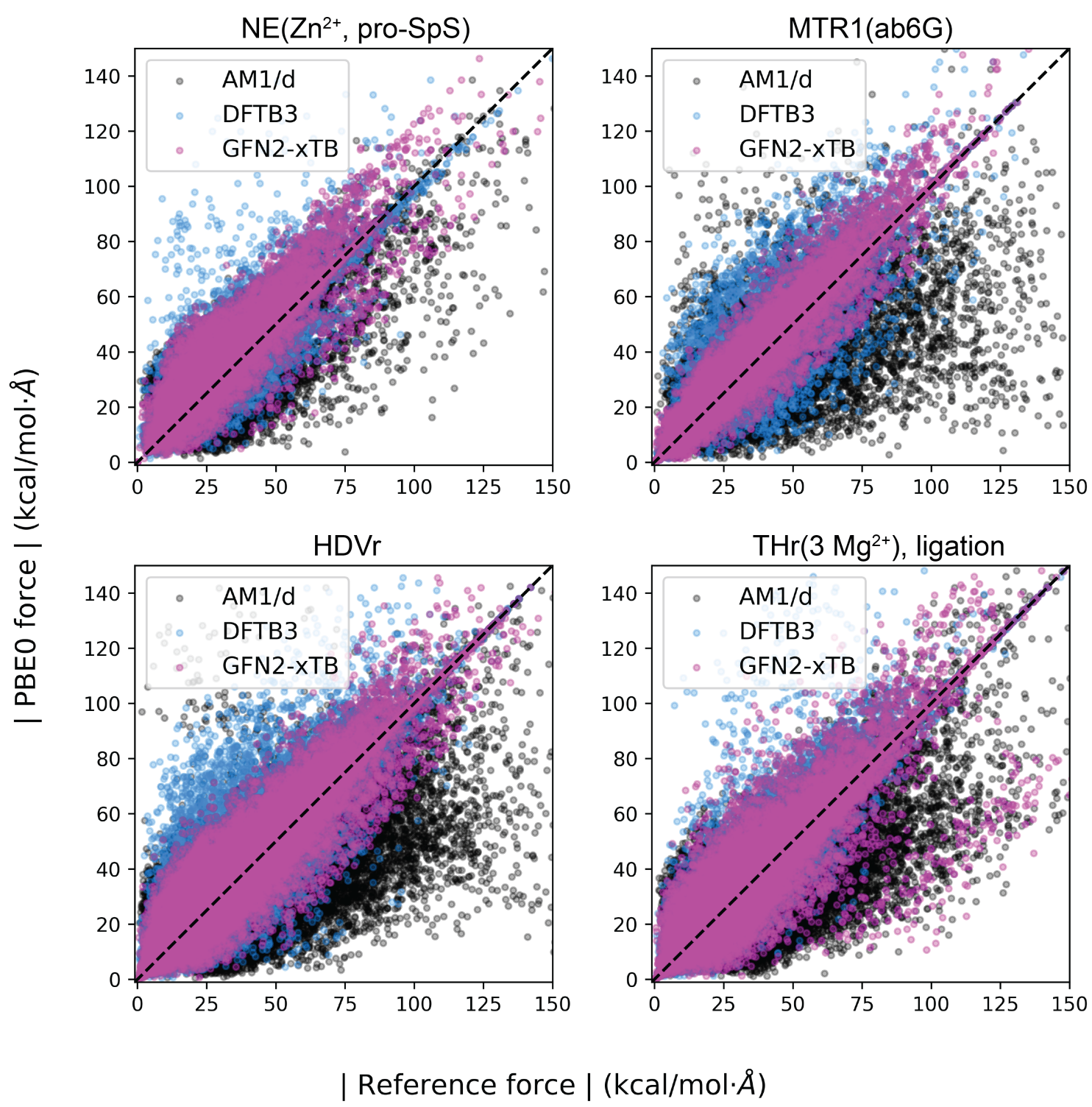

Figure S15: Comparison of the magnitude of forces obtained with AM1/d, DFTB3, and GFN2-xTB compared to the target, PBE0, for non-enzymatic phosphoryl transfer with Zn^{2+} coordinating the pro-S_P sulfur acting as an acid, alkyl transfer of in MTR1 with the ab6G ligand, RNA cleavage by HDV, and ligation by THr with 3 active site metal ions.

DFTB3 level free energy profiles generated from curation of the NAzyme data set

In the process of generating the NAzyme data set, DFTB3 level path-finding was performed, and the associated free energy profiles are provided in Figures S16-S20. Across the DFTB3 profiles, there are various results that raise concerns about DFTB3, especially for phosphoryl transfer reactions. For non-enzymatic reactions with a metal ion acting as an acid, one would expect the ion with the lowest pK_a (Zn^{2+}) for a coordinating water to yield the lowest barrier and most stable product. The ions in order of highest to lowest pK_a are $Na^+ > Ca^{2+} > Mg^{2+} > Zn^{2+}$; however, the product minima follow the order of $Ca^{2+} > Zn^{2+} > Mg^{2+} > Na^+$. This order, particularly the Na^+ result, is difficult to interpret chemically.

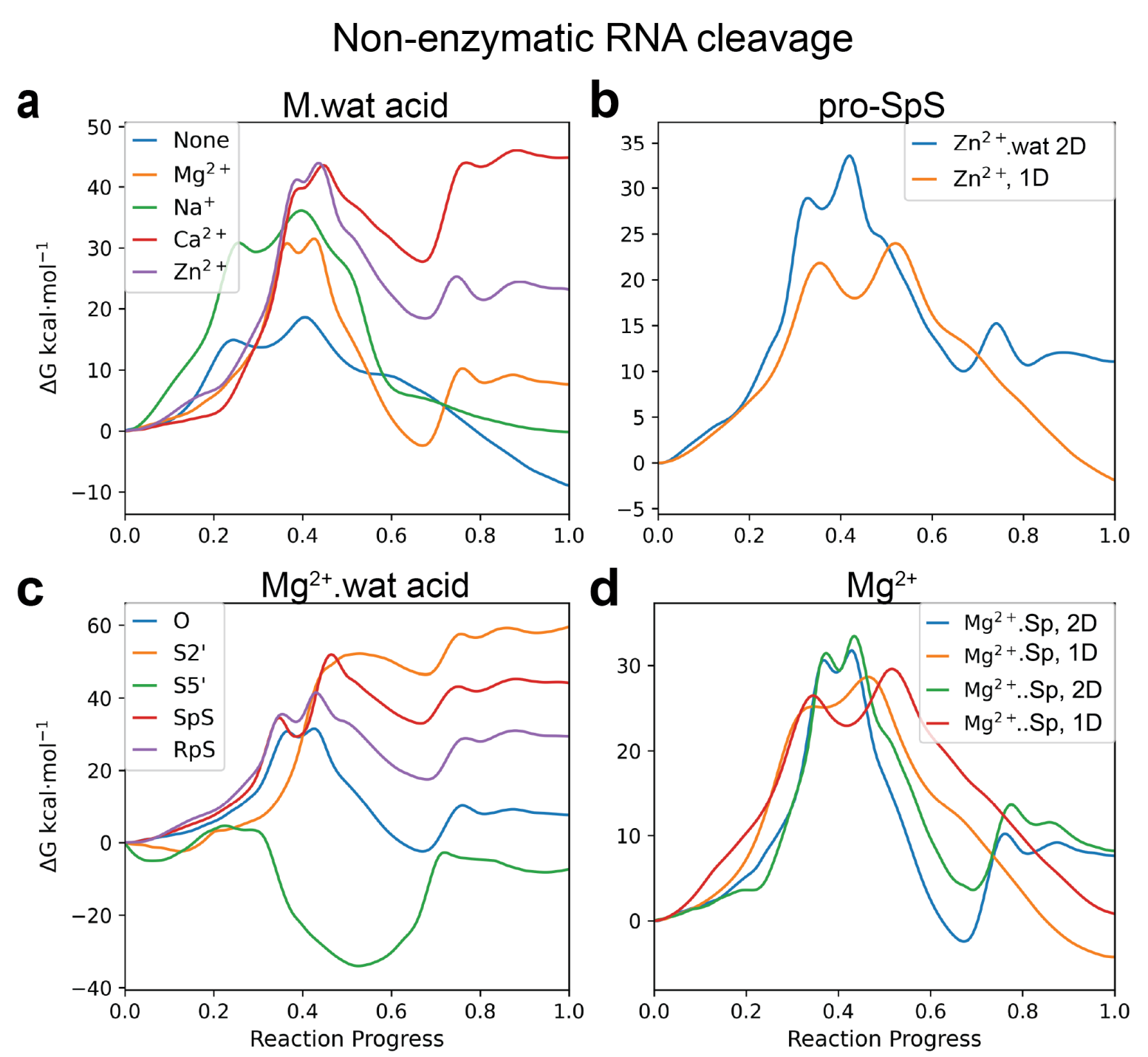

Figure S16: Free energy profiles for non-enzymatic reactions at the DFTB3 level. **(a)** Comparison of metal coordinated waters (M.wat) acting as an acid. **(b)** Comparison of the reaction with the pro-S_P oxygens substituted by sulfur (pro-SpS) with Zn^{2+} coordinated water acting as an acid (2D) and hydrogen bonding to the leaving group without transferring (1D). **(c)** Comparison of the reaction with Mg^{2+} coordinated water acting as an acid with oxygen substituted by sulfur. **(d)** Comparison of the reaction with Mg^{2+} coordinated water directly coordinating the pro-S_P (.) and indirectly coordinating (..), both donating a proton as an acid (2D) and donating a hydrogen bond (1D).

For the reactions of small nucleolytic ribozymes, the predominant concern arising from DFTB3

simulations is the sheer magnitude of the free energy barriers, especially for the HDVr and VSr reactions. Experimental estimates for these barriers with these ribozymes typically fall in the range of 16-20 kcal·mol^{-1}, with barriers of over 30 kcal·mol^{-1} predicting rates that would be difficult to even detect experimentally. For the HDVr reaction, which is sampled in 5 dimensions, 5 different initial path guesses yielded 5 distinct MFEP's, none of which would yield a detectable rate.

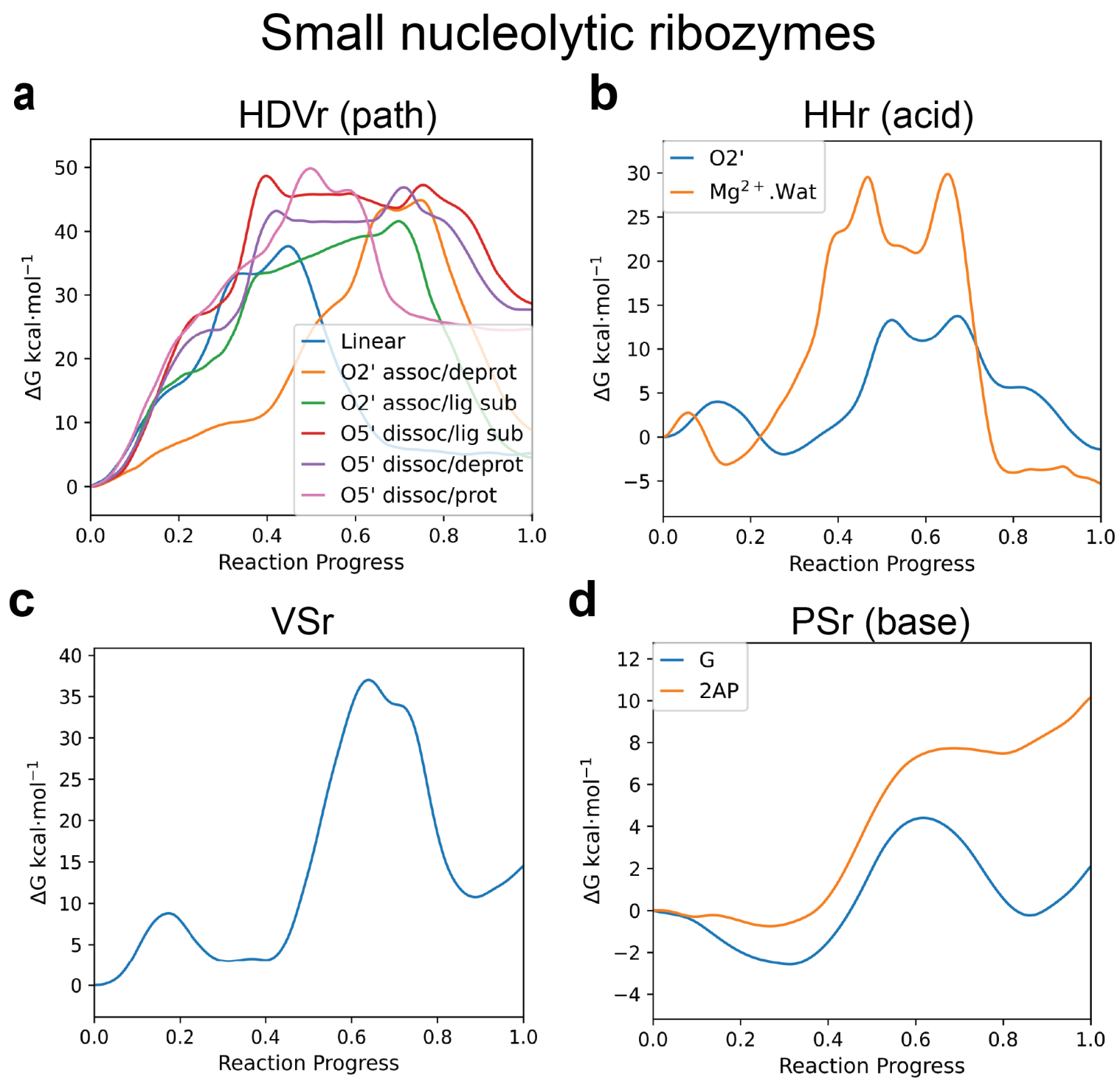

Figure S17: Free energy profiles for RNA cleaving ribozymes at the DFTB3 level. Titles indicate the component of the reaction being varied. **(a)** HDVr free energy profile initiated from different intial guesses. Labels indicate the steps that occurs first in the initial reaction path guess. **(b)** HHr free energy profiles with O2′ or Mg^{2+} coordinated water acting as the acid. **(c)** Free energy profile of the VSr reaction. **(d)** Free energy profile of the deprotonation reaction in PSr by guanine (G) and 2-aminopurine (2AP).

In the case of splicing by THr, all barriers well exceed experimental estimates of 16-17 kcal·mol^{-1}. The initial nucleophile deprotonation by hydroxide is shown to be endothermic. This hydroxide is indirectly coordinating Mg^{2+}, and the DFTB3 profiles would promote an interpretation that a water in this position has a pK_a downshifted below that of the nucleophile which directly coordinates Mg^{2+}.

The MTR1 reactions are relatively well described by DFTB3 compared to phosphoryl transfer reactions, although an over-stabilization of the intermediate does not comport with the relative

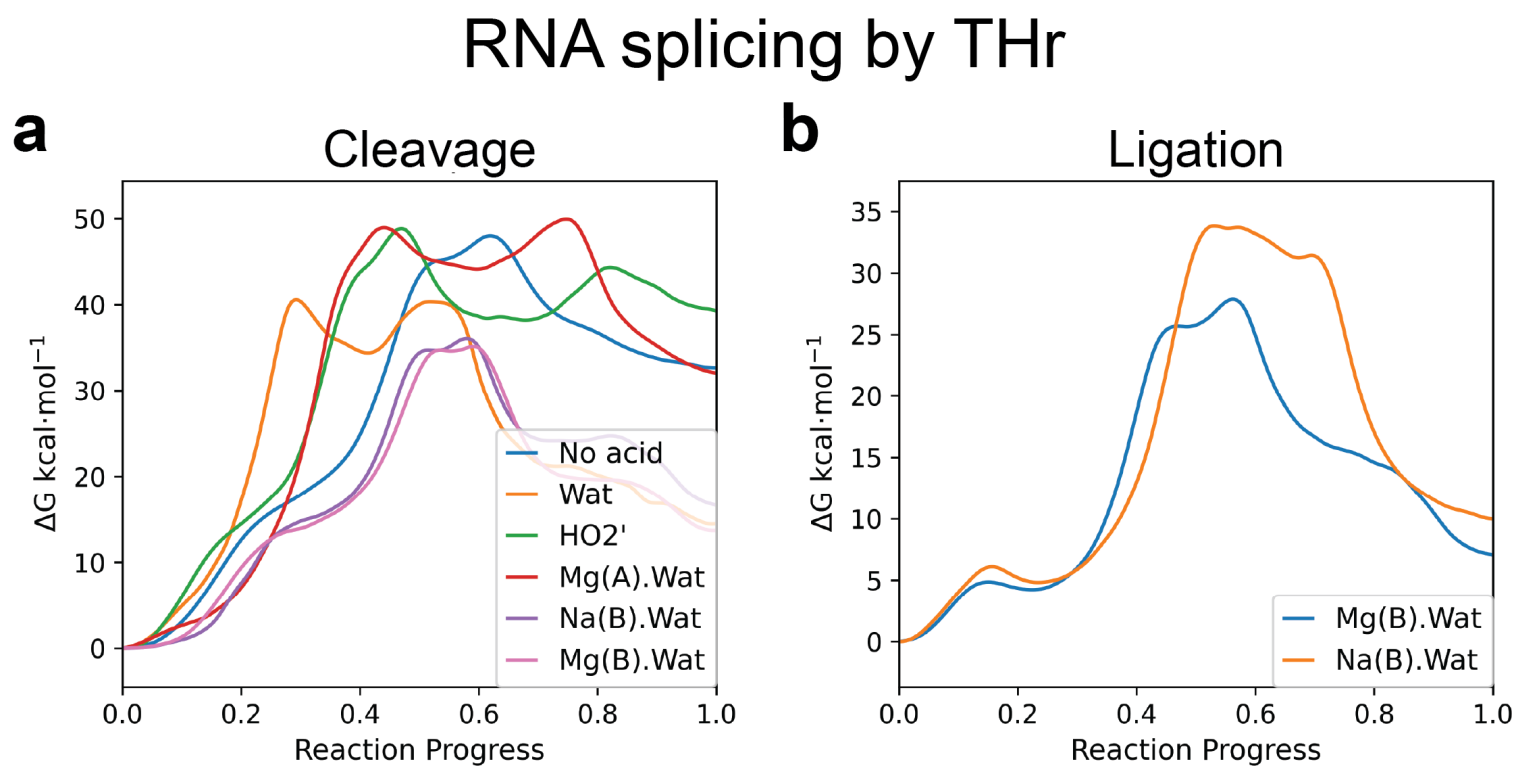

Figure S18: Free energy profiles of the THr cleavage and ligation reactions at the DFTB3 level. **(a)** Cleavage reactions where labels indicate the identity of the acid. **(b)** Ligation reactions where labels indicate a metal coordinated hydroxide deprotonating the nucleophile.

pK_a values of C and O6mG. In addition, the n7c modification is experimentally shown to lower the barrier by increasing nucleophilicity, but the barriers here are effectively equal. This trend is remedied with PBE0.

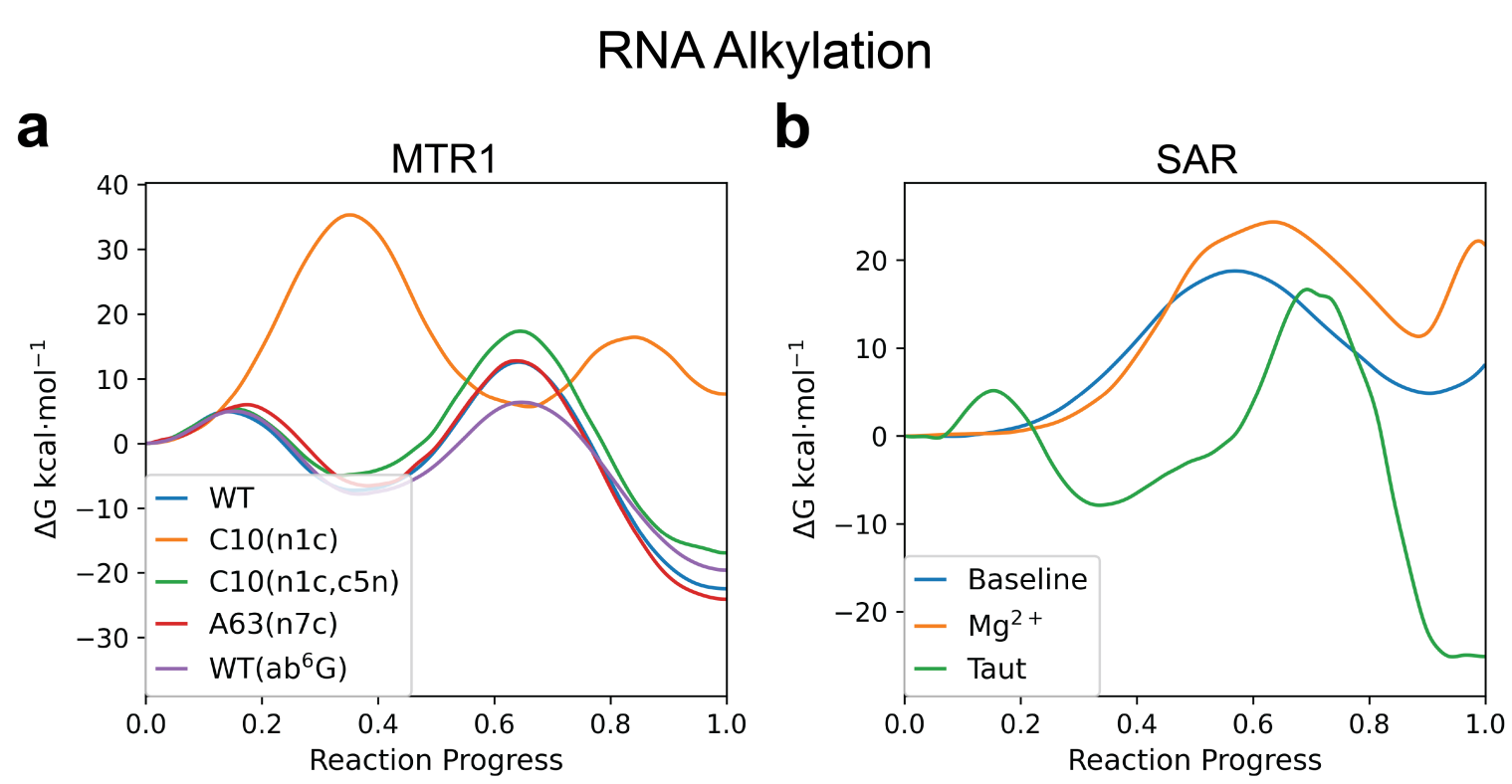

Figure S19: Free energy profiles for alkyl transfer reactions at the DFTB3 level. **(a)** Reactions of MTR1 where labels indicate isofunctional substitutions. The akyl donor is O6mG unless indicated as ab6G. **(b)** Reactions of SAR where the epoxide ring opening occurs without protonation (baseline), with protonation by Mg^{2+} coordinated water (Mg^{2+}), and by the O6 of a guanine tautomer (Taut).

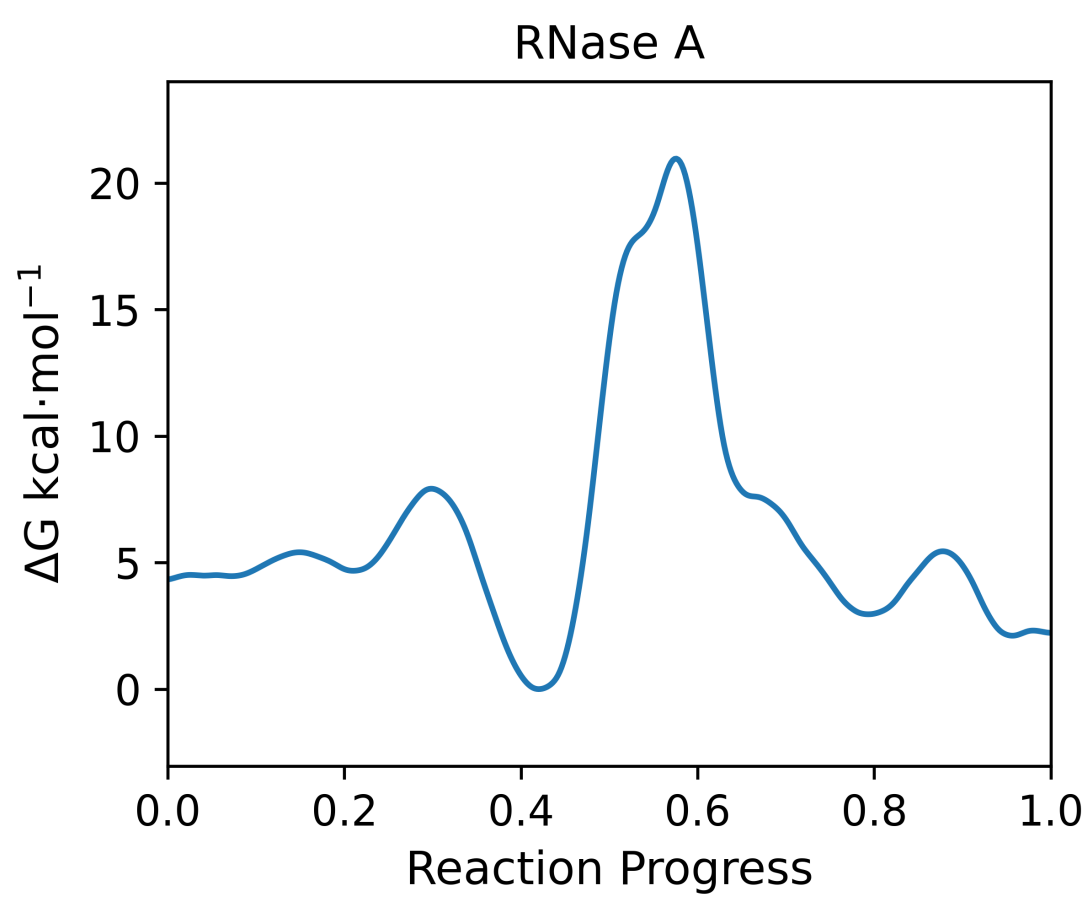

Figure S20: Free energy profile for the transphosphorylation reaction of RNase A at the DFTB3 level.

Validation of ΔMLP models on per-system training sets

We break the training errors down by system to gain further insight into system specific accuracy of the model. Figure S21 and Figure S22 show errors in predicted force and energy corrections, respectively. With the ΔDP model the RMSE in forces ranged from 0.53 to 0.81 kcal·mol^{-1}Å^{-1}, and for ΔMACE this ranged from 0.33 to 0.61. TWr, 8-17dz, and RNase A were the least represented systems, and their errors are predictably highest. For RNase A and 8-17dz, these scaffolds were also most dissimilar to the other systems in the training set, containing DNA and protein environments, indicating that further training could be performed as more data becomes available for these systems.

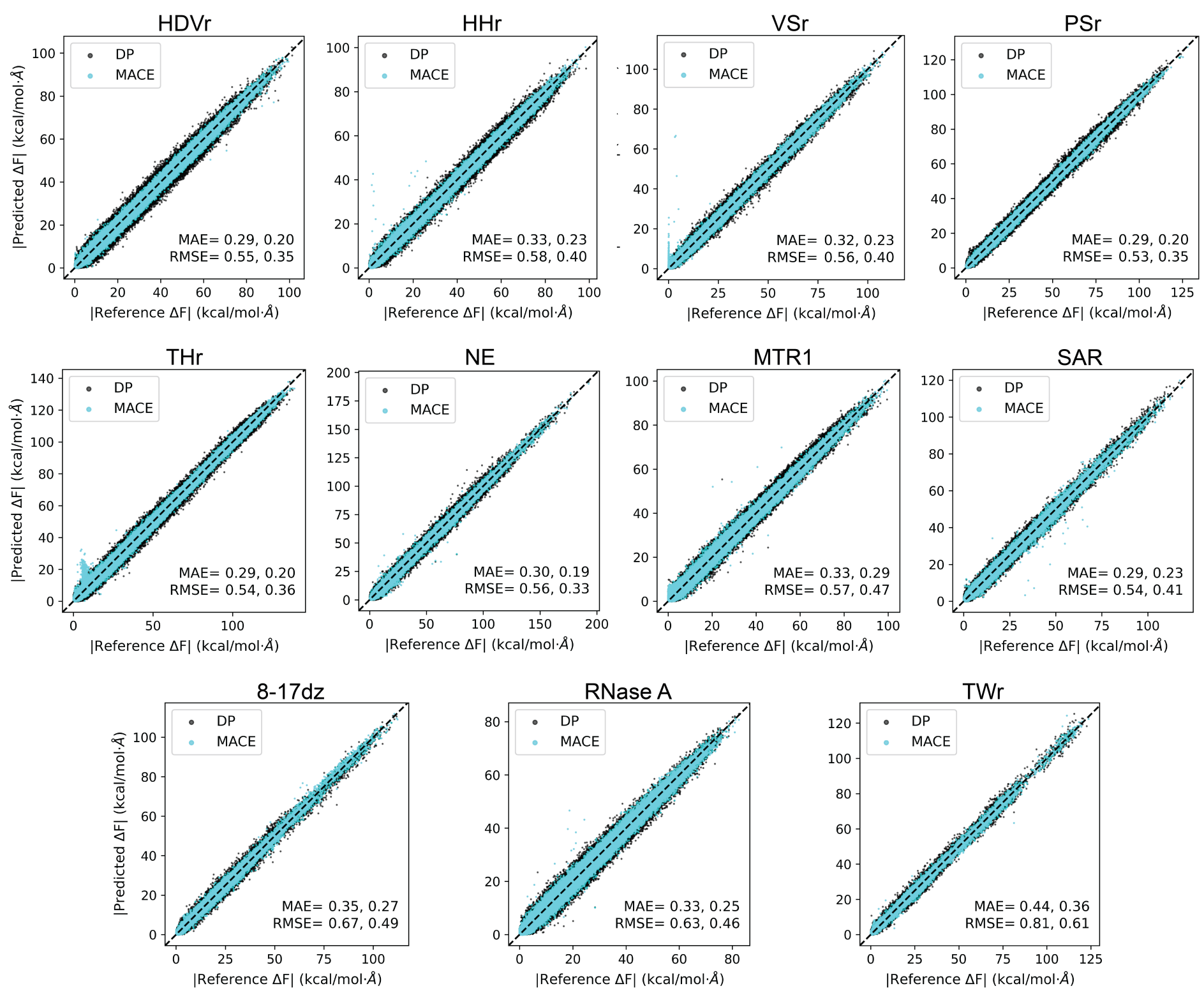

Figure S21: Per system errors in force corrections predicted by the ΔDP and ΔMACE models trained on the large data set. Titles indicate the system, and systems with multiple reactions have been combined. Errors are given as ΔDP,ΔMACE in kcal·mol^{-1}Å^{-1}.

The RMSE in predicted energy corrections with the ΔDP model ranged from 2.52 to 5.16 kcal·mol^{-1}, and ΔMACE ranged from 1.53 to to 5.14 kcal·mol^{-1}. The only two systems for which the ΔDP model outperforms the ΔMACE model are MTR1 and THr, both of which contain data for multiple reactions. Upon further inspection, the outlier data for MTR1 arises from the reaction

where the RNA substrate strand is mutated to DNA, and this RNA-DNA interaction is not highly represented in the data set. The THr outlier, although less dramatic than MTR1, arises from the only enzymatic case where the leaving group is not protonated by an acid and leaves as an oxyanion. Aside from these outliers, the systems with less available data generally yieled higher errors.

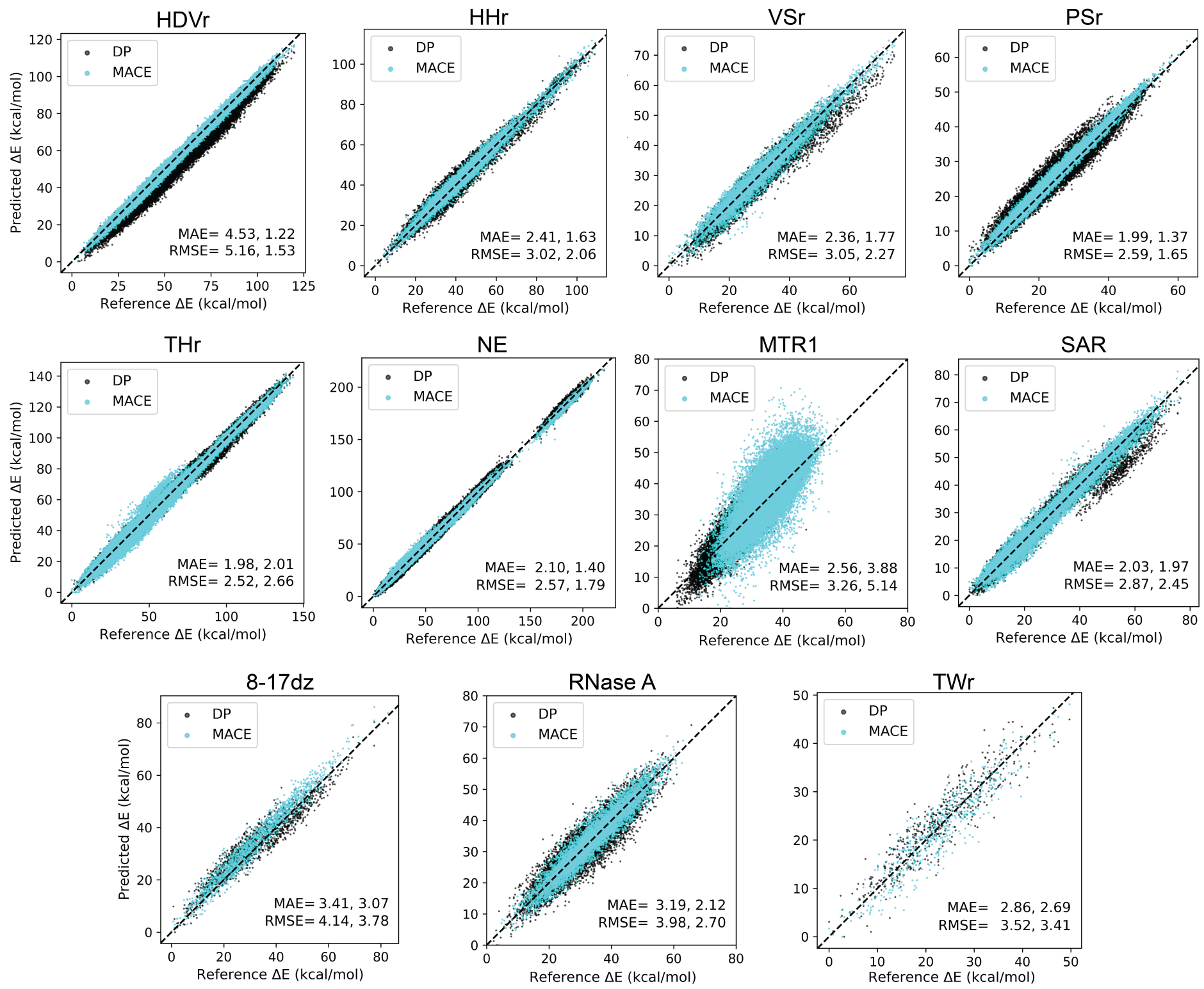

Figure S22: Per system errors in energy corrections predicted by the ΔDP and ΔMACE models trained on the large data set. Titles indicate the system, and systems with multiple reactions have been combined. Errors are given as ΔDP,ΔMACE in kcal·mol^{-1}.

Table S2: Data pruning by pool-based active learning on small and large data sets

Model	Data set	Total samples	Unique	Iterations	Accurate	Trained
DP	S	139,000	95,580	10	13,168	125,832
DP	L	369,000	233,125	15	184,938	184,062
MACE	S	139,000	95,580	9	39,191	99,809
MACE	L	369,000	233,125	16	180,857	188,143

Total samples refers to the total numbers of trajectory frames in the data set. Unique refers to frames with unique QM and MM atomic compositions. Non-unique frames can have the same number of each atom but with different atomic positions. Accurate refers to the cumulative number of frames over the training iterations pruned from the data set based on model deviation. Trained refers to the cumulative number of frames explicitly trained on (Total samples - Accurate).

Evaluation of transferability to experimentally relevant test sets

Next we evaluate in the detail the transferability of the trained models to the test set reactions outlined in Figure S14. As shown in Figure S23, the ΔDP and ΔMACE models trained on the small data set consistently outperform DFTB3, and their force errors are relatively similar to each other. This comports with the results of the training set analysis from the small data set. As shown in Figure S24, the ΔMACE model significantly outpaces the ΔDP model when trained on the large data set. The transferability tests with the ΔMACE-L model again reflect the results of the training set, as increasing the number of channels does not provide any marked transferability benefits. These results are shown in Figure S25.

A clear advantage in transferability comes when comparing the specific reaction potentials (SRP) trained from scratch and with refinement from the ΔMACE model (R-SRP). As shown in Figure S26, particularly for the QM and MM modifications, the refined potential is better able to handle modifications, suggesting it has retained some knowledge from seeing similar reaction components in other contexts. The SRP trained from scratch still performs better than DFTB3 and generally extrapolates well to high energy paths, but large perturbations to the QM and MM region will likely yield higher errors and necessitate further training for high accuracy. In general, the perturbation that caused higher errors across all potentials was the C75ΨisoC modification in HDVr. This swaps the C5 for N and the N1 for C, creating a new hydrogen bond donor at the N5 position. This in turn yields a new hydrogen bond between the the QM cytosine and an MM phosphate. It appears that modification at the QM/MM interface that induces interactions not previously seen by the model may lead to higher errors.

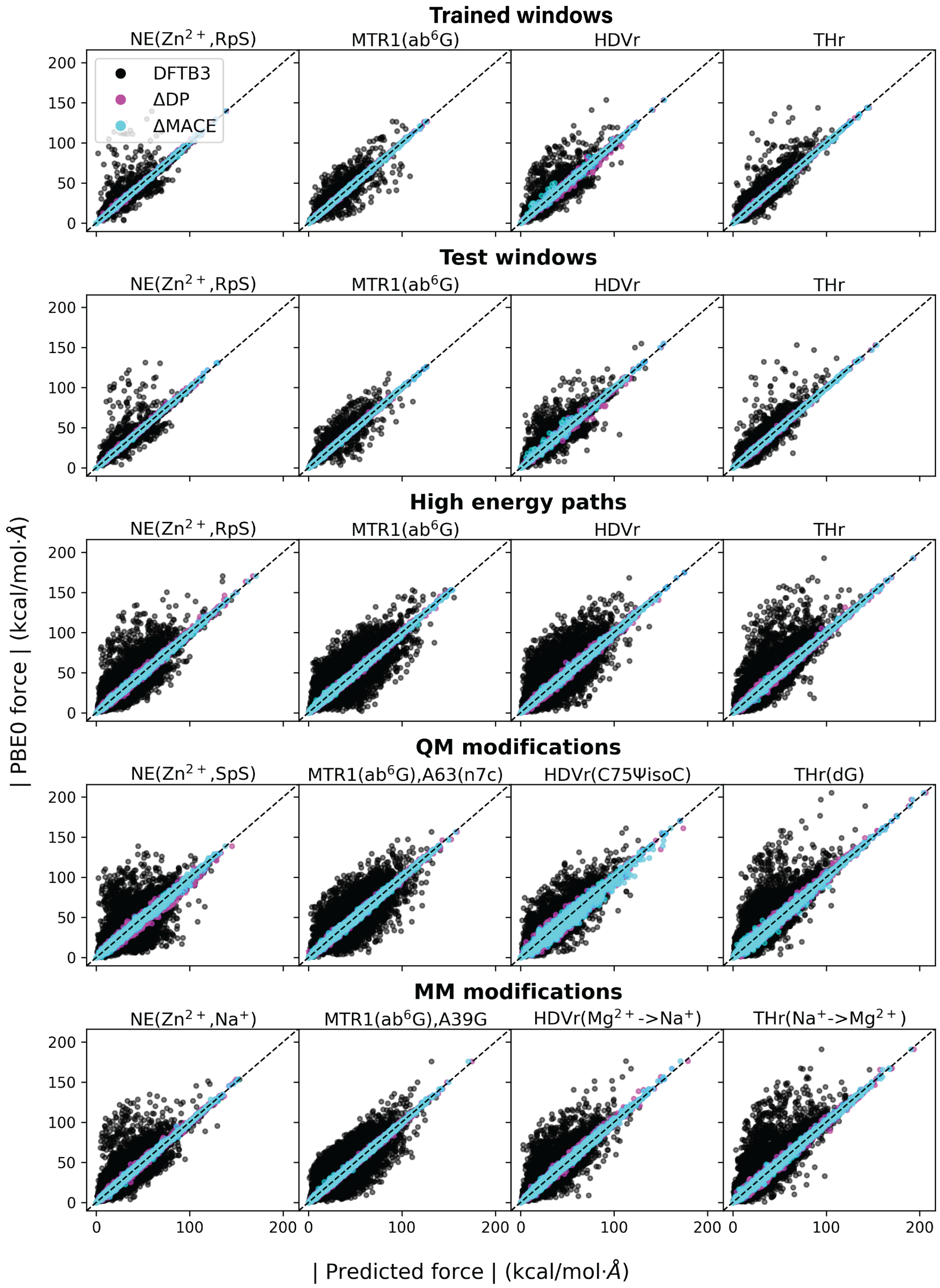

Figure S23: Errors in absolute forces predicted by DFTB3, ΔDP, and ΔMACE models trained on the small data set compared to PBE0 for non-enzymatic, MTR1, HDVr, and THr reactions for the umbrella windows in the training set, and for test sets including non-trained windows, high energy pathways, QM modifications and MM modifications.

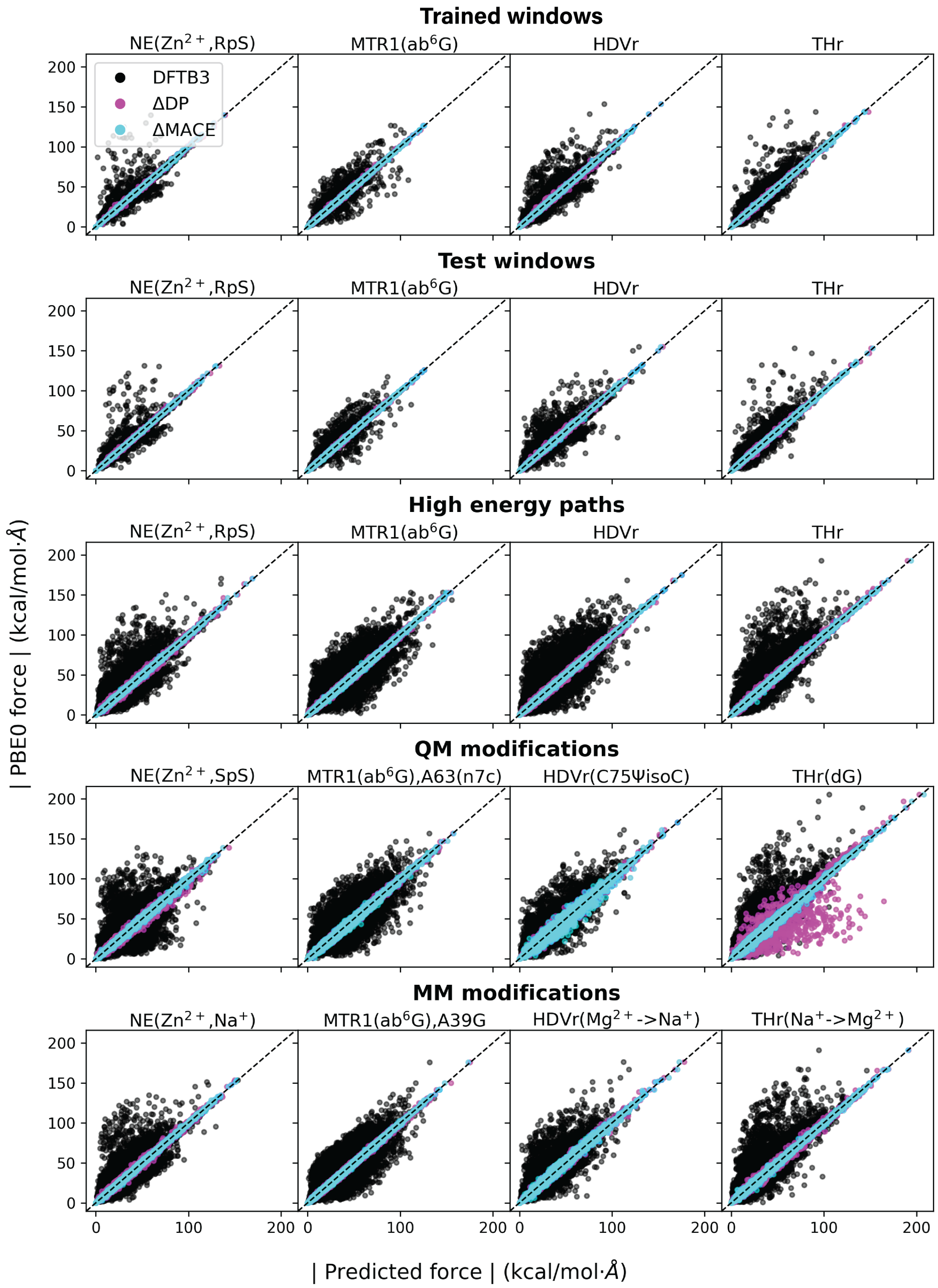

Figure S24: Errors in absolute forces predicted by DFTB3, ΔDP, and ΔMACE models trained on the large data set compared to PBE0 for non-enzymatic, MTR1, HDVr, and THr reactions for the umbrella windows in the training set, and for test sets including non-trained windows, high energy pathways, QM modifications and MM modifications.

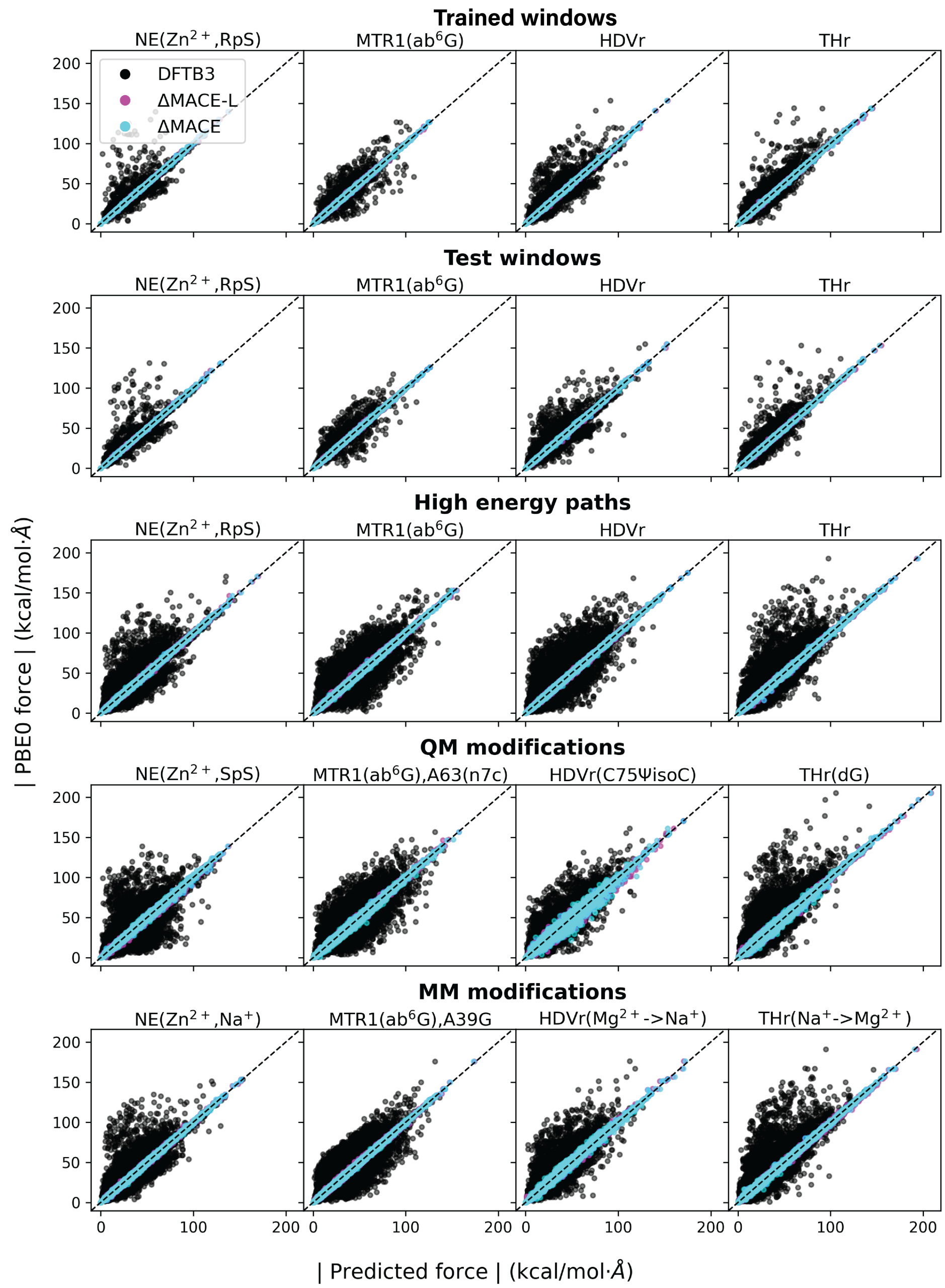

Figure S25: Errors in absolute forces predicted by DFTB3, ΔMACE, and ΔMACE-L models trained on the large data set compared to PBE0 for non-enzymatic, MTR1, HDVr, and THr reactions for the umbrella windows in the training set, and for test sets including non-trained windows, high energy pathways, QM modifications and MM modifications. ΔMACE refers to the default parameters containing 128 channels, and ΔMACE-L indicates increases the channels to 256.

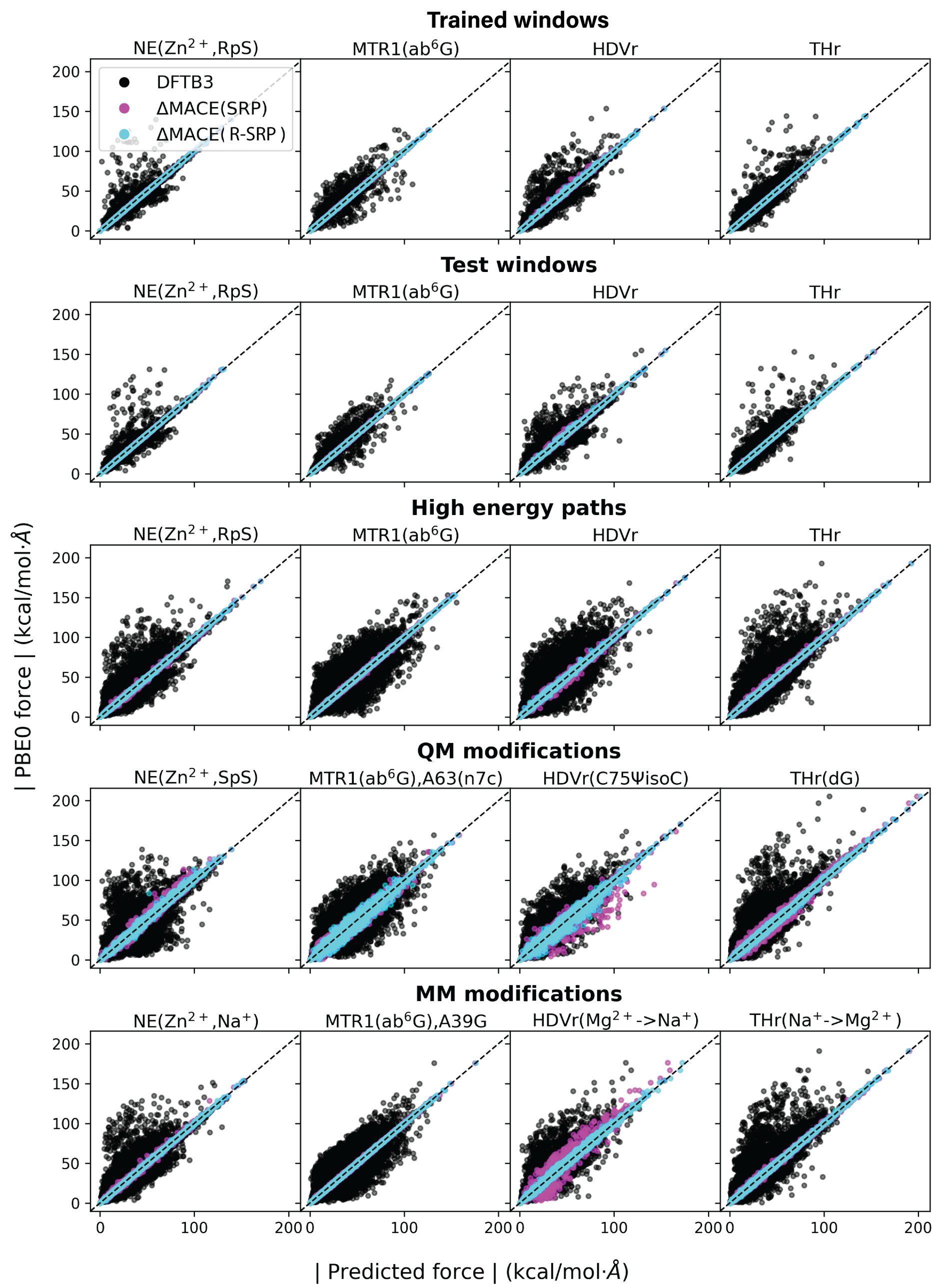

Figure S26: Errors in absolute forces predicted by DFTB3, ΔMACE(SRP), and ΔMACE(R-SRP) models trained on the large data set compared to PBE0 for non-enzymatic, MTR1, HDVr, and THr reactions for the umbrella windows in the training set, and for test sets including non-trained windows, high energy pathways, QM modifications and MM modifications. SRP refers to a specific reaction potential trained from scratch with MACE, and R-SRP indicates a specific reaction potential initiated from the foundational ΔMACE model.

Additional analysis of the VSr case study reaction

In previous sections we referred to the 2 dimensional reaction of VSr, which departed from an activated nucleophile. In this section we expand to a 3 dimensional reaction that involves nucleophile deprotonation, previously too expensive to sample in PBE0. A schematic of this reaction is given in Figure S27, and the resulting free energy barriers achieved with different potentials are given in Table S1. Although GFN2-xTB appears to perform well in 2 dimensions, allowing for reaction path-finding in 3 dimensions yields an outlier pathway in which nucleophile deprotonation and phosphoryl transfer are more concerted rather than step-wise. This yields a very low barrier of 6.15 kcal·mol^{-1}, well below experiment. Between the VSr and the HDVr reactions, GFN2-xTB appears to yield tighter transition states that favor mechanisms where negative charge is more diffuse, in this case avoiding a seemingly stable intermediate state where the nucleophile is negatively charged.

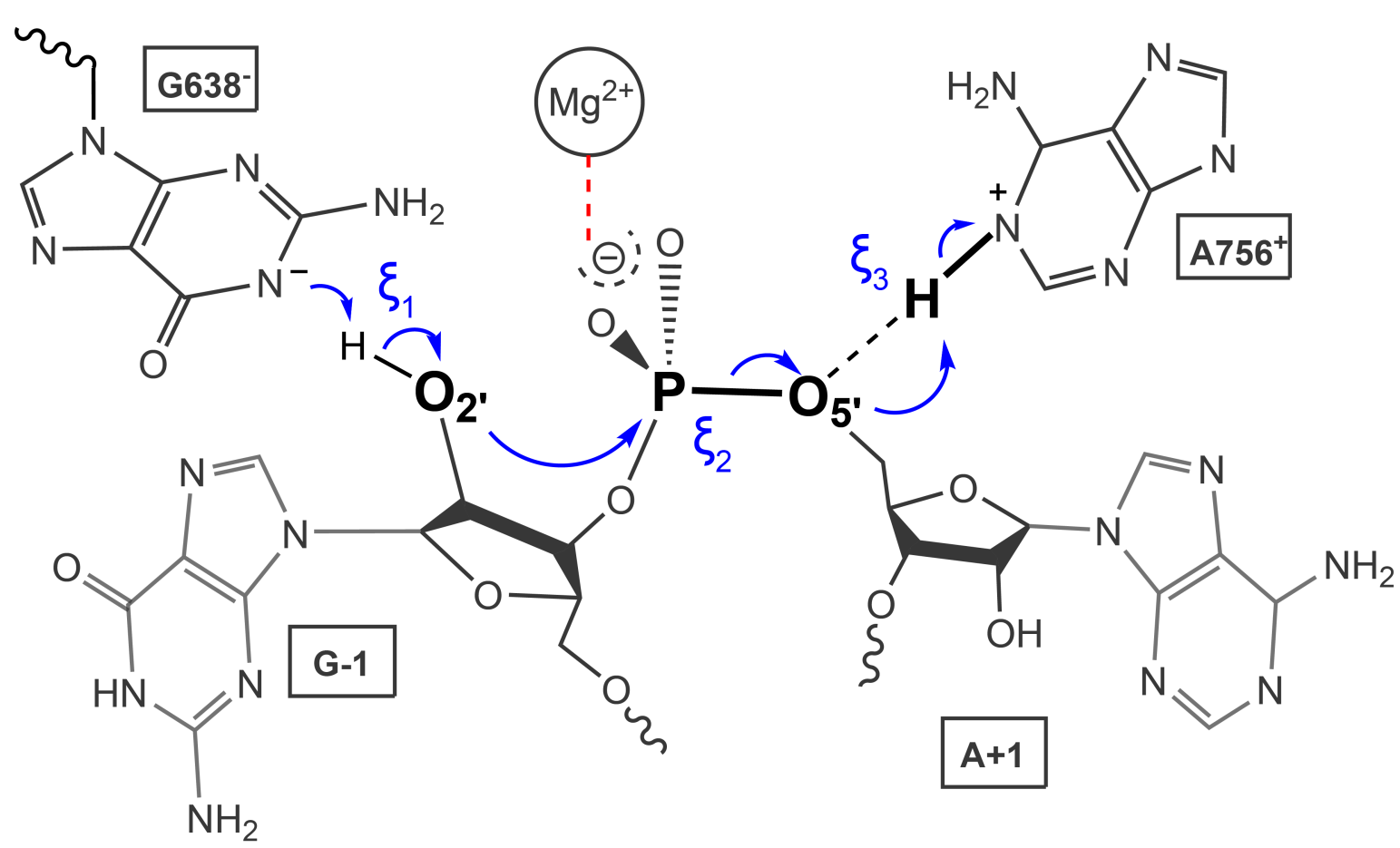

Figure S27: Schematic of the VSr reaction sampled in 3 dimensions. Reaction coordinates correspond to nucleophile deprotonation, phosphoryl transfer, and leaving group protonation, respectively.

Although the ΔMACE-AL refined model is more accurate than the foundational ΔMACE model, the MFEPs are very similar, as evidenced by the reaction coordinates at the transition state. This means that a more relevant set of structures can be fed to the active learning procedure for refinement, rather than supplying structures obtained with a semiempirical model far from the target MFEP. This again cautions users against the practice of simply obtaining a semiempirical level path and performing *ab initio* simulations on it, as this may not be a good representation of the *ab initio* MFEP.

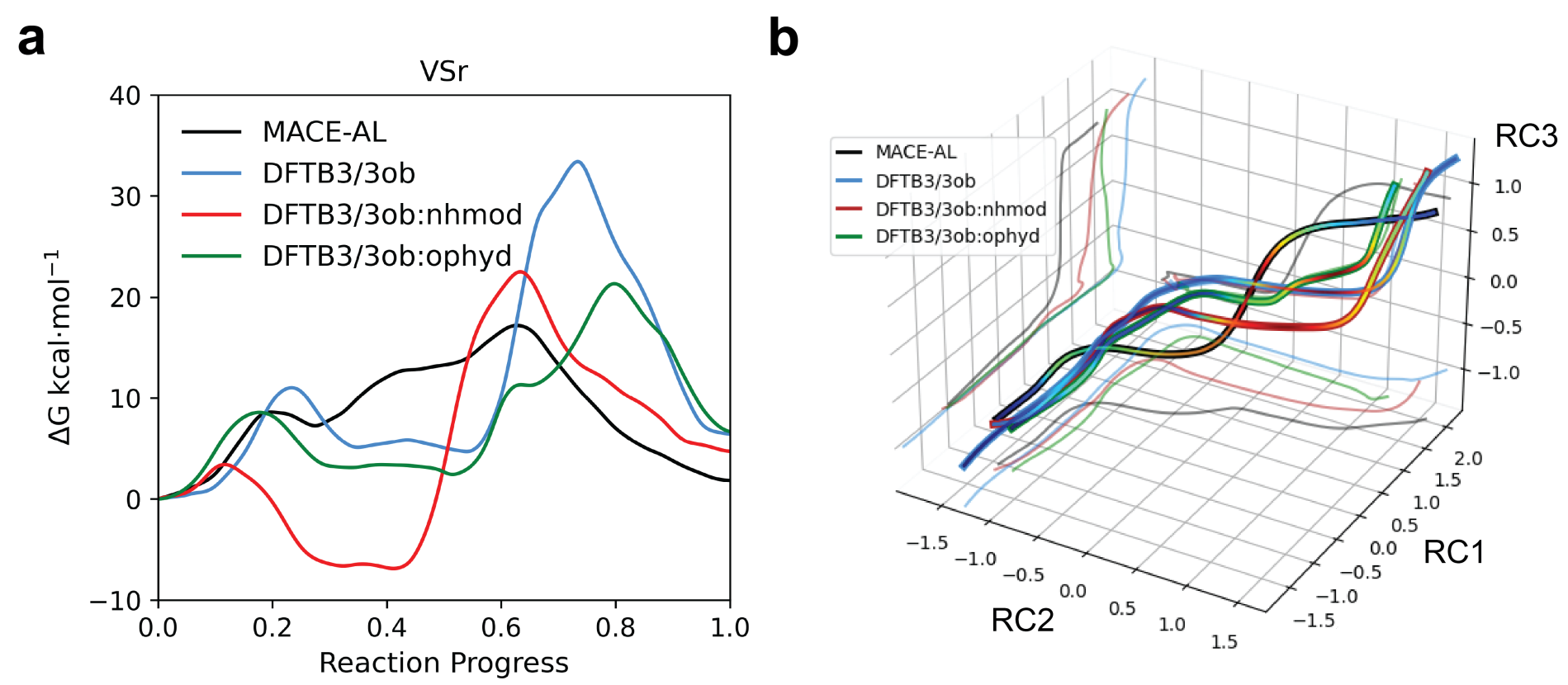

Figure S28: Free energy profiles **(a)** of the VSr reaction in 3 dimensions comparing DFTB3/3ob specific reaction parameterizations, nhmod and ophyd, and associated minimum free energy paths **(b)**.

Table S3: Free energy barriers for reactions of VSr sampled in 3 dimensions

	(kcal·mol^{-1})		(Å)		(kcal·mol^{-1})
Potential	$\Delta G^{\ddagger}$	$\xi_1^{\ddagger}$	$\xi_2^{\ddagger}$	$\xi_3^{\ddagger}$	ΔG_{rxn}
AM1/d	35.66	0.38	1.45	-0.19	13.48
DFTB3/3ob	33.41	0.35	2.36	-0.57	6.43
GFN2-xTB	6.15	-0.70	-0.20	-0.85	-8.37
DFTB3/3ob:nhmod	22.50	0.31	1.55	-0.60	4.71
DFTB3/3ob:ophyd	21.29	0.53	2.12	-0.22	6.68
ΔDP	16.86	-0.80	0.58	-0.81	-0.73
ΔMACE	15.11	0.19	1.07	-0.02	-3.67
ΔMACE-AL	17.19	0.16	1.15	0.00	1.84

Free energy barriers ($\Delta G^{\ddagger}$) and associated reaction coordinates (ξ) as defined in the "System specific preparation" section of the Supplementary Information. G_{rxn} refers to the free energy of reaction. Free energy profiles correspond to those shown in Figure 4.

Specific components of workflow leveraging the ΔNAzyme model

The following elements accompany the flowchart provided in Figure 4d of the main text. The components are either functions, data, or models.

Functions

- Sander (QM/MM MD): The AMBER sander engine is used to run QM/MM molecular dynamics simulations.

- Sander (QM/MM snglpt): The AMBER sander engine is used to perform single point calculations on a fixed trajectory (achieved by setting imin=6 and nstlim=0).

- DP AMBER: The `dp amber corr` functionality from DeePMD-kit is used to extract data from AMBER output files (trajectories, forces, and energies) and store it in hdf5 file format.

- DP MODEL-DEVI: The `dp model-devi` function within DeePMD-kit is used to compute the deviation in forces predicted by a committee of models to estimate model uncertainty.

- DP TRAIN: The `dp train` function within DeePMD-kit is used to train ΔMLP models.

Data

- Structures, $q(x)$: a system state defined by the potential energy function evaluated with a given set of system parameters at coordinates, x. This equates to the .parm7 and .rst7 parameter and topology files in AMBER.

- Trajectories

    - $\{q(x)\}_i$: a set of system states, or frames, resulting from a simulation using Hamiltonian i.

    - $\{q(x)\}_i^{jk}$: a set of frames generated with i and evaluated for forces and energies at Hamiltonians j and k.

- Labeled systems

- $\{R; \Delta F; \Delta E\}$: a set of numpy objects storing the environment (R) within r_{cut} of the QM region and the associated difference in forces (ΔF) and energies (ΔE) between Hamiltonians j and k extracted from a trajectory.
- $\{R; \Delta F; \Delta E\} \in C$: The labeled systems designated as candidates (C) for training based on model deviation criteria. This comprises the pool.

Models

- Model parameters, $\{\Theta_l\}_4$: The set of parameters defining a set of 4 models after training iteration i using training approach l.
- Optimized model parameters, $\{\Theta_l\}_4^*$: The final set of optimal ($*$) model defining parameters.
  - l=Δ_{NAzyme}: The foundational model trained in this work.
  - l=P-AL: The pool-based active learning approach in which pre-labeled data constitutes a pool from which data is iteratively drawn from and removed as accuracy is achieved. This is performed using the `dpgen simplify` function.
  - l=O-AL: The on-the-fly active learning approach in which data is generated on-the-fly at each training iteration by performing simulations with the model trained in the previous iteration. This is performed using the `dpgen run` function.

Novel reaction of the SAMURI ribozyme

The methyl transfer reaction catalyzed by the SAMURI ribozyme presents novel chemistry not present in the NAzyme data set. The detailed reaction mechanism is shown in Figure S29. While methyl transfer to a target adenine is seen in the MTR1 reaction, the leaving group is oxygen rather than sulfur, and the adenine is methylated at the N1 site rather than N3. The SAM ligand is structurally and electrostatically novel, and presents an carboxylate-Mg^{2+} coordination interaction not present in the training set.

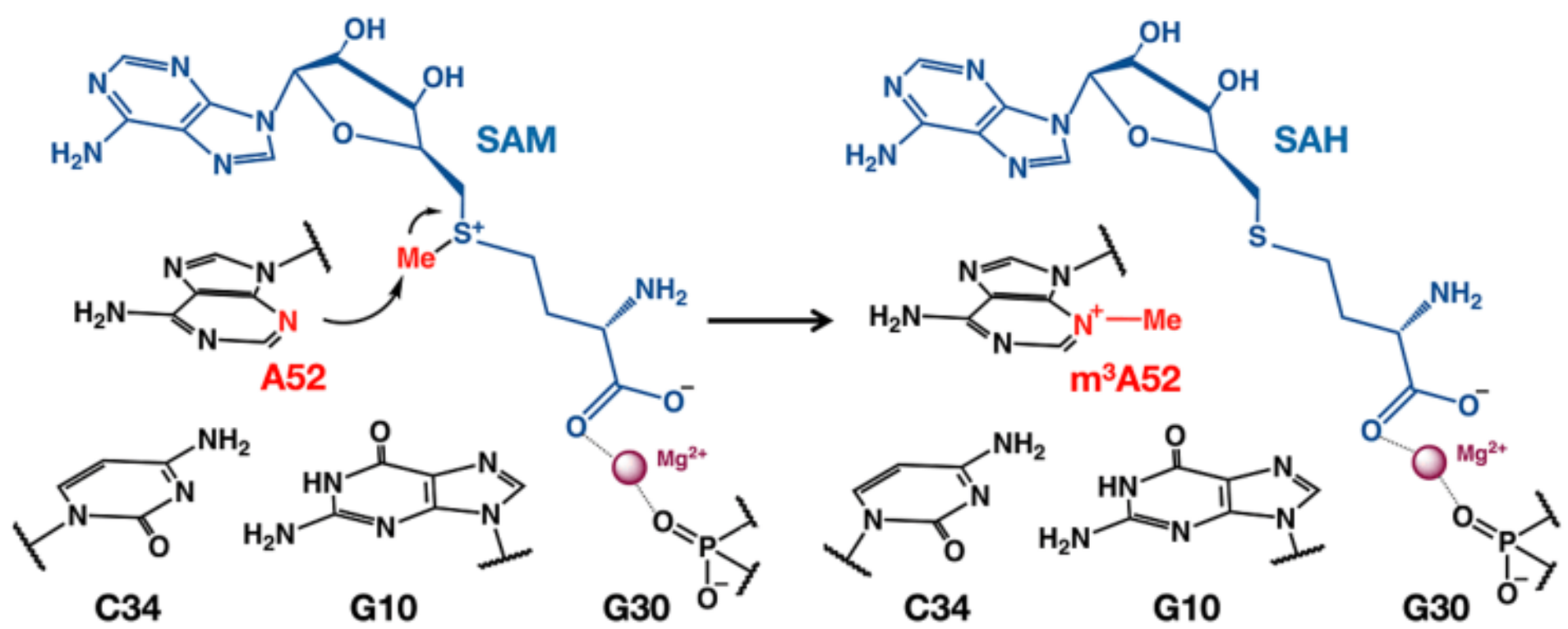

Figure S29: Site specific methylation of a target adenine (A52:N3) from the SAM cofactor (blue) catalyzed by the SAMURI ribozyme (black).

Additional validation of PMFs

The umbrella integration approach was used to correct ΔMACE potentials of mean force (PMF) to the DFTB3 level to demonstrate that short simulations with large force constants are sufficient to observe differences between potentials if they in fact deviate. As shown in Figure S30, the DFTB3 PMFs along the ΔMACE paths differ significantly as expected.

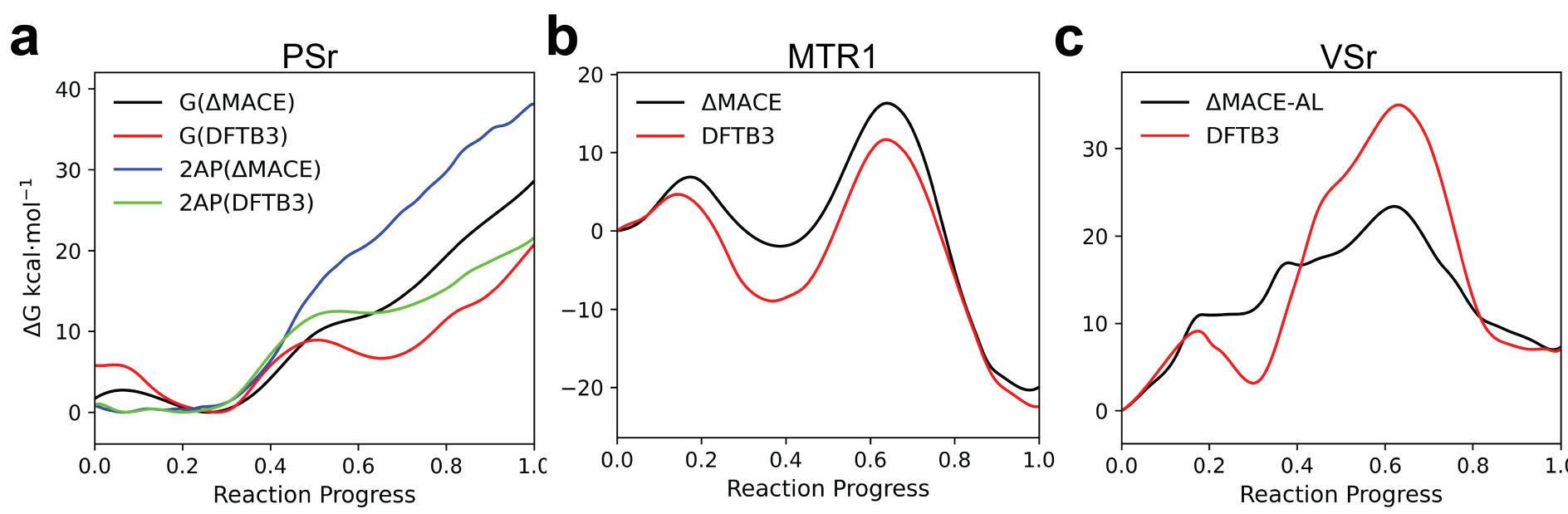

Figure S30: PMF from the ΔMACE model compared to the DFTB3 level. PMF for **(a)** Guanine (G) and 2-aminopurine (2AP) deprotonation reactions in the Pistol ribozyme, **(b)** 2D methyl transfer reaction in the methyl transferase ribozyme, and **(c)** 3D phosphoryl transfer in Varkud satellite ribozyme.

The free energy barriers obtained from umbrella integration in Figure S30 and Figure 5 of the main text are provided in Table S4. In addition, an RMSD metric is provided to quantify the total difference between reference and target level PMFs, where the reference has an RMSD of zero. RMSD was computed as:

$$\mathrm{RMSD} = \sqrt{\frac{1}{N}\sum_{i=1}^{N}([\Delta G_t(x_i) - \Delta G_t(x_0)] - [\Delta G_r(x_i) - \Delta G_r(x_0)])^2} \tag{34}$$

where x is the reaction progress variable that ranges from 0 to 1, r refers to the reference potential, and t to the target.

The umbrella integration (UI) approach was taken to analyze the short sampling that is affordable to achieve at the *ab initio* level. To further justify the use of UI, we compare free energy profiles analyzed by UI, Multi-state Bennett Acceptance Ratio (MBAR), and Weighted Histogram Analysis Method (WHAM) in Figure S31. In order to achieve coherent profiles with MBAR and WHAM, 25 ps of sampling was analyzed with each method. The MBAR and UI approaches agree extremely well, with WHAM differing by up to 2 kcal·mol^{-1}.

Table S4: Free energy barriers and PMF validation for reactions of PSr, MTR1, and VSr

		kcal·mol^{-1}	
System	Potential	ΔG‡	RMSD
PSr(G)	ΔMACE	28.58	0.00
	PBE0	28.34	0.78
	DFTB3	20.74	8.09
PSr(2AP)	ΔMACE	38.13	0.00
	PBE0	35.96	2.41
	DFTB3	21.59	8.94
MTR1	ΔMACE	18.30	0.00
	PBE0	19.68	0.58
	DFTB3	20.63	4.24
VSr	ΔMACE-AL	23.36	0.00
	ΔMACE	25.84	2.51
	PBE0	23.04	0.35
	DFTB3	34.99	6.39

Free energy barriers ($\Delta G^{\ddagger}$) are computed from umbrella integration. RMSD was computed according to Equation 34.

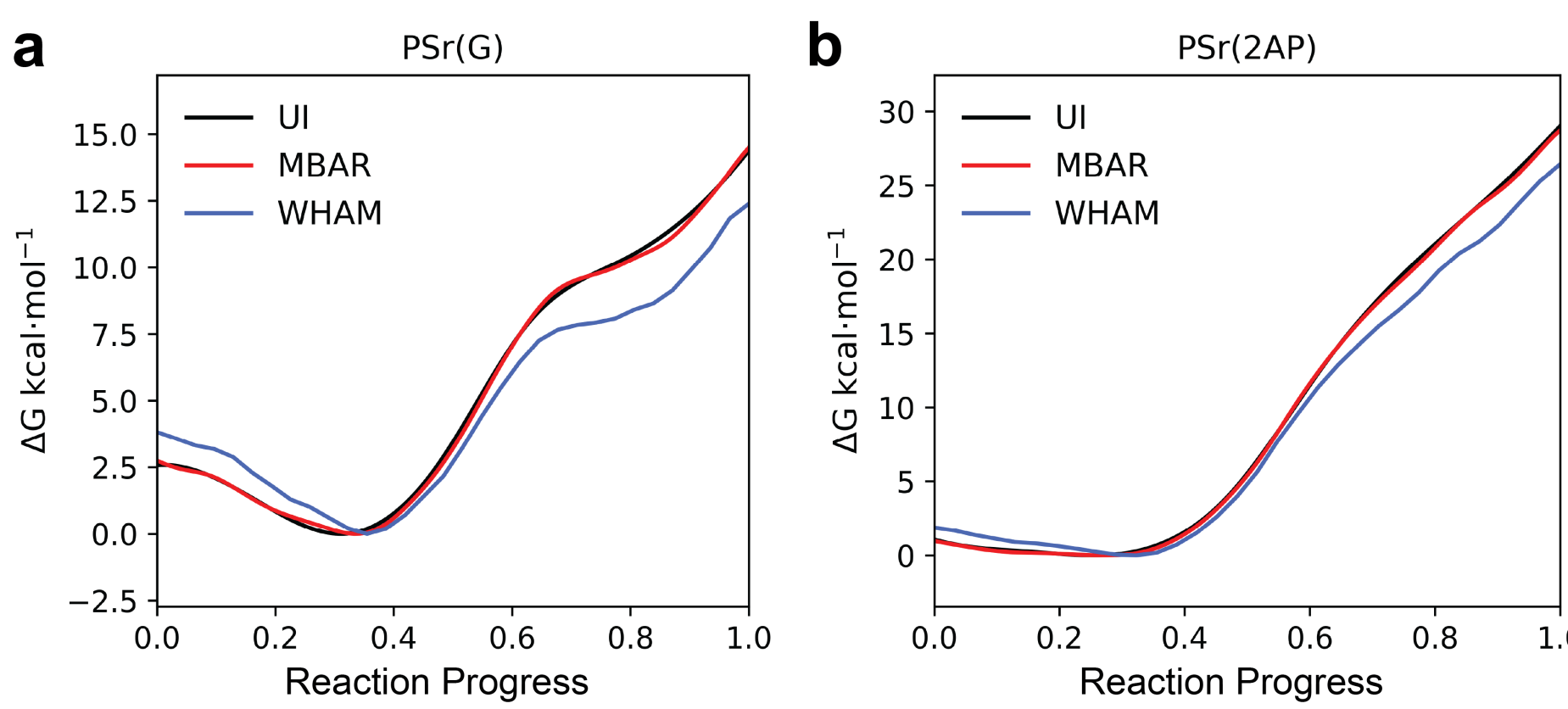

Figure S31: 1-dimensional ΔMACE free energy profiles from 25 ps of the PSr deprotonation reaction with **(a)** general base G and **(b)** 2AP analyzed by UI, MBAR, and WHAM.

Simulation timing comparison

We use the test set systems to demonstrate the speed-up afforded by the ΔMLP models. There is a small overhead of 33% or less for performing the ΔMLP evaluation on GPUs; however ΔMLP simulations were between 400 and 1000 times faster than PBE0. The number of CPUs used for PBE0 benchmarking was systematically increased until the electron repulsion integrals could be stored in memory. The ΔDP and ΔMACE timings were comparable on GPUs. Overall, the speed-up afforded by the ΔNAzyme models is dramatic when GPUs are available.

Table S5: Timings comparison for simulations of test set systems with DFTB3, ΔNAzyme models, and PBE0.

	Natoms		ps/day/CPU core		ps/day/GPU		Ncores
System	Total	QM	DFTB3	PBE0	ΔDP	ΔMACE	PBE0
NE	6056	40	390	0.32	330	340	32
MTR1	75383	62	50	0.10	40	40	32
HDVr	71237	93	40	0.03	30	30	64
THr	281595	93	10	0.01	10	10	128

Timings are given for a single umbrella window. Natoms refers to the number of atoms in the entire system (total) and the QM region (QM). Ncores refers to the number of CPU cores used to obtain PBE0 timings. All DFTB3 timings were obtained with 1 CPU core. All GPU timings used 1 CPU core for the underlying DFTB3 evaluation with the GPU utilized for the ΔMLP evaluation. 2.4 GHz Intel Xeon E5-2680 v4 CPUs and GeForce RTX 2080 Ti GPUs were used for benchmarking.

References

(S1) Chen, H.; Piccirilli, J. A.; Harris, M. E.; York, D. M. Effect of Zn^{2+} binding and enzyme active site on the transition state for RNA 2'-O-transphosphorylation interpreted through kinetic isotope effects. *Biochim. Biophys. Acta, Proteins Proteomics* **2015**, *1854*, 1795–1800.

(S2) Zhang, S.; Gu, H.; Chen, H.; Strong, E.; Ollie, E. W.; Kellerman, D.; Liang, D.; Miyagi, M.; Anderson, V. E.; Piccirilli, J. A. et al. Isotope effect analyses provide evidence for an altered transition state for RNA 2'-O-transphosphorylation catalyzed by Zn^{2+}. *Chem. Commun.* **2016**, *52*, 4462–4465.

(S3) Giese, T. J.; Zeng, J.; Ekesan, Ş.; York, D. M. Combined QM/MM, Machine Learning Path Integral Approach to Compute Free Energy Profiles and Kinetic Isotope Effects in RNA Cleavage Reactions. *J. Chem. Theory Comput.* **2022**, *18*, 4304–4317.

(S4) Weissman, B.; Ekesan, Ş.; Lin, H.-C.; Gardezi, S.; Li, N.-S.; Giese, T. J.; McCarthy, E.; Harris, M. E.; York, D. M.; Piccirilli, J. A. Dissociative Transition State in Hepatitis Delta Virus Ribozyme Catalysis. *J. Am. Chem. Soc.* **2023**, *145*, 2830–2839.

(S5) Lee, T.-S.; Silva-Lopez, C.; Martick, M.; Scott, W. G.; York, D. M. Insight into the role of Mg^{2+} in hammerhead ribozyme catalysis from x-ray crystallography and molecular dynamics simulation. *J. Chem. Theory Comput.* **2007**, *3*, 325–327.

(S6) Lee, T.-S.; Silva Lopez, C.; Giambaşu, G. M.; Martick, M.; Scott, W. G.; York, D. M. Role of Mg^{2+} in hammerhead ribozyme catalysis from molecular simulation. *J. Am. Chem. Soc.* **2008**, *130*, 3053–3064.

(S7) Lee, T.-S.; Giambaşu, G. M.; Sosa, C. P.; Martick, M.; Scott, W. G.; York, D. M. Threshold Occupancy and Specific Cation Binding Modes in the Hammerhead Ribozyme Active Site are Required for Active Conformation. *J. Mol. Biol.* **2009**, *388*, 195–206.

(S8) Lee, T.-S.; York, D. M. Computational mutagenesis studies of hammerhead ribozyme catalysis. *J. Am. Chem. Soc.* **2010**, *132*, 13505–13518.

(S9) Wong, K.-Y.; Lee, T.-S.; York, D. M. Active participation of the Mg^{2+} ion in the reaction coordinate of RNA self-cleavage catalyzed by the hammerhead ribozyme. *J. Chem. Theory Comput.* **2011**, *7*, 1–3.

(S10) Chen, H.; Giese, T. J.; Golden, B. L.; York, D. M. Divalent Metal Ion Activation of a Guanine General Base in the Hammerhead Ribozyme: Insights from Molecular Simulations. *Biochemistry* **2017**, *56*, 2985–2994.

(S11) Ganguly, A.; Weissman, B. P.; Piccirilli, J. A.; York, D. M. Evidence for a Catalytic Strategy to Promote Nucleophile Activation in Metal-Dependent RNA-Cleaving Ribozymes and 8-17 DNAzyme. *ACS Catal.* **2019**, *9*, 10612–10617.

(S12) Giese, T. J.; Ekesan, Ş.; McCarthy, E.; Tao, Y.; York, D. M. Surface-Accelerated String Method for Locating Minimum Free Energy Paths. *J. Chem. Theory Comput.* **2024**, *20*, 2058–2073.

(S13) Ekesan, Ş.; York, D. M. Who stole the proton? Suspect general base guanine found with a smoking gun in the pistol ribozyme. *Org. Biomol. Chem.* **2022**, *20*, 6219–6230.

(S14) Ganguly, A.; Weissman, B. P.; Giese, T. J.; Li, N.-S.; Hoshika, S.; Rao, S.; Benner, S. A.; Piccirilli, J. A.; York, D. M. Confluence of theory and experiment reveals the catalytic mechanism of the Varkud satellite ribozyme. *Nat. Chem.* **2020**, *12*, 193–201.

(S15) Gaines, C. S.; Giese, T. J.; York, D. M. Cleaning Up Mechanistic Debris Generated by Twister Ribozymes Using Computational RNA Enzymology. *ACS Catal.* **2019**, *9*, 5803–5815.

(S16) Ekesan, Ş.; York, D. M. Dynamical ensemble of the active state and transition state mimic for the RNA-cleaving 8-17 DNAzyme in solution. *Nucleic Acids Res.* **2019**, *47*, 10282–10295.

(S17) Raines, R. T. Ribonuclease A. *Chem. Rev.* **1998**, *98*, 1045–1066.

(S18) Cech, T. R. Self-Splicing of Group I Introns. *Annu. Rev. Biochem.* **1990**, *59*, 543–568.

(S19) McCarthy, E.; Ekesan, Ş.; Giese, T. J.; Wilson, T. J.; Deng, J.; Huang, L.; Lilley, D. M. J.; York, D. M. Catalytic mechanism and pH dependence of a methyltransferase ribozyme (MTR1) from computational enzymology. *Nucleic Acids Res.* **2023**, *51*, 4508–4518.

(S20) Wilson, T. J.; McCarthy, E.; Ekesan, Ş.; Giese, T. J.; Li, N.-S.; Huang, L.; Piccirilli, J. A.; York, D. M.; Lilley, D. M. J. The Role of General Acid Catalysis in the Mechanism of an Alkyl Transferase Ribozyme. *ACS Catal.* **2024**, *14*, 15294–15305.

(S21) Giese, T. J.; Zeng, J.; Lerew, L.; McCarthy, E.; Tao, Y.; Ekesan, Ş.; York, D. M. Software Infrastructure for Next-Generation QM/MM−ΔMLP Force Fields. *J. Phys. Chem. B* **2024**, *128*, 6257–6271.

(S22) Puyo-Fourtine, J.; Du, Y.; McCarthy, E.; Ekesan, Ş.; York, D. M. Origins of reactivity in SAM-utilizing ribozyme SAMURI-catalyzed RNA alkylation. *bioRxiv* **2026**,
